# Epidemiological trends and geographic disparities in low back pain burden based on the 2021 GBD study: A cross-sectional analysis

**DOI:** 10.1097/MD.0000000000049201

**Published:** 2026-06-12

**Authors:** Jin Fu, Yuxin Ye, Yanlei Li, Yuan Zhang, Shanggao Xie, Jun Zhang, Tingxiao Zhao, Lijiang Tao

**Affiliations:** a Emergency and Critical Care Center, Department of Emergency Medicine, Zhejiang Provincial People’s Hospital, Affiliated People’s Hospital, Hangzhou Medical College, Hangzhou, Zhejiang, China; b The Second School of Clinical Medicine, Hangzhou Normal University, Hangzhou, Zhejiang, China; c Department of Rheumatology, The Second Affiliated Hospital of Zhejiang Chinese Medical University, Hangzhou, China; d Center for Plastic & Reconstructive Surgery, Department of Orthopedics, Zhejiang Provincial People’s Hospital, Affiliated People’s Hospital, Hangzhou Medical College, Hangzhou, Zhejiang, China.

**Keywords:** DALYs, GBD, incidence, low back pain, prevalence, SDI

## Abstract

Low back pain (LBP) is a global health concern with varying incidence across regions. Recognizing these regional differences is essential for creating effective prevention and control strategies. This study examines the burden and trends of LBP globally using recent data from the Global Burden of Diseases, Injuries, and Risk Factors Study (GBD). We extracted GBD data on prevalence, incidence, and disability-adjusted life years (DALYs) for LBP from 1990 to 2021 via the Global Health Data Exchange (GHDx). Crude and age-standardized rates (ASRs) and estimated annual percentage changes (EAPCs) were calculated globally, and by region, country, age, and sex. Decomposition, frontier, and cluster analyses were applied to identify influencing factors and trends. In 2021, there were 629 million (95% UI: 552–701 million) prevalent cases, 267 million (95% UI: 235–299 million) incident cases, and 70.16 million (95% UI: 65.76 to 83.22 million) DALYs due to LBP globally. From 1990 to 2021, ASRs of prevalence (EAPC = −0.32), incidence (EAPC = −0.29), and DALYs (EAPC = −0.32) declined. Central Europe and Hungary showed the highest burden, while Asia and China had the largest number of affected individuals. High Socio-demographic Index (SDI) regions exhibited the highest ASRs, whereas middle SDI regions had the lowest. Population growth and aging contributed to increasing case numbers, with burden peaking at ages 80–84 and being higher among females. Despite a slight decline in age-standardized rates, the global burden of LBP remains high, with marked regional and demographic disparities. Targeted health policies, particularly for elderly women in high-SDI regions, are essential to effectively reduce the burden of LBP worldwide.

## 1. Introduction

Reportedly, low back pain (LBP) has become the main cause of productivity loss globally and the leading contributor to disability worldwide.^[1–3]^ It is the most common musculoskeletal disorder globally and has become a global health challenge.^[4,5]^ Driven by a rapidly aging global population, the prevalence of LBP is projected to surge from 619 million cases in 2020 to 800 million by 2050,^[6]^ posing an escalating threat to healthcare systems, particularly in low- and middle-income countries where medical resources are often constrained.^[7]^ The socioeconomic burden of LBP is even more striking, exceeding US$100 billion annually in the United States alone,^[8]^ attributed to both direct healthcare costs and substantial productivity losses. The socioeconomic burden of LBP is expected to further increase in the coming decades.^[9,10]^ Chronic LBP arises from a complex interplay of biological, psychological, and social factors, often leading to depression, functional impairment, and premature workforce departure.^[11–13]^ These ramifications underscore the urgent need for updated epidemiological assessments to inform effective public health strategies.

The Global Burden of Diseases, Injuries, and Risk Factors Study (GBD) provides the largest and most comprehensive database for quantifying global and regional health trends. The COVID-19 pandemic has since caused a historic reversal in global life expectancy and severely disrupted healthcare systems,^[14]^ potentially creating a paradigm shift in the burden of conditions like LBP. Given this context, the GBD 2021 dataset is of particular importance; it is the first to integrate postpandemic health trends, thereby offering a unique opportunity to examine the contemporary burden of LBP.

Utilizing GBD 2021 data, this study delivers a comprehensive update on the global LBP burden from 1990 to 2021. Beyond presenting a global overview, we place specific emphasis on identifying and analyzing regions that exhibited distinctive epidemiological patterns in our preliminary analyses. The selection of these regions, characterized by either a persistently high, rapidly increasing, or notably declining burden of LBP, is motivated by their potential to reveal critical insights into the associated factors of disease trends. Their divergent trajectories are likely shaped by the complex interplay of population aging, socioeconomic development, disparities in healthcare resource allocation, and evolving lifestyle factors in the postpandemic context. Through this detailed analysis, we seek to generate reliable evidence to inform targeted health policies and resource allocation.

## 2. Methods

### 2.1. Overview

All of the data analyzed and presented in this article were obtained from the updated GBD 2021 (the Global Burden of Disease, Injuries, and Risk Factors Study) (http://www.healthdata.org/gbd/data).

The GBD 2021 study quantified the health loss of 371 diseases in 204 countries and regions worldwide, and for the first time explored the impact of the global COVID-19 pandemic, investigating the latest trends in global health. The GBD 2021 study used the latest epidemiological data and enhanced standardized methods to ensure consistency and comparability in measuring the disease burden across different populations and time periods. The study utilized 328,938 data sources, revealing health disparities across different age groups, genders, locations, and socioeconomic groups, and emphasizing the impact of the COVID-19 pandemic as well as other health challenges.

### 2.2. Data sources

This study retrieved data using the GHDx tool, selecting “LBP” as the research condition and using “prevalence,” “incidence,” and “DALYs” as evaluation indicators to analyze data across different regions, socioeconomic levels, genders, and age groups. In the GBD 2021 study, the Bayesian meta-regression modeling tool DisMod-MR 2.1, developed by the GBD research team, was used to fill in missing data and ensure result stability, thereby estimating the incidence and burden of diseases and injuries. DALYs measure health loss by combining years of life lost (YLLs) and years lived with disability (YLDs). As LBP has no mortality, YLDs and DALYs are identical. This article refers solely to DALYs. All data was directly extracted from the latest GBD database (updated in 2021). Each iteration of GBD reevaluates the entire time series in the light of new data and methods. Therefore, the results of GBD 2021 have replaced the results of previous GBD rounds. Since the GBD database is publicly available, this study did not require additional ethical review or exemption.

### 2.3. Statistical analyses

We assessed the burden of LBP by estimating the age-standardized incidence rates (ASIR), age-standardized prevalence rates (ASPR), and age-standardized DALYs rates (ASDR). The estimated annual percentage change (EAPC) was used to quantify ASIR, ASPR, and ASDR to understand trends in the global burden of LBP. All estimates are reported at a rate per 100,000 population and give a 95% uncertainty interval (UI). EAPC represents the natural logarithm of the regression line compliance rate, expressed as y = α + βx + ε, where y = ln (ASR), x = calendar year, and ε is the error. EAPC is reported with a 95% confidence interval (CI). If EAPC > 0, ASRs is rising; if EAPC < 0, ASRs is decreasing; otherwise, it remains stable. The EAPC analysis from 1990 to 2021 reflects the change trend of LBP burden over time.

To identify regions with similar temporal trends in disease burden, we performed hierarchical clustering analysis^[15]^ on their estimated EAPC. This method grouped the regions into 4 distinct clusters based on their trajectory patterns, which we categorized as “marked increase,” “mild increase,” “stable or mild decrease,” and “marked decrease.” Concurrently, regions were stratified according to the Socio-demographic Index (SDI), a composite indicator of income, education, and fertility, which is standardized on a scale from 0 to 1 and categorized into 5 quintiles: low, low-middle, middle, high-middle, and high.

The Das Gupta decomposition method^[16]^ was utilized to quantify the independent contributions of population growth, population aging, and epidemiological changes to the evolution of the LBP burden from 1990 to 2021. This demographic approach disentangles the effect of each factor while holding others constant, thereby clarifying the underlying associated factors of burden trends. Frontier analysis was applied to explore the nonlinear relationship between the SDI and disease burden. A smooth frontier, representing the optimal (lowest) ASDR for each SDI level, was established using locally weighted regression.^[17]^ The model’s robustness was ensured through 1000 bootstrap iterations, and different smoothing spans (0.3–0.5) were tested to capture the underlying nonlinear relationship reliably. The effective difference, calculated as the disparity between a region’s observed ASDR and this frontier, quantifies the potential for reducing the disease burden given its current level of development.

This study adheres to the Strengthening the Reporting of Observational Studies in Epidemiology guidelines for reporting cross-sectional studies.^[18]^ All data organization and analysis was performed using R software (version 4.3.1).

## 3. Results

### 3.1. Global level

In 2021, there were approximately 629 million (95% UI: 552-701 million) people with LBP globally, with a global ASPR of 7463.13 per 100,000 (95% UI: 6575.68–8321.8) (Table 1). A critical divergence characterized the temporal trend from 1990 to 2021: while the age-standardized rates (ASRs) for prevalence, incidence, and DALYs all exhibited significant annual declines (EAPC ≈ −0.30 for all), the absolute number of prevalent cases, incident cases, and DALYs increased markedly, by over 60% in each category (Table 1, Figure S1, Supplemental Digital Content). This underscores that the public health challenge of LBP is growing in absolute terms, which is associated with population growth and aging, even as the risk-adjusted rates improve.

**Table 1:** Prevalent cases, incident cases, and DALYs cases of LBP in 1990 and 2021, along with ASRs and EAPC.

	Location	1990		2021		EAPC (95% CI) 1990-2021
		Cases (95% UI)	ASRs per 100 000 (95% UI)	Cases (95% UI)	ASRs per 100 000 (95% UI)	
Prevelence	Global	386,731,361 (341,581,662-434,164,620)	8391.58 (7381.14-9367.39)	628,838,475 (551,834,407-700,881,341)	7463.13 (6575.68-8321.8)	−0.32 (−0.35 to −0.28)
	Sex					
	Female	242,102,830 (214,937,265-271,065,504)	10,272.63 (9053.81-11,491.92)	396,747,972 (348,340,471-442,511,662)	9212.46 (8122.92-10,285.14)	−0.28 (−0.32 to −0.24)
	Male	144,628,531 (126,723,499-163,120,515)	6393.26 (5615.58-7160.79)	232,090,503 (203,351,350-259,931,953)	5640.23 (4965.63-6300.61)	−0.36 (−0.38 to −0.34)
	SDI region					
	High-middle SDI	93,734,190 (82,340,884-105,198,177)	8897.05 (7815.47-9917.76)	128,827,069 (113,031,393-144,240,276)	7631.54 (6693.46-8509.81)	−0.43 (−0.47 to −0.38)
	High SDI	105,597,928 (94,355,803-117,381,974)	10,585.74 (9467.02-11,806.57)	143,792,047 (129,652,681-157,076,124)	9783.64 (8876.62-10,734.23)	−0.19 (−0.21 to −0.16)
	Low-middle SDI	65,025,917 (57,621,208-73,772,912)	7762.41 (6800.92-8691.74)	126,191,233 (110,548,447-142,904,183)	7317.71 (6386.63-8227.13)	−0.18 (−0.23 to −0.14)
	Low SDI	25,218,245 (22,268,893-28,454,461)	7841.48 (6870.11-8781.4)	55,999,862 (49,426,590-63,405,672)	7364.87 (6427.17-8255.87)	−0.21 (−0.23 to −0.19)
	Middle SDI	96,691,431 (85,105,762-109,488,201)	7044.2 (6156.41-7905.07)	173,417,025 (150,668,277-195,125,730)	6421.04 (5616.75-7199.09)	−0.2 (−0.25 to −0.15)
Incidence	Global	165,063,882 (145,785,270-185,933,884)	3534.99 (3133.04-3960.99)	266,873,321 (235,369,489-299,406,380)	3176.63 (2811.82-3562.29)	−0.29 (−0.32 to −0.26)
	Sex					
	Female	101,510,307 (90,057,184-114,008,585)	4263.66 (3774.25-4776.07)	166,097,403 (146,916,668-186,192,888)	3879.94 (3438.63-4344.9)	−0.24 (−0.28 to −0.2)
	Male	63,553,575 (55,765,751-71,904,401)	2770.8 (2441.21-3122.36)	100,775,918 (88,485,948-113,863,884)	2450.55 (2161.03-2758.87)	−0.35 (−0.38 to −0.33)
	SDI region					
	High-middle SDI	39,553,208 (35,006,678-44,407,273)	3734.53 (3318.15-4178.19)	54,050,238 (47,424,129-60,795,832)	3250.38 (2870.7-3648.88)	−0.38 (−0.42 to −0.33)
	High SDI	43,722,165 (39,062,418-49,065,394)	4424.22 (3944.67-4976.02)	59,095,839 (52,994,576-65,248,529)	4118.8 (3695.02-4589.31)	−0.17 (−0.19 to −0.14)
	Low-middle SDI	28,310,723 (24,878,028-32,058,294)	3289.8 (2902.59-3692.6)	54,298,819 (47,620,836-61,354,906)	3113.93 (2740.21-3499.77)	−0.18 (−0.22 to −0.13)
	Low SDI	11,039,527 (9,652,870-12,437,704)	3315.99 (2931.45-3721.91)	24,608,330 (21,598,995-27,916,670)	3134.89 (2760.73-3519.33)	−0.19 (−0.21 to −0.17)
	Middle SDI	42,247,526 (37,048,076-47,853,836)	3016.06 (2660.6-3392.23)	74,568,788 (65,108,174-84,107,629)	2770.57 (2435.96-3119.83)	−0.19 (−0.24 to −0.15)
DALYs	Global	43,386,226 (31,083,937-58,355,210)	937.34 (669.13-1261)	70,156,962 (50,194,205-94,104,688)	832.18 (595.85-1115.24)	−0.32 (−0.35 to −0.28)
	Sex					
	Female	26,974,140 (19,377,113-36,203,736)	1142.28 (817.03-1533.18)	43,934,955 (31,447,685-58,945,143)	1021.52 (732.39-1370.45)	−0.28 (−0.32 to −0.24)
	Male	16,412,085 (11,713,623-22,118,911)	720.6 (512.4-974.5)	26,222,007 (18,702,285-35,396,348)	635.48 (453.91-854.29)	−0.35 (−0.38 to −0.33)
	SDI region					
	High-middle SDI	10,524,244 (7,567,872-14,235,162)	995.35 (712.29-1343.18)	14,394,112 (10,223,002-19,435,230)	854.81 (611.67-1147)	−0.41 (−0.46 to −0.37)
	High SDI	11,851,271 (8,465,650-15,920,115)	1189.91 (854.78-1600.27)	15,937,890 (11,551,525-21,216,646)	1094.34 (792.26-1459.42)	−0.2 (−0.23 to −0.17)
	Low-middle SDI	7,245,518 (5,146,960-9,671,477)	857.76 (613.87-1152.2)	14,064,118 (10,073,508-18,918,257)	810.23 (580.5-1088.22)	−0.17 (−0.22 to −0.12)
	Low SDI	2,801,498 (1,997,111-3,731,852)	863.78 (617.16-1157.85)	6,254,664 (4,446,006-8,373,290)	815.25 (582.63-1097.15)	−0.18 (−0.2 to −0.16)
	Middle SDI	10,911,753 (7,780,236-14,604,346)	788.71 (560.36-1061.61)	19,437,989 (13,849,605-26,187,075)	717.45 (512.59-962.53)	−0.2 (−0.25 to −0.15)

ASRs = age-standardized rates, CI = confdence interval, DALYs = disability-adjusted life years, EAPC = estimated annual percentage change, LBP = low back pain, SDI = social development index, UI = uncertainty interval.

### 3.2. Regional and national levels

**Figure 1.**
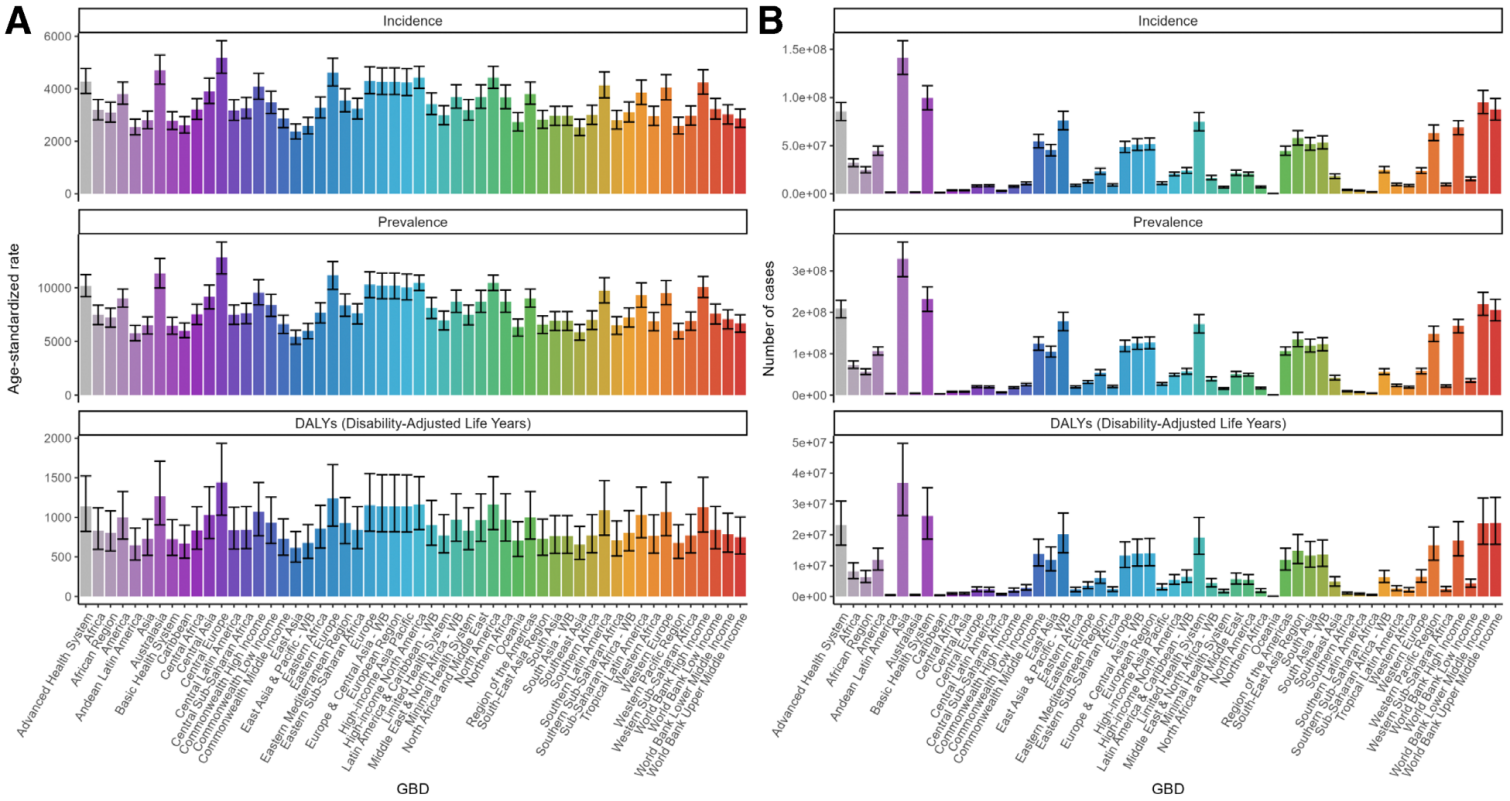
The regional burden of LBP in 2021. (A) Age-standardized rates show the highest burden in Central Europe, Australasia, and Eastern Europe. (B) Absolute case counts were greatest in Asia due to its large population.

**Figure 2.**
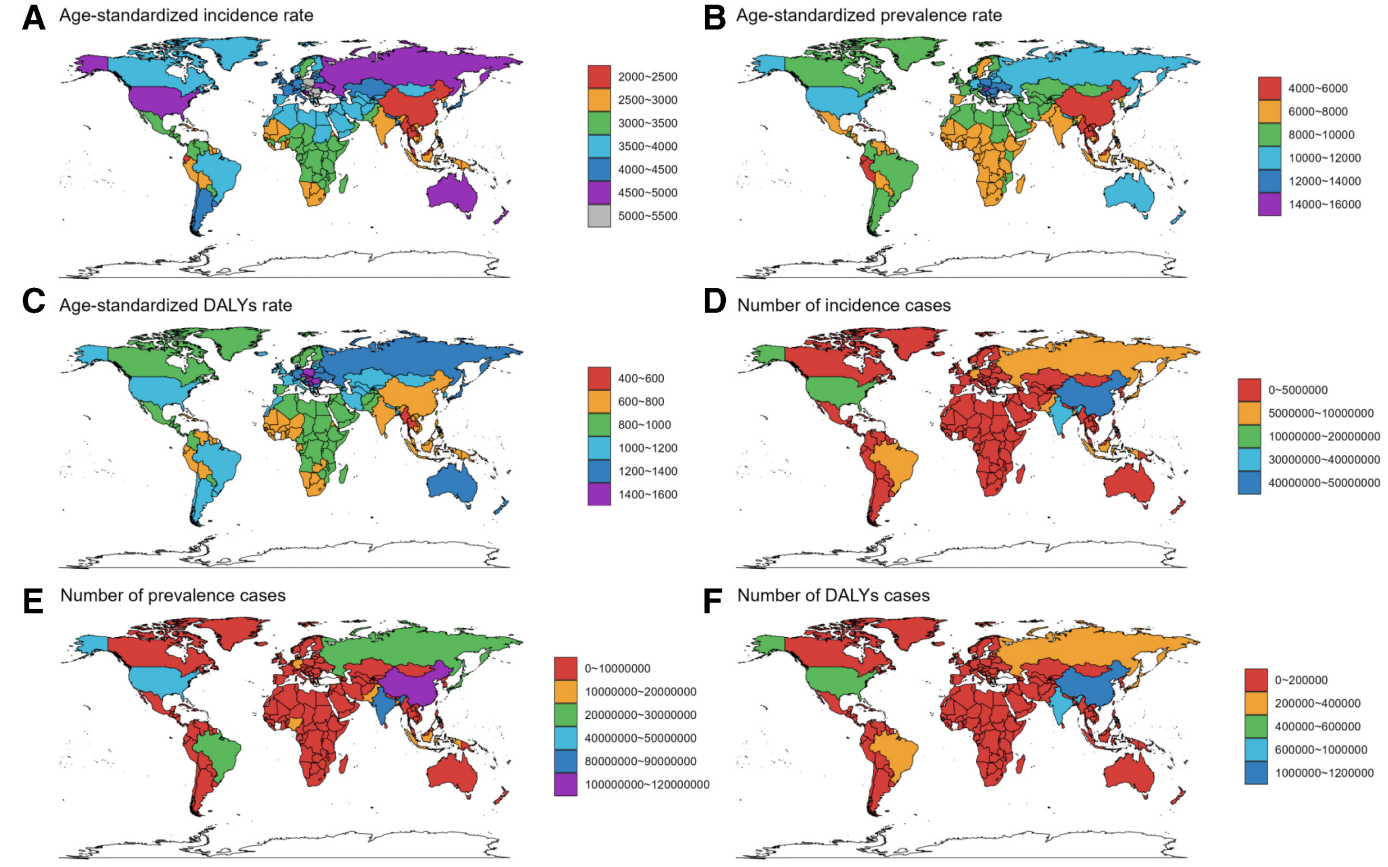
National estimates of low back pain burden in 2021. Maps show (A) age-standardized incidence rate (ASIR), (B) age-standardized prevalence rate (ASPR), (C) age-standardized DALYs rate (ASDR), and absolute numbers of (D) incident cases, (E) prevalent cases, and (F) DALYs.

In 2021, Central Europe, Australasia, and Eastern Europe consistently exhibited the highest ASRs for prevalence, incidence, and DALYs of low back pain (Fig. 1A, Tables S1–S3, Supplemental Digital Content). In contrast, the age-standardized burden was relatively low in East Asia, Southeast Asia, and Andean Latin America. This spatial pattern was replicated at the national level, with Hungary, Czechia, and Poland recording the highest ASRs globally (Fig. 2A–2C, Tables S6, S7, Supplemental Digital Content), while countries in East and Southeast Asia, along with nations like the Maldives and Myanmar, reported the lowest.

Diverging from the ASRs patterns, Asia and the World Bank lower-middle-income region accounted for the largest share of global prevalent cases, incident cases, and DALYs due to their substantial population bases (Fig. 1B). Asia alone was responsible for 329 million prevalent cases. Consequently, China and India shouldered the greatest absolute burden, recording 43.37 million and 38.58 million new cases in 2021 (Fig. 2D), respectively, while also ranking highest in total patients and disability-adjusted life years (DALYs) during the same year (Fig. 2E–2F, Table S4, Supplemental Digital Content).

**Figure 3.**
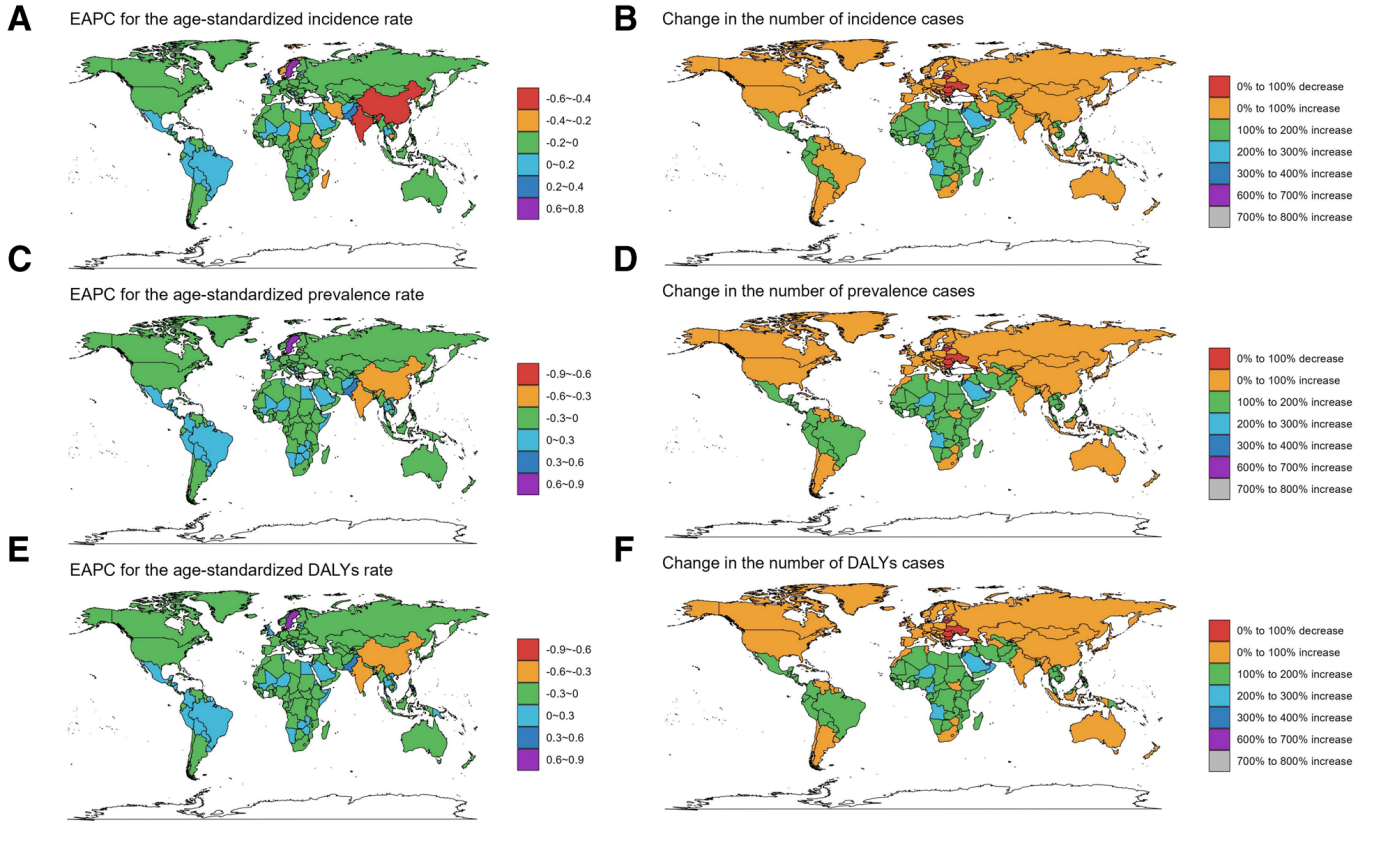
Trends in the global burden of low back pain, 1990–2021. Panels show the estimated annual percentage change (EAPC) for (A) ASIR, (C) ASPR, (E) ASDR, and the corresponding percentage change in absolute numbers for (B) incident cases, (D) prevalent cases, (F) DALYs

From 1990 to 2021, a majority of regions and countries experienced declining ASRs (Tables S1–S3, Supplemental Digital Content). The most pronounced decreases were observed in the Western Pacific Region, East Asia, and the East Asia & Pacific region. However, an increasing trend in the burden was noted in several nations, particularly in Latin America (e.g., Tropical Latin America) and the Eastern Mediterranean. Notably, Sweden, Taiwan (Province of China), and Pakistan registered the largest increases in ASIR, whereas Denmark, China, and India saw the most significant declines (Fig. 3A, Table S4, Supplemental Digital Content). Compared to 1990, the ASPR and ASDR in most countries and regions worldwide have seen varying degrees of increase (Figs. 3C, 3E). In absolute terms, Gulf Cooperation Council (GCC) countries, including Qatar and the United Arab Emirates, experienced the most dramatic growth, with the number of incident cases, prevalent cases, and DALYs each increasing by over 300% (Fig. 3B, 3D, 3F, Table S5, Supplemental Digital Content). Based on these trends, a stratified clustering method was used to categorize the burden of LBP across all GBD regions into 4 groups (Figure S2, Supplemental Digital Content).

### 3.3. Age and sex patterns of LBP

**Figure 4.**
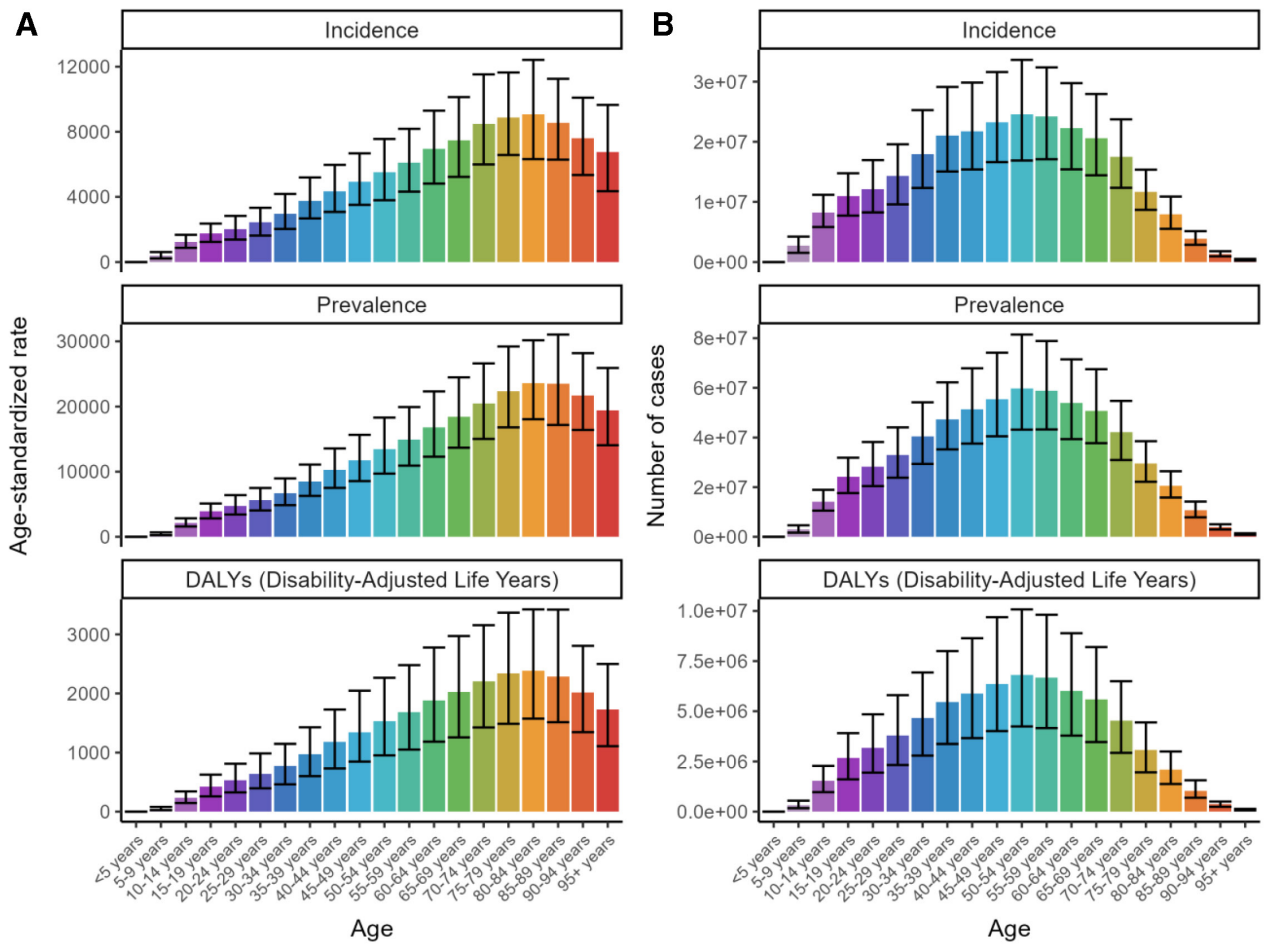
Age-specific patterns of low back pain burden in 2021. (A) Age-standardized rates (ASRs) peaked in the 80-84 year age group. (B) The absolute number of cases was highest in the 50-54 year age group. Error bars represent 95% uncertainty intervals.

In 2021, globally, the ASIR of LBP is lowest in children aged 9 and below, increasing with age until peaking at 80-84 years old (Fig. 4A, Table 2). This pattern is also observed in ASPR and ASDR. However, the 50-54 age group has the highest number of burdened individuals (Fig. 4B). From 1990 to 2021, trend analysis reveals a slight increase in ASIR for those aged 95 and above, while all other age groups show varying degrees of decrease in ASIR, ASPR, and ASDR (EAPC < 0) (Figure S3, Supplemental Digital Content, Table 2). Compared to 1990, there was a noticeable upward trend in patients with the LBP burden across all age groups in 2021. Globally, the ASRs and number of patients with LBP were higher in women than in men in 2021, suggesting that its impact is more pronounced in women (Figure S4, Supplemental Digital Content, Table 1). Moreover, over the past 30 years, while the ASIR, ASPR, and ASDR have declined, they remain higher than those for men, leaving women with a greater burden of LBP (Figure S5, Supplemental Digital Content).

**Table 2:** Global age distribution of prevalent cases, incident cases, and DALYs due to LBP in 1990 and 2021, including ASRs and EAPC.

Location	1990		2021		EAPC (95% CI) 1990-2021
	Cases (95% UI)	ASRs per 100 000 (95% UI)	Cases (95% UI)	ASRs per 100 000 (95% UI)	
**Prevalence**					
5–9 years	2,595,082 (1,431,890-4,069,488)	444.72 (245.38-697.39)	2,958,719 (1,659,796-4,628,651)	430.64 (241.58-673.7)	-0.07 (-0.1 to -0.04)
10–14 years	11,920,254 (8,797,881-15,886,526)	2225.25 (1642.37-2965.66)	14,178,912 (10,531,791-18,925,845)	2126.93 (1579.84-2839.01)	-0.15 (-0.17 to -0.14)
15–19 years	21,356,546 (15,422,303-28,134,067)	4111.59 (2969.12-5416.4)	24,297,914 (17,619,300-31,876,412)	3894.02 (2823.7-5108.56)	-0.23 (-0.26 to -0.2)
20–24 years	24,891,379 (17,932,171-33,859,071)	5058.32 (3644.1-6880.7)	28,264,043 (20,432,758-38,206,774)	4733.09 (3421.67-6398.1)	-0.23 (-0.28 to -0.19)
25–29 years	27,605,244 (19,871,555-36,872,090)	6236.79 (4489.53-8330.42)	32,974,313 (23,808,037-44,101,022)	5604.61 (4046.62-7495.8)	-0.24 (-0.3 to -0.17)
30–34 years	30,234,755 (21,708,142-40,681,785)	7844.57 (5632.3-10,555.11)	40,469,160 (29,368,915-54,200,102)	6694.87 (4858.54-8966.4)	-0.33 (-0.43 to -0.23)
35–39 years	34,265,988 (25,575,536-44,820,626)	9727.9 (7260.73-12,724.3)	47,385,924 (35,227,212-62,200,742)	8448.71 (6280.86-11,090.12)	-0.35 (-0.44 to -0.27)
40–44 years	33,481,062 (24,247,395-44,165,413)	11,686.98 (8463.86-15,416.49)	51,400,903 (37,564,314-67,867,421)	10,275.03 (7509.1-13,566.68)	-0.38 (-0.44 to -0.31)
45–49 years	30,722,253 (22,260,319-40,895,295)	13,231.2 (9586.89-17,612.44)	55,462,913 (40,506,735-74,148,990)	11,713.28 (8554.67-15,659.61)	-0.37 (-0.4 to -0.33)
50–54 years	32,652,367 (23,553,166-44,296,273)	15,360.65 (11,080.12-20,838.3)	59,765,618 (43,183,511-81,457,391)	13,432.8 (9705.84-18,308.2)	-0.36 (-0.39 to -0.33)
55–59 years	31,259,590 (22,844,127-41,798,218)	16,878.83 (12,334.84-22,569.24)	58,963,609 (43,259,709-78,865,827)	14,900.03 (10,931.68-19,929.3)	-0.32 (-0.35 to -0.28)
60–64 years	30,173,774 (21,789,776-39,882,728)	18,787.07 (13,566.95-24,832.15)	53,862,394 (39,362,646-71,471,628)	16,829.48 (12,298.99-22,331.54)	-0.31 (-0.34 to -0.27)
65–69 years	25,720,212 (18,961,993-34,271,717)	20,807.64 (15,340.24-27,725.81)	50,825,855 (37,740,718-67,494,985)	18,425.7 (13,682-24,468.7)	-0.34 (-0.37 to -0.31)
70–74 years	19,390,757 (14,169,188-25,408,845)	22,903.96 (16,736.35-30,012.4)	42,173,146 (30,943,170-54,775,717)	20,488.39 (15,032.69-26,610.92)	-0.35 (-0.37 to -0.33)
75–79 years	16,076,199 (12,115,415-20,992,687)	26,116.59 (19,682.1-34,103.67)	29,508,893 (22,168,668-38,524,820)	22,374.82 (16,809.17-29,211.05)	-0.35 (-0.39 to -0.31)
80–84 years	9,326,697 (7,069,414-11,888,956)	26,364.53 (19,983.69-33,607.48)	20,648,665 (15,816,087-26,419,482)	23,576.09 (18,058.39-30,165.06)	-0.31 (-0.35 to -0.27)
85–89 years	3,886,563 (2,835,673-5,138,068)	25,719.97 (18,765.53-34,002.01)	10,750,479 (7,853,607-14,192,772)	23,512.81 (17,176.94-31,041.59)	-0.23 (-0.26 to -0.2)
90–94 years	973,059 (729,208-1,265,430)	22,707.5 (17,016.95-29,530.32)	3,888,465 (2,935,704-5,042,620)	21,736.17 (16,410.32-28,187.79)	-0.14 (-0.15 to -0.12)
95 + years	199,577 (142,259-268,595)	19,603.18 (13,973.15-26,382.38)	1,058,551 (766,407-1,412,142)	19,421.86 (14,061.72-25,909.39)	-0.05 (-0.08 to -0.02)
Incidence					
5–9 years	2,367,823 (1,339,357-3,721,818)	405.78 (229.53-637.81)	2,701,534 (1,539,114-4,236,548)	393.21 (224.02-616.63)	-0.07 (-0.09 to -0.04)
10–14 years	6,969,757 (4,913,224-9,435,889)	1301.1 (917.19-1761.47)	8,256,822 (5,834,978-11,174,091)	1238.58 (875.29-1676.19)	-0.17 (-0.19 to -0.16)
15–19 years	9,707,953 (6,856,673-13,042,832)	1868.99 (1320.05-2511.02)	10,977,630 (7,733,059-14,758,035)	1759.29 (1239.31-2365.15)	-0.23 (-0.28 to -0.19)
20–24 years	10,745,129 (7,253,269-14,933,219)	2183.58 (1473.98-3034.67)	12,120,793 (8,254,564-16,947,345)	2029.75 (1382.31-2838)	-0.23 (-0.29 to -0.17)
25–29 years	12,079,579 (8,074,245-16,512,476)	2729.11 (1824.19-3730.62)	14,344,676 (9,580,873-19,580,295)	2438.15 (1628.45-3328.04)	-0.24 (-0.31 to -0.18)
30–34 years	13,399,926 (9,086,662-18,915,793)	3476.69 (2357.58-4907.81)	17,969,817 (12,312,377-25,264,619)	2972.77 (2036.85-4179.56)	-0.34 (-0.43 to -0.25)
35–39 years	15,063,946 (10,743,987-20,827,583)	4276.56 (3050.15-5912.82)	21,064,444 (15,049,925-29,122,559)	3755.7 (2683.34-5192.43)	-0.33 (-0.41 to -0.26)
40–44 years	13,951,113 (9,815,751-19,085,918)	4869.81 (3426.31-6662.18)	21,753,719 (15,392,009-29,847,567)	4348.56 (3076.86-5966.52)	-0.33 (-0.39 to -0.28)
45–49 years	12,706,720 (9,069,576-17,309,631)	5472.42 (3906.01-7454.77)	23,262,660 (16,610,856-31,602,484)	4912.87 (3508.07-6674.17)	-0.33 (-0.36 to -0.3)
50–54 years	13,278,029 (9,138,519-18,043,728)	6246.38 (4299.03-8488.31)	24,565,382 (16,878,271-33,626,265)	5521.27 (3793.53-7557.77)	-0.33 (-0.36 to -0.3)
55–59 years	12,663,226 (8,980,302-16,987,476)	6837.6 (4848.98-9172.51)	24,203,870 (17,094,038-32,376,054)	6116.29 (4319.64-8181.39)	-0.28 (-0.31 to -0.26)
60–64 years	12,329,764 (8,525,444-16,608,759)	7676.87 (5308.19-10,341.1)	22,287,196 (15,409,747-29,753,144)	6963.71 (4814.83-9296.47)	-0.28 (-0.3 to -0.25)
65–69 years	10,352,327 (7,235,502-13,953,614)	8375.03 (5853.52-11,288.47)	20,630,766 (14,421,113-27,947,621)	7479.19 (5228.03-10,131.74)	-0.32 (-0.35 to -0.3)
70–74 years	7,971,749 (5,531,252-10,987,895)	9416.06 (6533.4-12,978.67)	17,457,195 (12,335,067-23,728,059)	8480.99 (5992.57-11,527.47)	-0.33 (-0.34 to -0.31)
75–79 years	6,216,952 (4,643,308-8,179,183)	10,099.75 (7543.29-13,287.49)	11,696,358 (8,668,578-15,356,227)	8868.64 (6572.86-11,643.7)	-0.3 (-0.33 to -0.26)
80–84 years	3,491,646 (2,426,263-4,787,917)	9870.12 (6858.52-13,534.39)	7,945,436 (5,535,297-10,876,982)	9071.89 (6320.05-12,419.05)	-0.22 (-0.25 to-0.19)
85–89 years	1,366,377 (995,612-1,803,115)	9042.22 (6588.62-11,932.41)	3,903,147 (2,874,618-5,145,849)	8536.73 (6287.19-11,254.7)	-0.13 (-0.15 to -0.11)
90–94 years	333,695 (232,465-445,834)	7787.18 (5424.85-10,404.07)	1,362,947 (955,907-1,806,151)	7618.75 (5343.44-10,096.22)	-0.04 (-0.07 to -0.01)
95 + years	68,171 (43,056-98,657)	6695.97 (4229.13-9690.46)	368,926 (236,948-525,990)	6768.91 (4347.42-9650.65)	0.04 (-0.01-0.09)
DALYs					
5–9 years	282,652 (148,420-483,110)	48.44 (25.43-82.79)	322,999 (172,035-546,268)	47.01 (25.04-79.51)	-0.06 (-0.09 to -0.04)
10–14 years	1,291,279 (809,976-1,918,014)	241.05 (151.2-358.05)	1,537,405 (970,908-2,280,861)	230.62 (145.64-342.14)	-0.15 (-0.16 to -0.13)
15–19 years	2,345,953 (1,418,741-3,429,036)	451.65 (273.14-660.16)	2,668,590 (1,610,051-3,906,595)	427.67 (258.03-626.08)	-0.23 (-0.26 to -0.2)
20–24 years	2,811,759 (1,717,189-4,278,071)	571.39 (348.96-869.37)	3,192,076 (1,943,526-4,845,422)	534.54 (325.46-811.41)	-0.23 (-0.27 to -0.18)
25–29 years	3,171,326 (1,932,993-4,865,643)	716.49 (436.72-1099.28)	3,785,697 (2,324,087-5,800,596)	643.45 (395.02-985.92)	-0.23 (-0.29 to -0.16)
30–34 years	3,485,824 (2,083,958-5,187,977)	904.42 (540.69-1346.05)	4,662,317 (2,793,017-6,932,084)	771.29 (462.05-1146.78)	-0.32 (-0.42 to -0.23)
35–39 years	3,950,186 (2,438,495-5,782,981)	1121.43 (692.27-1641.75)	5,455,697 (3,370,953-7,999,316)	972.73 (601.03-1426.24)	-0.35 (-0.43 to -0.26)
40–44 years	3,835,723 (2,382,455-5,651,541)	1338.91 (831.63-1972.74)	5,880,450 (3,658,350-8,639,811)	1175.5 (731.3-1727.1)	-0.37 (-0.43 to -0.31)
45–49 years	3,527,882 (2,237,832-5,388,179)	1519.36 (963.77-2320.54)	6,364,181 (4,010,153-9,688,965)	1344.06 (846.91-2046.22)	-0.36 (-0.39 to -0.33)
50–54 years	3,726,950 (2,320,970-5,512,378)	1753.27 (1091.85-2593.19)	6,816,592 (4,239,946-10,077,366)	1532.08 (952.96-2264.97)	-0.36 (-0.39 to -0.32)
55–59 years	3,543,076 (2,211,275-5,187,128)	1913.11 (1193.99-2800.83)	6,673,088 (4,158,522-9,810,461)	1686.28 (1050.85-2479.09)	-0.31 (-0.35 to -0.28)
60–64 years	3,375,354 (2,108,627-5,024,034)	2101.59 (1312.89-3128.11)	6,011,465 (3,784,032-8,889,762)	1878.3 (1182.33-2777.64)	-0.31 (-0.34 to -0.27)
65–69 years	2,829,635 (1,744,611-4,174,415)	2289.17 (1411.39-3377.1)	5,577,566 (3,462,952-8,198,946)	2022.01 (1255.41-2972.33)	-0.34 (-0.37 to -0.31)
70–74 years	2,092,095 (1,336,220-3,017,159)	2471.14 (1578.32-3563.81)	4,538,764 (2,930,586-6,495,869)	2205 (1423.73-3155.8)	-0.35 (-0.37 to -0.33)
75–79 years	1,683,248 (1,066,171-2,434,743)	2734.52 (1732.05-3955.36)	3,080,131 (1,958,825-4,441,639)	2335.48 (1485.26-3367.83)	-0.36 (-0.4 to -0.31)
80–84 years	946,714 (627,119-1,359,475)	2676.15 (1772.73-3842.94)	2,090,832 (1,377,472-2,998,239)	2387.26 (1572.76-3423.31)	-0.31 (-0.35 to -0.27)
85–89 years	378,421 (248,736-566,697)	2504.26 (1646.05-3750.21)	1,044,192 (691,417-1,563,592)	2283.79 (1512.23-3419.8)	-0.24 (-0.27 to -0.21)
90–94 years	90,365 (59,471-125,999)	2108.78 (1387.82-2940.34)	360,624 (240,426-502,027)	2015.85 (1343.96-2806.28)	-0.14 (-0.16 to -0.12)
95 + years	17,783 (11,214-25,828)	1746.72 (1101.5-2536.89)	94,298 (60,415-136,199)	1730.14 (1108.47-2498.92)	-0.05 (-0.08 to -0.02)

ASRs = age-standardized rates, CI = confdence interval, DALYs = disability-adjusted life years, EAPC = estimated annual percentage change, LBP = low back pain, SDI = social development index, UI = uncertainty interval.

### 3.4. Burden of LBP by socio-demographic index

In 2021, regions with high Social Demographic Index (SDI) had the highest ASIR at 3250.38 (95% UI: 2870.7-3648.88) per 100,000 people, while those with middle SDI had the lowest rate at 2770.57 (95% UI: 2435.96-3119.83) (Table 1, Figure S6, Supplemental Digital Content). The same pattern was observed in ASPR and ASDR. Despite lower ASIR, ASPR, and ASDR in middle SDI region, their number of cases were higher compared to other SDI regions (Table 1, Figure S6, Supplemental Digital Content). From 1990 to 2021, all SDI regions showed a decreasing trend in ASIR, ASPR, and ASDR, while the affected population increased (Figure S7, Supplemental Digital Content). Among them, the high-middle SDI region had the largest decrease in incidence, prevalence, and DALYs, with EAPCs of −0.43 (95% CI: −0.47 to −0.38), −0.38 (95% CI: −0.42 to −0.33), and −0.41 (95% CI: −0.46 to −0.37), respectively (Table 1, Figure S7, Supplemental Digital Content).

### 3.5. Decomposition analysis of LBP burden

**Figure 5.**
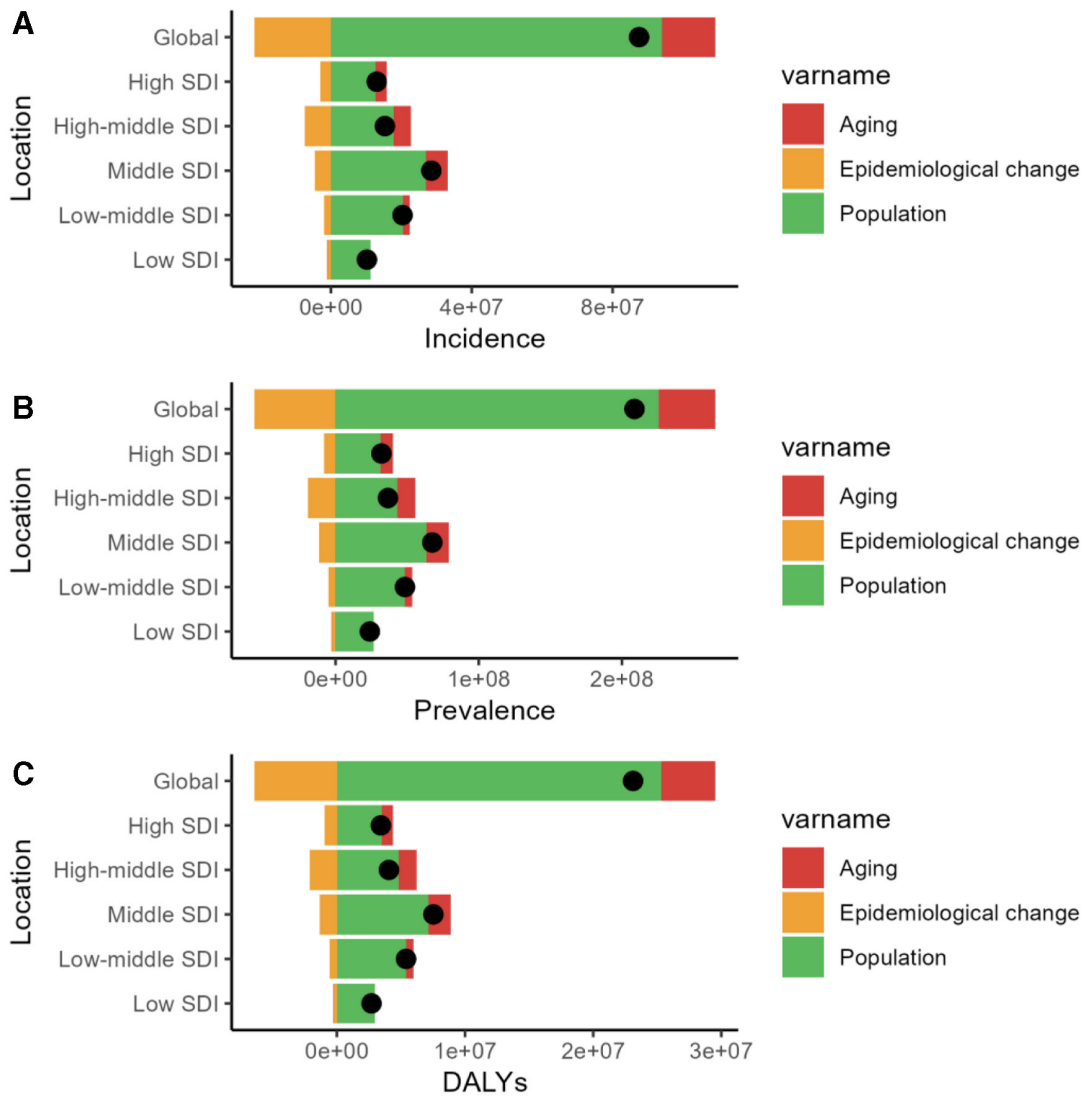
Decomposition analysis of associated factors for the increase in low back pain burden, 1990–2021. Contributions of population growth, aging, and epidemiological changes to changes in (A) incidence, (B) prevalence, and (C) DALYs, globally and by SDI quintile. The black dot denotes the net change. Positive values indicate an increase attributable to that associated factor.

Decomposition analysis revealed that from 1990 to 2021, global increases in LBP incidence were primarily associated with population growth (108.14%), followed by population aging (18.89%), while epidemiological changes partially offset this rise (Fig. 5A–5C, Table S8, Supplemental Digital Content). Population growth had the strongest effect in the high-middle SDI quintile (117.49%), and was the main associated factor for increases in prevalence and DALYs globally and across all SDI regions. The high-middle SDI region also experienced the largest increases in prevalence and DALYs associated with combined demographic forces, whereas the association of epidemiological changes with burden reduction was most evident in these regions.

### 3.6. Frontier analysis of LBP burden

**Figure 6.**
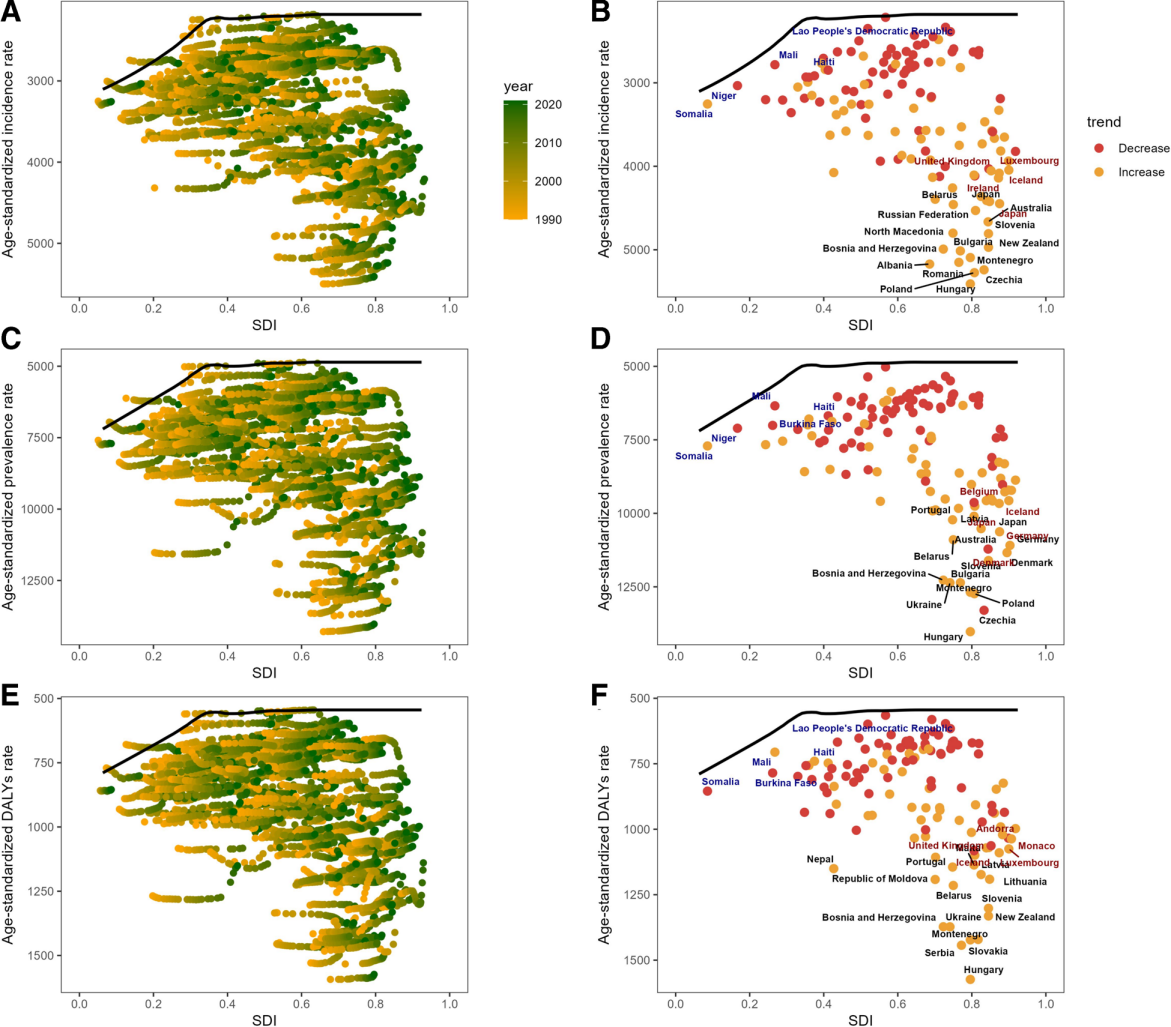
Frontier analysis of LBP burden vs Socio-demographic Index (SDI). The black line represents the theoretical minimum burden at each SDI level. The vertical distance of a point from this line (effective difference) indicates unrealized potential for burden reduction. In 2021, high-SDI countries (e.g., Hungary, Poland) showed the largest effective differences. Colors in A, C, E indicate the progression of years (1990-2021); in B, D, F, red/yellow dots indicate decreasing/increasing ASRs since 1990. Countries with low SDI (< 0.5) and minimal deviation from the frontier are highlighted in blue, while those with high SDI (> 0.78) and notable deviation for their developmental level are emphasized in red.

Based on the frontier analysis, we examined the correlation between the global burden of LBP and socio-demographic development from 1990 to 2021 (Figs. 6A-6F). The results indicate a positive association between SDI and ASIR, ASPR, and ASDR. The “effective difference”, reflecting the gap between observed rates and the theoretical optimum for a given SDI, was largest in high-SDI countries such as Hungary, Poland, and Czechia, indicating considerable potential for improvement. In contrast, several lower-SDI countries, including Maldives, Mali, and Timor-Leste, performed closer to the ideal frontier. Finally, in the ASDR analysis, countries like Malaysia, Sri Lanka, Somalia, Eswatini, and Mauritius performed closer to the ideal standard.

## 4. Discussion

This study used GBD 2021 data to study the change of global LBP trend from 1990 to 2021 and the global prevalence status. Our analysis confirms that LBP remains a formidable public health challenge, with an estimated 629 million prevalent cases and 70 million DALYs in 2021. A critical finding is the striking disparity between the overall increase in absolute case numbers and the observed decline in ASRs. This divergence suggests that the growing burden of LBP is primarily attributable to demographic transitions, specifically population growth and aging, despite the notable age and sex variations in its distribution. Furthermore, a general positive correlation was observed between the LBP burden and the SDI. This nuanced understanding is crucial for guiding policy responses that extend beyond disease management to address fundamental demographic trends.

Our results align with those of Wang et al,^[19]^ who observed a global decline in age-standardized LBP rates from 1990 to 2019 alongside significant regional heterogeneity and an increasing absolute burden driven by population aging and growth. This study extends the analysis through 2021, including the first postpandemic estimates, and confirms the persistence of these trends. Using GBD 2021 data, Cheng et al^[20]^ further projected that the LBP burden will continue to rise through 2050, especially in low- and middle-income countries, highlighting the need for preventive action. Our findings complement these projections by offering a decomposition and frontier analysis that clarifies how demographic factors and developmental disparities shape the current burden.

It is noteworthy that GCC countries account for half of the top ten countries with the highest number of patients growth in the burden of LBP. Among them, Qatar and the United Arab Emirates are the countries with the most pronounced global trend in the growth of the burden of LBP, consistent with previous research findings.^[21]^ While social and economic development and improved living conditions have occurred, they may have also brought about unhealthy lifestyles such as obesity, sedentary behavior, and lack of exercise.^[22–24]^ Previous studies have shown that physical inactivity is a risk factor for various diseases, and the relationship is more pronounced in high-income countries.^[24]^ Nevertheless, some low-middle income countries are also among the nations experiencing a faster increase in the burden of LBP, which may be related to differences in socioeconomic and healthcare systems.^[25]^ In addition, occupation and smoking have also been shown to be closely related to the occurrence and development of LBP.^[26,27]^ Therefore, addressing modifiable risk factors, such as smoking cessation, promotion of physical activity, and reduction of sedentary behavior, must be a primary focus in these regions, particularly as these issues are prevalent in affluent, urbanized settings. Concurrently, healthcare systems must be adapted to prioritize resource allocation towards public health education, routine screenings, and early intervention strategies. This systematic shift is essential to alleviate the growing burden of low back pain and prevent long-term disability.

As the research indicates, there are significant gender and age differences in the global patterns of LBP burden. In 2021, the burden of LBP was higher in females compared to males, consistent with the findings of the previous 2 GBD studies.^[28]^ Over the past 3 decades, the burden of LBP has consistently been higher in females, especially in middle-aged and older women. Research has shown that female hormones play a crucial role in preventing various musculoskeletal diseases, with a significant increase in disease incidence after menopause.^[29]^ From a physical and mental perspective, women are more sensitive to pain, more susceptible to musculoskeletal diseases, and more willing to report pain.^[30,31]^ Additionally, LBP is closely related to degenerative changes in intervertebral discs, which worsen with age, partly explaining its age-related pattern.^[32,33]^ On the other hand, aging is closely associated with chronic musculoskeletal pain, and older women are more vulnerable to the effects of osteoporosis and fractures.^[34,35]^ Consequently, targeted policy actions are imperative. First, integrate spine health into women’s health programs, with routine screening for middle-aged and older women. Second, develop scalable community-based exercise programs, such as adapted yoga, to improve muscle strength and proprioception. Finally, leverage digital tools for personalized education and follow-up, creating an integrated “prevention-intervention-management” continuum. Implementing this multi-pronged strategy is critical for reducing the impact of LBP on women.

With the continuous improvement of economic globalization and medical care system, the incidence, prevalence and DALYs of LBP showed a downward trend. However, with population growth and aging, the global burden of LBP continues to increase. Despite the global burden of LBP is high, current treatment methods have difficulty achieving complete cure.^[36]^ Therefore, early health education and risk factor control are key to preventing and reducing the burden of LBP. Countries with high SDI should strengthen health screening and education, while middle and low-income regions need to optimize primary healthcare resource allocation to improve efficiency. While we cannot change factors like genetics and aging, modifiable lifestyle factors such as smoking, obesity, and lack of physical exercise also impact LBP.^[22,27]^ Studies have shown that lifestyle interventions and physical exercise can improve chronic pain in adults and reduce disability associated with LBP.^[10,37]^ Prevention policies at the global, regional, and national levels should emphasize healthy lifestyles, such as exercise and balanced diets, to combat obesity and poor habits, ultimately improving quality of life and physical and mental health.^[38]^ Overall, LBP is a global health issue with significant regional disparities, with the burden particularly heavy for middle-aged and elderly women. When formulating health policies, decision makers should integrate various risk factors and formulate personalized prevention and treatment measures for LBP according to local conditions, so as to more effectively reduce the burden of LBP.

The global burden of low back pain exhibits significant geographical variation. However, these patterns must be interpreted through a critical lens of data heterogeneity. Elevated reported rates in high-SDI regions likely reflect superior healthcare infrastructure and integrated health information systems, which enhance case ascertainment.^[39,40]^ Conversely, in low- and middle-income countries with fragmented primary care, underreporting and diagnostic inconsistencies likely lead to an underestimation of the true burden. Furthermore, transnational differences in clinical coding practices and a general lack of granular,^[14]^ micro-level validation data mean that the observed disparities represent estimated burdens rather than a perfect reflection of reality. This potential bias underscores the imperative to strengthen global surveillance systems for musculoskeletal disorders.

Several limitations should be considered when interpreting the findings of this study. First, the findings are based on modeled data from the GBD study. Although complex statistical methods were applied to ensure consistency and address data gaps, these estimates remain subject to uncertainty and should not be considered exact measurements. Consequently, our results represent the best available modeled estimates rather than precise measurements. Second, while the frontier analysis is useful for identifying outliers, its fundamental assumption, a deterministic association between the SDI and the theoretically optimal disease burden, may be an oversimplification, as it fails to capture the influence of other complex factors like specific health policies or cultural contexts. Finally, the exclusion of risk factor attribution analysis limits our ability to interpret the underlying associated factors of the observed trends and disparities. Despite these limitations, this study still provides an up-to-date and comprehensive perspective on the management of LBP disease.

## 5. Conclusion

Globally, LBP remains a serious public health problem, with great variation across countries and regions. Although the ASPR, ASIR, and ASDR have declined over the past 3 decades, the burden remains high, especially among middle-aged and older women. Population growth and aging are the main demographic factors associated with the LBP burden. Understanding the regional distribution characteristics and risk factors of LBP helps formulate effective public health policies to reduce future disease burden. It is recommended that policymakers prioritize addressing high disease burden in areas with high SDI, while optimizing resource allocation and strengthening prevention systems in regions of rapid population growth in Asia.

## Acknowledgments

The authors wish to acknowledge the invaluable work of the Global Burden of Disease Study 2021 collaborators.

## Author contributions

**Conceptualization:** Jin Fu, Tingxiao Zhao, Lijiang Tao.

**Data curation:** Yuxin Ye, Yanlei Li, Yuan Zhang.

**Investigation:** Yuan Zhang, Tingxiao Zhao.

**Supervision:** Jin Fu, Yanlei Li, Shanggao Xie, Jun Zhang, Lijiang Tao.

**Validation:** Shanggao Xie, Jun Zhang.

**Visualization:** Yanlei Li, Tingxiao Zhao.

**Writing – original draft:** Yuxin Ye, Yanlei Li.

**Writing – review & editing:** Jin Fu, Lijiang Tao.






























