# Supplementary material for: Epidemiological trends and geographic disparities in low back pain burden based on the 2021 GBD study: A cross-sectional analysis
Source: Medicine (Baltimore). 2026 Jun 12;105(24):e49201. doi: 10.1097/MD.0000000000049201 (PMC13268564; doi:10.1097/MD.0000000000049201)

Figure S1. Global trends in LBP burden, 1990-2021. Shows the divergence between declining age-standardized rates (lines) and rising absolute case numbers (bars), highlighting the impact of demographic growth.

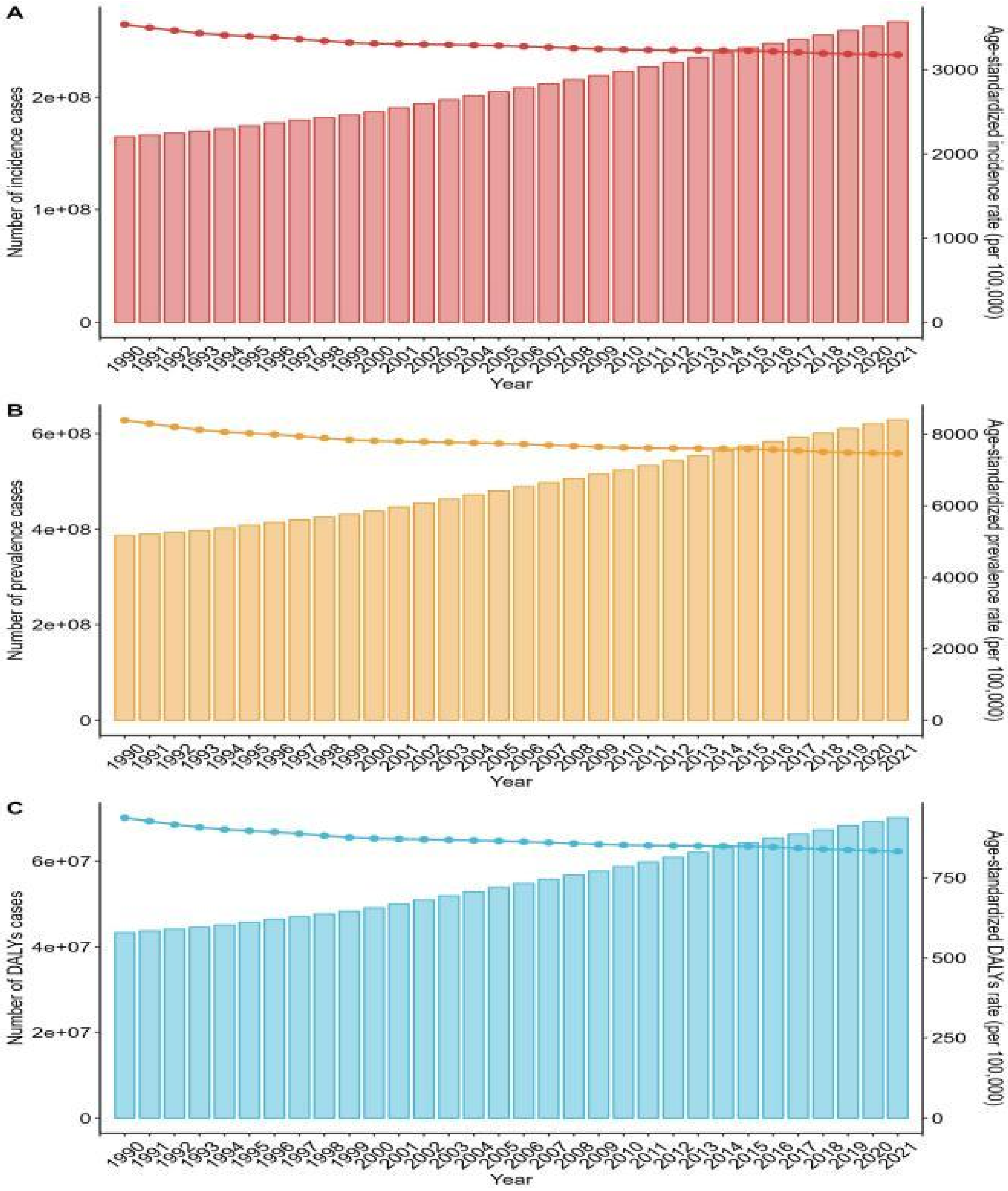

Supplement: Supplementary file 1 [file medi-105-e49201-s001.pdf]
