# Supplementary material for: Epidemiological trends and geographic disparities in low back pain burden based on the 2021 GBD study: A cross-sectional analysis
Source: Medicine (Baltimore). 2026 Jun 12;105(24):e49201. doi: 10.1097/MD.0000000000049201 (PMC13268564; doi:10.1097/MD.0000000000049201)
Supplement: Supplementary file 2 [file medi-105-e49201-s002.docx]

Table S1. The prevalent cases and ASR for LBP in all GBD regions between 1990 and 2021, and its temporal trends.

**1990 2021**

**Location**

**ASR per 100 000 (95%**

**ASR per 100 000**

**(95% UI)**

**Cases (95% UI)**

**Cases (95% UI)**

**UI)**

**EAPC (95% CI) 1990-2021**

**Global**

386731361

(341581662-434164620)

8391.58

(7381.14-9367.39)

628838475

(551834407-700881341)

7463.13

(6575.68-8321.8)

-0.32

(-0.35 to -0.28)

**GBD region**

| Advanced Health System | 161587419  (143872850-179554921) | 10863.99  (9687.51-12072.79) | 208649932  (186755701-228968090) | 10190.72  (9177.58-11238.29) | -0.15  (-0.17 to -0.13) |
| --- | --- | --- | --- | --- | --- |
| Africa | 31794322  (28109148-36039492) | 7721.34  (6773.4-8655.07) | 72985789  (64492809-82599513) | 7493.02  (6570.61-8398.82) | -0.1  (-0.13 to -0.08) |
| African Region | 23963570  (21143246-27116462) | 7477.4  (6540.22-8367.83) | 56181344  (49588465-63668506) | 7241.28  (6331.68-8109.11) | -0.11  (-0.13 to -0.09) |
| America | 63700953  (56770907-71383796) | 9553.32  (8495.17-10683.41) | 106545480  (96623196-116294833) | 9022.45  (8203.14-9882.87) | -0.12  (-0.15 to -0.08) |
| Andean Latin America | 1640636  (1443039-1863568) | 5748.76  (5034.13-6474.59) | 3735038 (3274646-4209857) | 5769.81  (5050.62-6502.89) | 0.03 (0.01-0.06) |
| Asia | 190990220  (167792495-216241942) | 7398.33  (6463.31-8289.06) | 329178566  (286241764-369686846) | 6510.48  (5690.1-7286.61) | -0.32  (-0.38 to -0.26) |
| Australasia | 2728928  (2438871-3043336) | 12314.65  (11042.6-13718.62) | 4389558 (3843185-4905996) | 11327.03  (9980.82-12746.38) | -0.19  (-0.22 to -0.17) |
| Basic Health System | 134359264  (117899517-152566043) | 7067.55  (6166.52-7939.58) | 232634652  (202084829-261302536) | 6464.37  (5673.18-7245.59) | -0.17  (-0.23 to -0.11) |
| Caribbean | 1873836  (1653012-2118352) | 6092.39  (5347.57-6854.23) | 3086535 (2735228-3457070) | 6006.95  (5348.69-6726.06) | -0.01 (-0.02-0) |
| Central Africa | 3244036  (2853453-3668766) | 7866.87  (6892.8-8791.07) | 8115406 (7151843-9250676) | 7534.85  (6585.24-8467.08) | -0.16  (-0.19 to -0.13) |
| Central Asia | 5123333  (4534991-5736765) | 9298.22  (8196.51-10320.92) | 8445849 (7373427-9503998) | 9188.48  (8032.34-10254.54) | -0.03  (-0.04 to -0.02) |
| Central Europe | 18424682  (16237814-20534270) | 13198.96  (11644.23-14728.93) | 20610737  (18182273-22911381) | 12831.04  (11293.99-14267.18) | -0.1  (-0.11 to -0.09) |
| Central Latin America | 9231074  (8138286-10600871) | 7394.45  (6490.72-8348.32) | 19673938  (17310811-22160905) | 7487.05  (6597.82-8413.59) | 0.04 (0-0.09) |
| Central Sub-Saharan Africa | 2666476  (2333799-3016266) | 7872.21  (6875.11-8803.69) | 6759109 (5963457-7709322) | 7619.29  (6670.28-8565.24) | -0.13  (-0.16 to -0.1) |
| Commonwealth High Income | 12632283  (11230778-14079939) | 9914.64  (8810.21-11088.73) | 18297444  (16050159-20426417) | 9564.66  (8426.3-10760.55) | -0.01  (-0.04-0.02) |
| Commonwealth Low Income | 11609082  (10319350-13127147) | 8924.88  (7947.17-9883.2) | 25410812  (22489294-28611248) | 8388.7  (7337.89-9399.65) | -0.16  (-0.21 to -0.12) |
| Commonwealth Middle | 62825039 | 7338.12 | 124405128 | 6620.14 | -0.34 |
| Income | (55178761-71135452) | (6397.24-8233.46) | (108575539-140884468) | (5746.75-7439.83) | (-0.44 to -0.25) |
| East Asia | 70972106  (61584149-80733977) | 6652.23  (5797.61-7476.49) | 105135609  (91895083-118365184) | 5418.74  (4746.47-6045.66) | -0.47  (-0.57 to -0.36) |

| East Asia & Pacific - WB | 115497744  (100955497-130784569) | 7080.68  (6177.38-7936.43) | 177945794  (155614768-200070908) | 5991.79  (5249.41-6687.8) | -0.4  (-0.47 to -0.34) |
| --- | --- | --- | --- | --- | --- |
| Eastern Africa | 8433709  (7451567-9567319) | 8001.66  (7020.65-8975.88) | 19731989  (17408592-22410692) | 7693.42  (6725.97-8611.71) | -0.13  (-0.14 to -0.13) |
| Eastern Europe | 30221827  (26656521-33743437) | 11653.11  (10264.38-12960.92) | 31818730  (28084336-35390254) | 11189.88  (9858.28-12447.24) | -0.07  (-0.09 to -0.05) |
| Eastern Mediterranean Region | 22143770  (19572389-25287613) | 8354.76  (7332.19-9415.87) | 53737230  (47441432-61183512) | 8377.42  (7348.01-9434.18) | 0.04 (0.03-0.06) |
| Eastern Sub-Saharan Africa | 8853965  (7817977-10027008) | 7890 (6905.07-8826.62) | 20671419  (18268068-23441517) | 7607.2  (6640.3-8512.38) | -0.13  (-0.14 to -0.12) |
| Europe | 99541740  (88507765-110589667) | 10776.83  (9565.48-11965.24) | 119202203  (105126305-132503575) | 10304.71  (9092.62-11495.9) | -0.11  (-0.12 to -0.09) |
| Europe & Central Asia - WB | 102885807  (91478725-114350658) | 10695.97  (9490.79-11879.56) | 125165846  (110490151-139182766) | 10199.04  (8987.37-11380.92) | -0.12  (-0.13 to -0.11) |
| European Region | 103643837  (92155078-115194946) | 10695.86  (9490.87-11878.83) | 126563245  (111716208-140736362) | 10200.08  (8988.3-11382.99) | -0.12  (-0.13 to -0.11) |
| High-income Asia Pacific | 21730692  (19102521-24328946) | 11083.01  (9779.81-12436.64) | 27054791  (23812392-30032356) | 10041.05  (8876.21-11287.71) | -0.27  (-0.29 to -0.25) |
| High-income North America | 35237734  (31463631-39080410) | 11238.41  (10024.5-12532.94) | 49456310  (46021985-52598499) | 10475.84  (9747.66-11177.32) | -0.07  (-0.13 to -0.02) |
| Latin America & Caribbean - WB | 28693631  (25418761-32558595) | 8063.06 (7064-9076.4) | 57386526  (50426901-64495838) | 8115.61  (7148.12-9105.81) | 0.01  (-0.01-0.03) |
| Limited Health System | 83882220  (74000365-95037754) | 7604.46  (6650.65-8511.62) | 171410981  (150063104-194196806) | 6972  (6071.06-7833.45) | -0.28  (-0.35 to -0.21) |
| Middle East & North Africa - | 16795173 | 8987.69 | 38936787 | 8724.05 | -0.09 |
| WB | (14883212-19089755) | (7971.58-10067.73) | (34448590-44034049) | (7707.18-9793.2) | (-0.1 to -0.07) |
| Minimal Health System | 6438810  (5668082-7288428) | 7595.91  (6646.2-8504.53) | 15531672  (13746255-17745082) | 7489.73  (6543.45-8404.67) | -0.06  (-0.08 to -0.04) |
| North Africa and Middle East | 22399030  (19902203-25305533) | 8937.83  (7994.23-9965.56) | 50601960  (44810213-57246491) | 8713.52  (7713-9767.15) | -0.06  (-0.08 to -0.05) |
| North America | 35236213  (31462309-39078790) | 11237.71  (10023.9-12532.14) | 49455657  (46021310-52597992) | 10475.32  (9747.18-11176.76) | -0.07  (-0.13 to -0.02) |
| Northern Africa | 7907472  (7022562-8983396) | 8699.29  (7748.89-9741.39) | 16721513  (14821607-18959626) | 8723.77  (7721.37-9800.62) | 0.01  (-0.03-0.06) |
| Oceania | 282858 (248890-321690) | 6380.65  (5600.75-7119.26) | 674940 (591101-768285) | 6322.16  (5498.31-7093.65) | -0.01  (-0.02-0.01) |
| Region of the Americas | 63700953  (56770907-71383796) | 9553.32  (8495.17-10683.41) | 106545480  (96623196-116294833) | 9022.45  (8203.14-9882.87) | -0.12  (-0.15 to -0.08) |
| South-East Asia Region | 71097873  (62820258-80403841) | 7331.06  (6421.84-8184.63) | 134191910  (116857132-151590079) | 6585.27  (5743.76-7386.4) | -0.34  (-0.42 to -0.27) |
| South Asia | 61510958  (54295998-69663661) | 7735.78  (6764.27-8668.52) | 119421340  (104121215-135119660) | 6919.73  (6023.41-7778.72) | -0.36  (-0.45 to -0.26) |
| South Asia - WB | 62969707  (55583123-71308543) | 7703.98  (6739.97-8629.54) | 122615543  (106947040-138681886) | 6922.42  (6027.35-7781.14) | -0.34  (-0.44 to -0.25) |

| Southeast Asia | 20564245  (18259667-23269347) | 5936.27  (5227.83-6631.53) | 42068815  (36528833-47513123) | 5859.18  (5118.41-6584.8) | -0.02  (-0.03 to -0.01) |
| --- | --- | --- | --- | --- | --- |
| Southern Africa | 4368590  (3863523-4924590) | 7167.84  (6270.55-7994.18) | 9319015  (8211437-10554981) | 7011.82  (6119.59-7857.56) | -0.06  (-0.07 to -0.05) |
| Southern Latin America | 4668528  (4103323-5247668) | 9770.4  (8585.81-10945.33) | 7546942 (6657533-8456318) | 9741.14  (8600.77-10945.72) | -0.02  (-0.06-0.02) |
| Southern Sub-Saharan Africa | 2452713  (2151707-2750769) | 6840.43  (5957.72-7647.28) | 4577210 (3987735-5172963) | 6510.35  (5661.38-7303.04) | -0.13  (-0.14 to -0.11) |
| Sub-Saharan Africa - WB | 23953764  (21149097-27135306) | 7472.83  (6542.36-8364.65) | 56387752  (49770091-63976603) | 7255.15  (6343.93-8124.49) | -0.1  (-0.12 to -0.08) |
| Tropical Latin America | 11347302  (10034300-12883622) | 9009.3  (7876.51-10155.83) | 23454673  (20450315-26355944) | 9303.75  (8187.67-10456.01) | 0.07 (0.04-0.1) |
| Western Africa | 7840516  (6924306-8872881) | 7034.18  (6153.64-7870.92) | 19097867  (16844120-21641607) | 6871.89  (6000.66-7706.3) | -0.09  (-0.12 to -0.05) |
| Western Europe | 46066166  (41107299-50853208) | 9854.31  (8811-10969.29) | 58122167  (51179306-64598752) | 9533.04  (8439.69-10690.62) | -0.07  (-0.08 to -0.06) |
| Western Pacific Region | 100142118  (87229710-113634263) | 7263.92  (6326.32-8151.35) | 147823176  (129241795-166462815) | 5984.04  (5235.61-6694.87) | -0.48  (-0.55 to -0.4) |
| Western Sub-Saharan Africa | 8734271  (7720212-9871622) | 7094.79  (6213.33-7933.42) | 21532804  (18994996-24407091) | 6911.71  (6038.99-7750.39) | -0.1  (-0.13 to -0.06) |
| World Bank High Income | 125532112  (112161648-139159993) | 10714.37  (9587.06-11932.17) | 167218766  (150398904-182875630) | 10062.55  (9108.38-11069.73) | -0.14  (-0.17 to -0.12) |
| World Bank Low Income | 15418667  (13602546-17424588) | 7812.43  (6838.2-8745.33) | 35116029  (31076848-39920684) | 7578.02  (6631.08-8484.4) | -0.12  (-0.13 to -0.1) |
| World Bank Lower Middle Income | 112620455  (99639813-127326629) | 7789.22  (6841.57-8705.13) | 219993032  (192581528-248393128) | 7078.6 (6180.2-7947) | -0.3  (-0.35 to -0.25) |
| World Bank Upper Middle | 132695270 | 7624.56 | 205897749 | 6688.45 | -0.31 |
| Income | (115964190-150198958) | (6660.57-8536.59) | (179386540-231431902) | (5869.48-7474.16) | (-0.38 to -0.24) |

ASR, age-standardized rate; LBP, low back pain; UI, uncertainty interval, CI, confdence interval; EAPC, estimated annual percentage change; DALYs, disability-adjusted life years.
