# Supplementary material for: Epidemiological trends and geographic disparities in low back pain burden based on the 2021 GBD study: A cross-sectional analysis
Source: Medicine (Baltimore). 2026 Jun 12;105(24):e49201. doi: 10.1097/MD.0000000000049201 (PMC13268564; doi:10.1097/MD.0000000000049201)
Supplement: Supplementary file 3 [file medi-105-e49201-s003.docx]

Table S6. The prevalent cases and ASR for LBP in 204 countries and territories between 1990 and 2021, and its temporal trends.

**Location**

**1990 2021**

| **Cases (95% UI) ASR per 100 000 (95% UI)** | **Cases (95% UI) ASR per 100 000 (95% UI)** |
| --- | --- |

**EAPC (95% CI) 1990-2021**

**Global**

386731361

(341581662-434164620)

8391.58

(7381.14-9367.39)

628838475

(551834407-700881341)

7463.13 (6575.68-8321.8)

-0.32

(-0.35 to -0.28)

**Country**

| Afghanistan | 652566 (573531-740069) | 8528.2 (7566.54-9657.6) | 1740282 (1523444-2003266) | 8582.35  (7532.18-9629.43) | 0.01 (0-0.03) |
| --- | --- | --- | --- | --- | --- |
| Albania | 351911 (311471-397083) | 12887.32  (11359.85-14502.21) | 447074 (393034-499676) | 13011.72  (11468.98-14627.03) | 0.02 (0.01-0.03) |
| Algeria | 1551910 (1359947-1762684) | 8441.78  (7439.59-9498.71) | 3521862 (3093604-3987279) | 8357.05  (7368.18-9404.42) | -0.06  (-0.07 to -0.04) |
| American Samoa | 2214 (1934-2517) | 6558.01  (5732.13-7411.07) | 3244 (2817-3653) | 6441.42  (5610.92-7245.08) | -0.04  (-0.05 to -0.02) |
| Andorra | 5784 (5137-6476) | 9489.83  (8362.35-10635.71) | 11037 (9628-12376) | 9207.44  (8094.02-10385.29) | -0.08  (-0.09 to -0.08) |
| Angola | 498461 (437390-568024) | 7945.74  (6975.49-8871.66) | 1503355 (1317401-1712709) | 7608.16  (6690.86-8544.03) | -0.17 (-0.2 to -0.13) |
| Antigua and Barbuda | 3339 (2929-3769) | 5988.53  (5211.17-6728.73) | 6248 (5425-7042) | 5965.27  (5229.16-6715.16) | -0.01 (-0.03-0) |
| Argentina | 3156731 (2771816-3553902) | 9785.39 (8599-10991.15) | 4934493 (4365734-5539534) | 9689.88  (8544.99-10902.93) | -0.03  (-0.07-0.01) |
| Armenia | 304742 (269172-344185) | 9902.49  (8783.45-11065.67) | 371065 (326269-413994) | 9917.94  (8766.38-10995.72) | 0.04 (0.02-0.05) |
| Australia | 2261634 (2014473-2520895) | 12243.28  (10979.33-13626.86) | 3632185 (3181104-4053379) | 11215.08  (9851.98-12602.58) | -0.21  (-0.24 to -0.17) |
| Austria | 837156 (735669-934509) | 8714.28  (7701.71-9733.63) | 1020074 (887488-1148851) | 8101.71  (7040.01-9159.66) | -0.07  (-0.12 to -0.01) |
| Azerbaijan | 547059 (483342-614795) | 9138.99  (8057.59-10181.41) | 1042998 (905544-1184527) | 9256.37  (8164.23-10352.97) | 0.08 (0.07-0.1) |
| Bahamas | 12950 (11367-14781) | 6012.99  (5244.99-6767.72) | 25754 (22331-29490) | 5988.04  (5211.44-6825.77) | -0.03  (-0.04 to -0.02) |
| Bahrain | 32745 (28293-38063) | 8169.9 (7170.14-9179.97) | 123876 (108170-141440) | 8022.63  (7091.52-9041.09) | -0.07  (-0.08 to -0.07) |
| Bangladesh | 6854026 (6137690-7734259) | 9763.14  (8693.55-10904.57) | 14161375  (12413606-15945019) | 9019.91  (7907.53-10138.84) | -0.2 (-0.26 to -0.13) |
| Barbados | 16240 (14248-18116) | 6102.45  (5318.41-6816.81) | 24130 (20842-27264) | 6041.64 (5263.3-6809.26) | -0.03  (-0.04 to -0.02) |
| Belarus | 1301307 (1148453-1454690) | 10937.04  (9648.92-12177.27) | 1404947 (1227482-1580532) | 10896.87  (9603.3-12215.07) | -0.01 (-0.02-0) |
| Belgium | 1207980 (1066272-1348533) | 9806.2  (8629.27-11017.33) | 1487462 (1312589-1651605) | 9579.03  (8459.85-10671.4) | -0.07  (-0.09 to -0.06) |

Belize 7902 (6952-8976) 6116.95 (5364.6-6865.19) 23990 (21031-27353) 6147.46 0.04 (0.03-0.05)

|  |  |  |  | (5360.62-6927.89) |  |
| --- | --- | --- | --- | --- | --- |
| Benin | 207166 (182164-235096) | 7310.88  (6370.53-8242.36) | 580741 (507806-659679) | 6990.71  (6069.07-7901.72) | -0.16  (-0.19 to -0.12) |
| Bermuda | 4073 (3561-4632) | 6222.44 (5460.8-7014.99) | 5731 (4987-6456) | 6140.18  (5404.86-6960.59) | -0.05  (-0.05 to -0.04) |
| Bhutan | 35580 (31344-40447) | 8863.25  (7728.54-9931.72) | 62894 (55240-71369) | 8675.14  (7620.28-9800.51) | -0.05  (-0.06 to -0.04) |
| Bolivia (Plurinational State of) | 278398 (244451-318991) | 6088.81  (5300.59-6902.29) | 670540 (588104-765274) | 6154.29  (5400.48-6941.47) | 0.01 (-0.03-0.05) |
| Bosnia and Herzegovina | 545205 (478419-612475) | 11903.71  (10516.19-13319.52) | 564674 (492508-634599) | 12273.98  (10776.12-13835.4) | 0.11 (0.09-0.12) |
| Botswana | 52487 (45614-59421) | 6526.42  (5646.54-7296.48) | 130644 (113278-148456) | 6505.43 (5682.32-7283.7) | 0.01 (-0.01-0.03) |
| Brazil | 11120243 (9831515-12631211) | 9047.71  (7909.56-10204.39) | 22920640  (19975535-25768038) | 9346.43  (8224.78-10501.25) | 0.07 (0.04-0.1) |
| Brunei Darussalam | 18415 (15997-21279) | 9002.97  (7934.58-10152.66) | 40769 (35695-46673) | 8622.35  (7618.23-9688.61) | -0.13  (-0.15 to -0.11) |
| Bulgaria | 1339666 (1172733-1513461) | 12612.96  (11087.34-14181.03) | 1219153 (1065091-1354885) | 12359.21  (10924.95-13774.09) | -0.05  (-0.06 to -0.05) |
| Burkina Faso | 416881 (368880-467848) | 7221.03  (6393.66-8021.51) | 992730 (877101-1123849) | 7008.71 (6163.73-7915.1) | -0.15  (-0.18 to -0.12) |
| Burundi | 267294 (235475-303877) | 8001.53  (6971.74-8977.87) | 614908 (537547-693814) | 7543.27 (6529.8-8441.58) | -0.23  (-0.24 to -0.21) |
| Cabo Verde | 16720 (14685-18776) | 6878.08  (6008.05-7748.46) | 34286 (29948-38719) | 6442.85  (5589.17-7204.82) | -0.24 (-0.28 to -0.2) |
| Cambodia | 435435 (383306-499868) | 6710.24 (5891.86-7576.9) | 934987 (813187-1057303) | 6196.23  (5436.47-6928.21) | -0.28  (-0.29 to -0.26) |
| Cameroon | 491364 (435576-554923) | 7598.98 (6660.2-8497.8) | 1486905 (1303167-1703216) | 7204.39  (6315.09-8106.24) | -0.2 (-0.23 to -0.16) |
| Canada | 2946272 (2599728-3314606) | 9620.36  (8438.35-10811.64) | 4333536 (3799309-4839311) | 8808.1 (7775.51-9920.89) | -0.2 (-0.23 to -0.17) |
| Central African Republic | 134200 (117714-153087) | 7801.09  (6866.76-8856.97) | 278572 (245144-316841) | 7699.72  (6727.79-8620.19) | -0.06  (-0.08 to -0.05) |
| Chad | 312464 (277518-349402) | 8362.22  (7404.02-9292.25) | 747933 (656127-849862) | 7664.38 (6654.57-8584) | -0.25  (-0.34 to -0.17) |
| Chile | 1202081 (1064111-1354424) | 9900.7 (8710.1-11058.93) | 2199064 (1931395-2475517) | 9831.34  (8668.68-11038.74) | -0.04  (-0.08 to -0.01) |
| China | 68281006  (59158022-77853897) | 6635.49 (5770.68-7472.8) | 100093746  (87128173-113014316) | 5342.1 (4660.41-5976.28) | -0.5 (-0.6 to -0.39) |
| Colombia | 2116129 (1867094-2422819) | 8086.05  (7102.97-9134.32) | 4387068 (3898841-4869218) | 8144.84  (7261.08-9049.88) | 0.05 (0.04-0.07) |
| Comoros | 20201 (17919-22589) | 7097.19 (6273.7-7856.99) | 44293 (38868-50176) | 7151.55  (6276.78-8044.87) | -0.04  (-0.07 to -0.02) |

Congo 112186 (98424-127076) 7293.81 297725 (259637-340600) 7247.8 (6297.9-8180.71) -0.05

|  |  | (6388.31-8180.12) |  |  | (-0.07 to -0.03) |
| --- | --- | --- | --- | --- | --- |
| Cook Islands | 985 (864-1121) | 6426.89  (5615.35-7278.28) | 1444 (1255-1612) | 6590.4 (5726.9-7430.14) | 0.12 (0.1-0.13) |
| Costa Rica | 188582 (166468-215485) | 7723.52 (6803.2-8708.26) | 399436 (351219-450635) | 7530.44  (6625.87-8487.53) | -0.07  (-0.08 to -0.07) |
| Croatia | 692671 (621881-765892) | 12202.65  (10932.22-13429.12) | 727775 (635603-811834) | 11995.24  (10504.44-13505.46) | -0.02  (-0.08-0.04) |
| Cuba | 673707 (590839-762972) | 6137.51  (5384.88-6935.88) | 876685 (791222-962271) | 5749.29 (5218.3-6303.35) | -0.09  (-0.12 to -0.05) |
| Cyprus | 78587 (68835-88477) | 9619.52  (8470.13-10885.6) | 163840 (143959-183692) | 9506.42  (8351.05-10631.82) | -0.06  (-0.08 to -0.05) |
| Czechia | 1662807 (1477052-1841916) | 13734.57  (12198.17-15220.97) | 1987875 (1746840-2216854) | 13298.59  (11720.8-14874.21) | -0.1 (-0.11 to -0.09) |
| Cote d’Ivoire | 531847 (470449-603590) | 7435.46  (6553.37-8289.53) | 1335591 (1174743-1534501) | 7168.93  (6236.54-8093.03) | -0.11  (-0.15 to -0.06) |
| Democratic People's Republic of Korea | 1360858 (1180789-1533062) | 7241.56  (6317.37-8075.41) | 2177911 (1892033-2458081) | 6808.29 (5925.2-7643.56) | -0.23  (-0.25 to -0.21) |
| Democratic Republic of the Congo | 1850318 (1616716-2097537) | 7919.44  (6907.03-8886.26) | 4505799 (3979196-5151954) | 7660.49  (6703.11-8614.04) | -0.13 (-0.16 to -0.1) |
| Denmark | 833644 (766445-901731) | 12984.95  (11937-14072.76) | 903094 (779843-1027887) | 11336.5  (9875.82-12888.7) | -0.71  (-0.84 to -0.58) |
| Djibouti | 17826 (15662-20600) | 7133.96 (6249.6-8068.29) | 66466 (58544-75945) | 6730.18  (5934.43-7561.62) | -0.22 (-0.23 to -0.2) |
| Dominica | 3948 (3469-4451) | 6297.07  (5489.81-7084.55) | 4713 (4105-5325) | 6071.32  (5304.36-6847.18) | -0.14  (-0.16 to -0.13) |
| Dominican Republic | 323940 (286922-368314) | 5978.75  (5255.55-6728.62) | 673428 (590865-762543) | 6179.72  (5426.01-6949.64) | 0.09 (0.07-0.12) |
| Ecuador | 434318 (382309-490236) | 5790.05  (5129.95-6475.14) | 960162 (857114-1075027) | 5455.79  (4865.81-6114.62) | -0.21 (-0.32 to -0.1) |
| Egypt | 3516624 (3113328-4021667) | 8434.65  (7419.97-9530.32) | 7705843 (6704374-8797681) | 8656.87 (7588.07-9775.7) | 0.1 (0.06-0.13) |
| El Salvador | 302072 (268185-342736) | 7541.95  (6633.45-8511.71) | 490166 (430668-553334) | 7735.76  (6786.93-8708.49) | 0.13 (0.12-0.15) |
| Equatorial Guinea | 20876 (18373-23592) | 7793.62 (6848.44-8731.9) | 71402 (62770-82269) | 7380.07 (6446.74-8329.6) | -0.21  (-0.23 to -0.19) |
| Eritrea | 130156 (114425-148942) | 6748.42 (5894.49-7515.1) | 304239 (266564-347828) | 6809.63 (5873.71-7649.5) | 0.06 (0.05-0.07) |
| Estonia | 200056 (179016-222516) | 10822.48  (9692.06-12046.2) | 205001 (180338-228597) | 10696.64  (9433.06-11842.71) | -0.01  (-0.03-0.02) |
| Eswatini | 26457 (23377-29969) | 5909.33 (5192.3-6559.76) | 47873 (41792-54339) | 5856.36  (5131.74-6566.97) | -0.08  (-0.11 to -0.04) |
| Ethiopia | 2374630 (2090462-2682737) | 8019.11  (7002.79-8973.95) | 5192958 (4573144-5923660) | 7402.48  (6450.47-8289.55) | -0.26  (-0.28 to -0.25) |
| Fiji | 35678 (31182-40847) | 6481.53  (5653.02-7300.53) | 54726 (47317-61864) | 6258 (5475.98-7040.28) | -0.11 (-0.11 to -0.1) |

| Finland | 535334 (476188-591803) | 8759.22 (7848.09-9688.3) | 663630 (580817-736549) | 8370.21 (7372.16-9330.6) | -0.1 (-0.12 to -0.09) |
| --- | --- | --- | --- | --- | --- |
| France | 6508552 (5785610-7201694) | 9575.33  (8489.75-10684.55) | 8606897 (7537365-9566141) | 9567.67  (8423.92-10697.51) | -0.02 (-0.04-0) |
| Gabon | 50435 (44220-56756) | 7199.97  (6281.31-8050.82) | 102256 (89983-115655) | 7175.3 (6302.67-8002.68) | -0.01 (-0.01-0) |
| Gambia | 38541 (33628-43878) | 6702.43  (5827.03-7547.23) | 101319 (89135-115870) | 6514.16  (5668.02-7331.75) | -0.1 (-0.14 to -0.06) |
| Georgia | 516795 (450504-577963) | 8662.53 (7577.4-9711.65) | 405869 (355697-452921) | 8546.13  (7533.18-9556.41) | -0.13  (-0.18 to -0.09) |
| Germany | 11840089  (10538749-13311846) | 11696.68  (10393.2-13148.01) | 13525638  (11953401-15074153) | 11089.56  (9825.79-12456.38) | -0.1 (-0.13 to -0.08) |
| Ghana | 620325 (553349-700336) | 6595.01  (5865.42-7331.65) | 1557171 (1370088-1750809) | 6224.21  (5503.97-6895.19) | -0.21  (-0.24 to -0.18) |
| Greece | 1143751 (1010403-1274088) | 9083.96  (8045.18-10171.58) | 1337382 (1163621-1490489) | 9017.14  (7847.22-10168.75) | -0.06  (-0.09 to -0.03) |
| Greenland | 4786 (4180-5498) | 9115.31  (8116.14-10280.63) | 5609 (4873-6399) | 8629.82 (7616.16-9745.8) | -0.12  (-0.15 to -0.09) |
| Grenada | 4408 (3890-4970) | 6123.86  (5366.59-6880.97) | 6882 (6005-7808) | 6042.09 (5319.92-6822.4) | -0.03  (-0.04 to -0.01) |
| Guam | 7064 (6170-7999) | 6365.41  (5533.85-7121.09) | 11788 (10218-13211) | 6314.48  (5507.28-7059.52) | -0.01 (-0.02-0) |
| Guatemala | 492361 (436794-555102) | 8933.72  (7926.25-9972.46) | 1193584 (1044791-1362887) | 8525.94  (7442.89-9615.06) | -0.09  (-0.13 to -0.04) |
| Guinea | 295065 (258790-334095) | 7233.55  (6331.89-8179.55) | 607335 (538746-691542) | 7145.37  (6234.99-8045.14) | -0.09  (-0.13 to -0.04) |
| Guinea-Bissau | 41317 (36194-47240) | 6916.19  (5955.94-7794.11) | 86687 (76071-99775) | 6791.53  (5927.99-7699.64) | -0.09  (-0.12 to -0.05) |
| Guyana | 35252 (30895-40281) | 6069.4 (5322.36-6855.35) | 44261 (38612-50188) | 6025.23  (5270.01-6796.04) | -0.03  (-0.03 to -0.03) |
| Haiti | 279072 (244551-318867) | 6087.77  (5333.23-6898.96) | 640533 (560029-734333) | 6036.33  (5283.41-6816.84) | -0.05  (-0.07 to -0.03) |
| Honduras | 239411 (211012-273404) | 7591.31 (6621.8-8564.09) | 674277 (595436-775784) | 7737.63  (6772.48-8785.18) | 0.08 (0.07-0.09) |
| Hungary | 1790446 (1593259-1997888) | 14289.59  (12701.87-15966.22) | 1915401 (1702105-2131974) | 14024.64  (12361.13-15712.34) | -0.06  (-0.06 to -0.05) |
| Iceland | 27508 (24146-30693) | 10310.47  (9069.43-11519.79) | 42195 (37054-47174) | 9664.49  (8562.24-10854.22) | -0.24  (-0.26 to -0.23) |
| India | 47842656  (42039472-54147203) | 7525.7 (6558.53-8440.89) | 88378724  (76690994-99816577) | 6495.18  (5653.16-7292.19) | -0.5 (-0.62 to -0.38) |
| Indonesia | 8769934 (7738469-9950876) | 6381.23  (5566.75-7157.83) | 17495212  (15170534-19717471) | 6207.79  (5421.45-6957.03) | -0.04  (-0.06 to -0.02) |
| Iran (Islamic Republic of) | 4033747 (3564298-4581475) | 10164.87  (8997.03-11356.67) | 8280715 (7280760-9354790) | 9275.86  (8213.42-10410.11) | -0.24  (-0.29 to -0.19) |

Iraq 1074684 (947243-1217765) 8488.92 2936152 (2586751-3351786) 8343.82 (7322.81-9401.1) -0.05

|  |  | (7517.61-9520.86) |  |  | (-0.06 to -0.05) |
| --- | --- | --- | --- | --- | --- |
| Ireland | 372043 (325548-418467) | 9902.79  (8631.53-11086.92) | 605072 (531564-672017) | 9843.33  (8706.38-10982.67) | -0.1 (-0.13 to -0.07) |
| Israel | 487078 (433787-541590) | 10177.91  (9049.26-11294.94) | 1007587 (884790-1126942) | 9756.09  (8616.81-10954.91) | -0.12  (-0.15 to -0.09) |
| Italy | 6968334 (6085183-7787818) | 9732.76  (8519.85-10945.8) | 8595373 (7523229-9562297) | 9632.31  (8440.97-10814.43) | -0.1 (-0.12 to -0.08) |
| Jamaica | 123457 (108661-140064) | 6238.69 (5417.68-7050.3) | 191529 (166504-217014) | 6231.81  (5416.02-7066.83) | -0.03  (-0.05 to -0.01) |
| Japan | 17401998  (15257688-19461937) | 11575.73  (10253.68-12977.41) | 20008724  (17610370-22194703) | 10626.15  (9373.76-11935.56) | -0.21  (-0.24 to -0.18) |
| Jordan | 215134 (189300-247098) | 8573.8 (7589.52-9636.99) | 932614 (820414-1063564) | 8451.99  (7479.98-9519.67) | -0.04  (-0.05 to -0.04) |
| Kazakhstan | 1364249 (1207479-1515371) | 9518.46  (8412.29-10513.16) | 1808242 (1574058-2036469) | 9515.34  (8317.49-10667.14) | -0.03  (-0.06 to -0.01) |
| Kenya | 1077197 (954586-1227217) | 8315.01  (7250.93-9306.07) | 2842418 (2517504-3226416) | 8096.48 (7070.81-9068.9) | -0.09  (-0.12 to -0.07) |
| Kiribati | 3377 (2965-3842) | 6529.64  (5704.51-7316.21) | 6494 (5648-7358) | 6727.2 (5844.13-7576.32) | 0.18 (0.15-0.21) |
| Kuwait | 110884 (96638-129245) | 8034.3 (7081.74-9072.53) | 413683 (357544-477404) | 8396.07  (7427.68-9458.53) | 0.15 (0.13-0.17) |
| Kyrgyzstan | 331545 (292351-372755) | 9548.6  (8424.91-10675.16) | 543012 (473906-613015) | 9210.53  (8054.39-10334.84) | -0.12  (-0.13 to -0.11) |
| Lao People's Democratic Republic | 168945 (149433-191047) | 6103.1 (5379.69-6829.61) | 359138 (315417-411195) | 5795.91 (5076.15-6553.8) | -0.18  (-0.21 to -0.16) |
| Latvia | 340664 (302350-382212) | 10704.79  (9492.11-11924.01) | 295163 (259792-330798) | 10520.37  (9253.12-11796.36) | -0.07  (-0.08 to -0.06) |
| Lebanon | 214032 (187916-241794) | 8313.42 (7311-9318.66) | 497058 (438187-558082) | 8319.97 (7301.53-9337.7) | 0.06 (0.04-0.09) |
| Lesotho | 69629 (60903-78743) | 6696.86  (5847.17-7520.92) | 90595 (79699-102961) | 6376.37  (5600.61-7152.14) | -0.21  (-0.23 to -0.18) |
| Liberia | 109426 (96293-123552) | 6969.34  (6096.22-7818.73) | 249479 (219063-286106) | 6756.55  (5859.08-7633.34) | -0.13  (-0.14 to -0.11) |
| Libya | 251646 (222272-288128) | 8370.25 (7372.86-9447.3) | 587793 (512910-668675) | 8369.97  (7322.04-9461.15) | -0.02  (-0.03 to -0.01) |
| Lithuania | 456295 (401665-508994) | 10943.73  (9668.74-12218.43) | 436060 (380514-487270) | 10696.23  (9362.77-12003.14) | -0.09  (-0.11 to -0.08) |
| Luxembourg | 45970 (40535-51328) | 9860.26  (8727.82-11035.08) | 79859 (70342-89230) | 9567.96  (8462.71-10727.77) | -0.12  (-0.13 to -0.11) |
| Madagascar | 593164 (522642-679083) | 8089.45  (7087.33-9073.24) | 1397380 (1225476-1597018) | 7522.46  (6554.68-8480.72) | -0.28 (-0.3 to -0.25) |
| Malawi | 448583 (398269-504115) | 7719.58  (6805.35-8557.95) | 925440 (814245-1060347) | 7601.69 (6638.82-8538.8) | -0.08 (-0.1 to -0.06) |
| Malaysia | 749702 (674461-831354) | 5646.69  (5101.43-6192.75) | 1754495 (1529030-1991575) | 5491.53  (4778.01-6190.28) | -0.17  (-0.22 to -0.12) |

| Maldives | 7156 (6272-8163) | 5298.27  (4627.27-5935.27) | 25850 (22502-29840) | 5020.83  (4401.32-5671.95) | -0.18  (-0.21 to -0.14) |
| --- | --- | --- | --- | --- | --- |
| Mali | 332933 (292968-374731) | 6120.96  (5353.83-6862.75) | 888570 (779419-1011155) | 6343.48  (5534.65-7141.25) | 0.07 (0.05-0.1) |
| Malta | 41275 (36298-46273) | 10132.62  (8933.5-11350.84) | 63750 (56479-70919) | 10100.38  (9017.14-11353.89) | -0.08  (-0.11 to -0.06) |
| Marshall Islands | 1637 (1439-1882) | 6269.96 (5443.88-7065.3) | 2905 (2518-3311) | 6060.97 (5286.29-6826.8) | -0.11  (-0.11 to -0.11) |
| Mauritania | 84741 (74901-95858) | 6319.43  (5577.33-7054.48) | 189942 (167032-215024) | 6325.49 (5520.8-7107.65) | -0.06  (-0.09 to -0.03) |
| Mauritius | 57327 (50742-64828) | 6126.63  (5432.82-6834.57) | 93863 (80189-105460) | 5756.61 (4990.9-6431.86) | -0.15  (-0.19 to -0.11) |
| Mexico | 4491909 (3941999-5143189) | 6978.51  (6104.13-7912.25) | 9705422 (8453488-11018157) | 7187.42 (6264.81-8143.3) | 0.08 (-0.03-0.19) |
| Micronesia (Federated States of) | 4419 (3875-5024) | 6587.21  (5734.99-7409.15) | 5999 (5220-6809) | 6605.14  (5803.66-7454.28) | 0.04 (0.02-0.06) |
| Monaco | 4282 (3770-4783) | 9552.29  (8410.41-10732.05) | 5313 (4656-5951) | 9217.81  (8125.59-10374.3) | -0.11 (-0.12 to -0.1) |
| Mongolia | 134932 (119826-152356) | 9313.85  (8194.4-10428.73) | 266853 (231712-303765) | 9044.92  (7952.4-10176.84) | -0.12  (-0.13 to -0.11) |
| Montenegro | 80700 (70934-90086) | 12536.2  (11020.31-13989.58) | 100518 (87811-113076) | 12684.44  (11184.73-14300) | 0.06 (0.05-0.07) |
| Morocco | 1936482 (1729102-2197226) | 9764.03  (8729.16-10953.93) | 3594308 (3177048-4050695) | 9590.36  (8505.95-10797.91) | -0.03  (-0.13-0.08) |
| Mozambique | 683904 (603454-775329) | 8091.27  (7123.37-9115.38) | 1473284 (1300260-1675989) | 8037.15  (7066.52-8990.92) | -0.01 (-0.02-0) |
| Myanmar | 1514648 (1335921-1728873) | 5018.8 (4397.18-5669.43) | 2740284 (2389488-3118188) | 5056.85  (4441.86-5710.22) | -0.02  (-0.06-0.01) |
| Namibia | 60061 (53423-67233) | 6745.7 (5950.83-7495.86) | 125760 (109623-142882) | 6738.73 (5872.6-7594.86) | 0.04 (0.02-0.06) |
| Nauru | 451 (395-514) | 6426.54  (5624.53-7245.08) | 551 (482-627) | 6670.83  (5802.28-7476.14) | 0.15 (0.12-0.17) |
| Nepal | 1544505 (1371676-1753622) | 11501.6  (10204.86-12970.5) | 2893257 (2548839-3275501) | 10402.94  (9164.82-11750.04) | -0.24  (-0.34 to -0.15) |
| Netherlands | 1496346 (1305899-1678983) | 8558.16  (7495.39-9558.79) | 1983906 (1720121-2224849) | 8313.56  (7276.84-9344.45) | -0.05  (-0.11-0.01) |
| New Zealand | 467295 (413196-519723) | 12687.16  (11163.69-14161.99) | 757373 (663718-849958) | 11896.74  (10529.49-13415.3) | -0.14  (-0.16 to -0.13) |
| Nicaragua | 199009 (175228-228181) | 7804.92 (6841.5-8839.85) | 476898 (418988-540128) | 7737.93  (6801.22-8740.97) | 0.01 (-0.01-0.02) |
| Niger | 311989 (272938-358535) | 6914.47  (6000.71-7800.19) | 961562 (844164-1095884) | 7106.03 (6251.49-7985.7) | 0.14 (0.1-0.18) |
| Nigeria | 4269265 (3750911-4831320) | 7124.31  (6215.63-7994.73) | 10200135 (8992602-11594712) | 7000.06  (6102.69-7867.01) | -0.06 (-0.1 to -0.02) |

Niue 137 (121-155) 6469.34 125 (108-141) 6384.67 (5536.41-7180.2) -0.03

|  |  | (5684.51-7322.08) |  |  | (-0.04 to -0.02) |
| --- | --- | --- | --- | --- | --- |
| North Macedonia | 234463 (204659-265083) | 11662.32  (10220.16-13109.09) | 331671 (287839-372757) | 11609.65  (10161.18-12973.66) | 0 (-0.01-0) |
| Northern Mariana Islands | 2307 (2010-2684) | 6369.64  (5618.26-7177.31) | 3463 (2965-3942) | 6330.05 (5497.2-7161.1) | -0.04  (-0.07 to -0.01) |
| Norway | 490688 (432271-544345) | 9424.38  (8281.53-10562.62) | 636231 (557163-709406) | 8871.11  (7777.91-9939.32) | -0.22 (-0.24 to -0.2) |
| Oman | 112281 (99214-128802) | 8103.46 (7137.19-9108.2) | 335904 (291421-388406) | 7948.79  (7000.98-8982.24) | -0.06  (-0.06 to -0.05) |
| Pakistan | 5234191 (4523822-6069187) | 7048.51  (6070.55-8089.97) | 13925090  (12007269-16056430) | 7787.21 (6686.41-8936.8) | 0.44 (0.36-0.51) |
| Palau | 813 (718-925) | 6379.05 (5601.5-7177.93) | 1415 (1220-1614) | 6222.34  (5421.67-6993.18) | -0.05  (-0.08 to -0.03) |
| Palestine | 116459 (103404-132736) | 8683.04  (7671.82-9744.06) | 341245 (302855-391250) | 8389.82 (7460.3-9482.58) | -0.1 (-0.11 to -0.09) |
| Panama | 147132 (129879-168269) | 7396.34  (6522.61-8335.63) | 328200 (287898-371245) | 7461.51 (6558-8435.5) | 0.03 (0.02-0.05) |
| Papua New Guinea | 173028 (152044-197274) | 6304.72  (5534.85-7045.39) | 484332 (423829-552993) | 6257.15  (5445.77-7041.12) | 0 (-0.02-0.02) |
| Paraguay | 227059 (202770-255183) | 7452.52  (6648.12-8298.92) | 534033 (467609-606411) | 7799.54 (6799.9-8833.81) | 0.13 (0.06-0.2) |
| Peru | 927920 (814632-1058117) | 5637.78 (4917.55-6369.6) | 2104337 (1846504-2388521) | 5805.87  (5078.43-6566.62) | 0.15 (0.12-0.18) |
| Philippines | 2718984 (2408919-3093527) | 6227.18 (5429-6988.53) | 6069056 (5306912-6865982) | 6075.61 (5291.1-6805.08) | -0.12 (-0.14 to -0.1) |
| Poland | 5467417 (4811083-6087896) | 13153.59  (11632.52-14646.5) | 6752690 (5945177-7494972) | 12733.25  (11216.09-14164.42) | -0.13  (-0.14 to -0.11) |
| Portugal | 1242693 (1089575-1386627) | 10418.52  (9160.58-11664.8) | 1594817 (1418508-1756789) | 10220.31  (9098.41-11365.85) | -0.13  (-0.16 to -0.09) |
| Puerto Rico | 218708 (192299-247276) | 6009.32  (5270.91-6807.95) | 283448 (246010-319443) | 6003.56 (5235.2-6781.99) | -0.02  (-0.04 to -0.01) |
| Qatar | 28895 (24918-33959) | 7887.69  (6928.13-8859.43) | 235940 (201753-278425) | 8067.42  (7119.23-9079.04) | 0.02 (0-0.04) |
| Republic of Korea | 4062541 (3563523-4649682) | 9718.86  (8541.53-10954.36) | 6450707 (5626961-7267718) | 9025.43  (7908.34-10208.85) | -0.22 (-0.24 to -0.2) |
| Republic of Moldova | 486239 (428380-548446) | 10859.89  (9582.49-12161.79) | 527810 (464804-593650) | 10662.81  (9388.29-11973.41) | -0.1 (-0.12 to -0.08) |
| Romania | 3551341 (3094005-3983858) | 13646.2  (11958.72-15325.18) | 3418295 (2996915-3814026) | 12906.32  (11314.5-14506.14) | -0.23  (-0.25 to -0.21) |
| Russian Federation | 19274148  (16967111-21537164) | 11311.87  (9963.45-12583.8) | 21356309  (18787451-23691942) | 10885.16  (9558.53-12109.4) | -0.03  (-0.06 to -0.01) |
| Rwanda | 354904 (313220-403935) | 8364.13  (7345.01-9381.28) | 765497 (674154-866796) | 8124.73  (7152.18-9035.89) | -0.13  (-0.15 to -0.11) |
| Saint Kitts and Nevis | 2237 (1973-2527) | 6091.19  (5333.83-6856.28) | 4168 (3603-4718) | 5938.88 (5223.94-6707.4) | -0.09  (-0.09 to -0.09) |

| Saint Lucia | 6891 (6043-7891) | 6427.93  (5607.09-7276.55) | 13425 (11666-15221) | 6128.82 (5342.8-6938.09) | -0.16  (-0.16 to -0.15) |
| --- | --- | --- | --- | --- | --- |
| Saint Vincent and the Grenadines | 5195 (4548-5918) | 6033.42  (5231.66-6848.04) | 7774 (6710-8772) | 5925.97 (5140.63-6690.9) | -0.05  (-0.05 to -0.05) |
| Samoa | 7807 (6857-8880) | 6891.46  (6010.82-7768.81) | 11141 (9680-12684) | 6521.23  (5693.61-7358.61) | -0.24 (-0.27 to -0.2) |
| San Marino | 2783 (2444-3093) | 9613.26  (8485.78-10755.06) | 4390 (3867-4875) | 9268.5  (8175.27-10378.97) | -0.11  (-0.12 to -0.11) |
| Sao Tome and Principe | 4898 (4320-5525) | 6237.93  (5461.23-6994.48) | 9905 (8693-11313) | 6060.07  (5301.89-6830.75) | -0.1 (-0.12 to -0.09) |
| Saudi Arabia | 900969 (793441-1026567) | 7998.85  (7035.49-8974.03) | 3046049 (2639437-3517216) | 8183.71 (7179.18-9185) | 0.1 (0.09-0.1) |
| Senegal | 299204 (264885-338146) | 6536.88  (5760.68-7332.21) | 694412 (604478-788850) | 6295.91  (5487.08-7128.35) | -0.19  (-0.23 to -0.16) |
| Serbia | 1388614 (1208087-1554390) | 12823.58  (11270.15-14290) | 1564729 (1377561-1739735) | 12848.39  (11287.1-14321.05) | 0 (0-0.01) |
| Seychelles | 3505 (3106-3942) | 5642.49  (4952.39-6347.44) | 6349 (5468-7200) | 5340.09  (4649.82-6024.65) | -0.19 (-0.2 to -0.18) |
| Sierra Leone | 199087 (173793-225503) | 7207.68  (6297.33-8120.06) | 402507 (353574-459066) | 6793.39  (5910.23-7674.55) | -0.2 (-0.22 to -0.17) |
| Singapore | 247738 (218203-280718) | 8070.33  (7079.64-9079.01) | 554591 (478477-633875) | 7432.27  (6469.72-8402.66) | -0.14  (-0.19 to -0.08) |
| Slovakia | 754732 (670829-836725) | 13277.43  (11835.64-14771.76) | 932278 (815318-1040375) | 12675.05  (11137.01-14064.97) | -0.13  (-0.15 to -0.12) |
| Slovenia | 270064 (238077-301285) | 11836.39  (10458.22-13166.22) | 348609 (303595-389183) | 11604.67  (10076.82-13015.74) | -0.08  (-0.09 to -0.06) |
| Solomon Islands | 12982 (11298-14899) | 6240.15 (5452.41-7036.5) | 33662 (29645-38463) | 6683.08  (5873.26-7521.36) | 0.3 (0.27-0.33) |
| Somalia | 332154 (291589-382071) | 7593.2 (6632.88-8508.98) | 896557 (791541-1023167) | 7707.47  (6766.72-8641.29) | 0.02 (0-0.03) |
| South Africa | 1828261 (1597558-2060239) | 6852.39 (5968.8-7689.17) | 3413489 (2969977-3842766) | 6348.57  (5524.91-7134.81) | -0.21  (-0.23 to -0.19) |
| South Sudan | 271315 (236963-308512) | 7407.44  (6482.06-8310.04) | 449966 (395095-513432) | 7362.27  (6461.62-8309.05) | -0.03  (-0.05 to -0.02) |
| Spain | 3778557 (3470512-4071798) | 8246.48  (7614.15-8925.29) | 4886811 (4194607-5507508) | 7499 (6554.63-8524.74) | -0.06  (-0.17-0.05) |
| Sri Lanka | 799026 (704572-897682) | 5671.96  (5024.33-6294.15) | 1428070 (1243386-1604885) | 5630.58  (4954.24-6315.15) | -0.03  (-0.05 to -0.01) |
| Sudan | 1209950 (1074866-1380961) | 8579.04  (7637.52-9679.92) | 2831712 (2489806-3243962) | 8519.24  (7467.08-9569.09) | -0.02  (-0.02 to -0.01) |
| Suriname | 19496 (17083-22019) | 6022.57  (5248.53-6758.34) | 38433 (33418-43260) | 6147.19  (5363.91-6925.22) | 0.09 (0.07-0.1) |
| Sweden | 673497 (606451-739171) | 5909.66  (5328.92-6483.76) | 1025162 (876881-1155495) | 7137.31 (6144.5-8163.41) | 0.6 (0.46-0.75) |

| Switzerland | 937038 (866555-1002711) | 11264.32  (10420.27-12070.23) | 1240148 (1092899-1377665) | 10111 (8915.72-11348.45) | -0.16  (-0.29 to -0.03) |
| --- | --- | --- | --- | --- | --- |
| Syrian Arab Republic | 740303 (655974-840835) | 8697.8 (7659.18-9798.43) | 1232817 (1072153-1386397) | 8633.95  (7568.04-9718.58) | -0.04  (-0.06 to -0.02) |
| Taiwan (Province of China) | 1330241 (1263151-1404172) | 7004.3 (6665.12-7385.11) | 2863952 (2776572-2911707) | 8258.66  (7982.88-8396.91) | 0.64 (0.55-0.73) |
| Tajikistan | 319298 (283031-360943) | 8931 (7837.05-10005.83) | 689712 (601982-782154) | 8587.71  (7460.01-9576.45) | -0.14  (-0.15 to -0.13) |
| Thailand | 2341629 (2049835-2667957) | 4944.73 (4321.54-5588.8) | 4771838 (4142456-5430805) | 5169.73  (4487.19-5814.51) | 0.21 (0.13-0.29) |
| Timor-Leste | 27853 (24310-32166) | 5621.86  (4927.51-6302.13) | 56496 (49497-64251) | 5359.99 (4682.68-6055.3) | -0.18  (-0.21 to -0.15) |
| Togo | 150749 (132194-172582) | 7272.07  (6340.21-8184.99) | 405343 (354535-460942) | 6874.79 (5976.23-7729.5) | -0.23  (-0.28 to -0.17) |
| Tokelau | 87 (76-98) | 6465.99 (5613.1-7296.48) | 91 (78-102) | 6376.61  (5506.68-7174.26) | -0.02  (-0.04 to -0.01) |
| Tonga | 4669 (4060-5299) | 6759.51  (5852.55-7610.49) | 5819 (5082-6598) | 6574.56  (5734.47-7435.66) | -0.12 (-0.13 to -0.1) |
| Trinidad and Tobago | 63461 (55667-72390) | 6151.52  (5350.04-7007.86) | 103430 (89977-117281) | 6095.78  (5370.37-6852.24) | -0.02  (-0.03 to -0.01) |
| Tunisia | 566069 (503965-632736) | 8451.1 (7532.5-9384.72) | 1121765 (977545-1259229) | 8636.15  (7602.35-9694.83) | 0.05 (0.03-0.07) |
| Turkmenistan | 235057 (207139-266670) | 9201.3  (8032.69-10366.62) | 420977 (365155-477478) | 8907.22  (7777.96-9972.36) | -0.09 (-0.1 to -0.08) |
| Tuvalu | 513 (444-587) | 6621.68  (5776.23-7480.78) | 736 (641-837) | 6453.62  (5641.32-7300.41) | -0.06  (-0.08 to -0.05) |
| Türkiye | 4275267 (3884174-4711310) | 9066.15  (8245.85-9972.51) | 8183785 (7130592-9147088) | 8879.73  (7790.75-9931.43) | 0 (-0.07-0.08) |
| Uganda | 780458 (685153-883756) | 7979.11  (6962.65-8979.14) | 1940680 (1707310-2213542) | 7741.54  (6784.36-8662.54) | -0.11 (-0.13 to -0.1) |
| Ukraine | 8163118 (7201869-9108413) | 12894.94  (11390.49-14405.76) | 7593441 (6720327-8474902) | 12359.34  (10971.7-13778.44) | -0.12  (-0.15 to -0.08) |
| United Arab Emirates | 105601 (90538-123761) | 7199.25  (6354.94-8053.34) | 786524 (659566-924763) | 7396.24  (6507.46-8276.71) | 0.05 (0.01-0.09) |
| United Kingdom | 6469334 (5718073-7189092) | 9453.68  (8371.54-10619.93) | 8581321 (7522462-9553824) | 9500.7  (8329.38-10674.23) | 0.16 (0.1-0.21) |
| United Republic of Tanzania | 1210922 (1073775-1366178) | 7752.75  (6778.51-8658.44) | 2899612 (2540841-3286702) | 7515.87  (6572.57-8463.93) | -0.08 (-0.1 to -0.07) |
| United States of America | 32285867  (28788416-35821909) | 11417.82  (10185.35-12721.07) | 45116389  (42173149-47890790) | 10670.05  (9968.49-11340.01) | -0.06 (-0.12-0) |
| United States Virgin Islands | 6110 (5328-6954) | 6076.15  (5295.53-6868.31) | 7523 (6556-8449) | 6061.27  (5312.05-6837.35) | -0.01  (-0.02-0.01) |
| Uruguay | 309495 (273784-344083) | 9142.01  (8069.91-10195.86) | 412968 (362875-460156) | 9881.71  (8658.53-11091.24) | 0.21 (0.16-0.27) |

| Uzbekistan | 1369655 (1215646-1548123) | 9313.07  (8139.6-10449.41) | 2897121 (2535147-3272811) | 9170.37  (8046.19-10282.73) | 0 (-0.02-0.01) |
| --- | --- | --- | --- | --- | --- |
| Vanuatu | 6644 (5877-7565) | 6871.83 (6005.1-7734.26) | 16702 (14671-18997) | 7012.46  (6112.64-7887.02) | 0.06 (0.03-0.1) |
| Venezuela (Bolivarian Republic of) | 1054469 (927629-1215722) | 7198.76 (6308.44-8178.9) | 2018887 (1761622-2280973) | 6961.07 (6078.9-7821.3) | -0.12  (-0.14 to -0.11) |
| Viet Nam | 2940353 (2587925-3280062) | 5998.19  (5311.46-6673.17) | 6274500 (5439049-7141392) | 5919.46  (5169.18-6709.04) | 0.02 (-0.01-0.06) |
| Yemen | 740527 (651153-841050) | 8871.11  (7751.41-9908.56) | 2104836 (1862068-2397745) | 8506.43  (7548.14-9555.91) | -0.17  (-0.18 to -0.15) |
| Zambia | 284929 (251514-322219) | 6340.58  (5627.21-7103.54) | 839726 (732953-958237) | 6954.99  (6066.08-7787.35) | 0.15 (0.09-0.22) |
| Zimbabwe | 415818 (367368-466255) | 6932.1 (6095.86-7701.41) | 768848 (674000-867083) | 7412.46 (6471.6-8305.34) | 0.27 (0.25-0.28) |

ASR, age-standardized rate; LBP, low back pain; UI, uncertainty interval, CI, confdence interval; EAPC, estimated annual percentage change.
