# Supplementary material for: Epidemiological trends and geographic disparities in low back pain burden based on the 2021 GBD study: A cross-sectional analysis
Source: Medicine (Baltimore). 2026 Jun 12;105(24):e49201. doi: 10.1097/MD.0000000000049201 (PMC13268564; doi:10.1097/MD.0000000000049201)
Supplement: Supplementary file 4 [file medi-105-e49201-s004.docx]

| Table S4. The incident cases and ASR for LBP in 204 countries and territories between 1990 and 2021, and its temporal trends. | | | | | |
| --- | --- | --- | --- | --- | --- |
| **Location** | **1990** | | **2021** | | **EAPC (95% CI)**  **1990-2021** |
|  | \| **Cases (95% UI) ASR per 100 000 (95% UI)** \| **Cases (95% UI) ASR per 100 000 (95% UI)** \| \| --- \| --- \| | **ASR per 100 000 (95% UI)** | **Cases (95% UI)** | **ASR per 100 000 (95% UI)** |  |
| **Global** | 165063882  (145785270-185933884) | 3534.99 (3133.04-3960.99) | 266873321  (235369489-299406380) | 3176.63 (2811.82-3562.29) | -0.29 (-0.32 to -0.26) |
| **Country** |  |  |  |  |  |
| Afghanistan | 283230 (248804-317563) | 3628.83 (3219.91-4103.38) | 763858 (660141-866459) | 3648.4 (3213.24-4123.2) | 0.01 (-0.01-0.02) |
| Albania | 144445 (127283-164169) | 5166.37 (4575.53-5815.59) | 173589 (151725-193704) | 5171.35 (4567.64-5848.59) | 0 (-0.01-0) |
| Algeria | 680749 (593215-773819) | 3599.51 (3162.44-4096.98) | 1513180 (1336387-1706644) | 3574.04 (3156.27-4026.17) | -0.04 (-0.05 to -0.02) |
| American Samoa | 977 (856-1121) | 2814.56 (2461.9-3186.64) | 1399 (1214-1587) | 2770.28 (2426.17-3132.16) | -0.04 (-0.06 to -0.03) |
| Andorra | 2463 (2164-2791) | 4062.48 (3585.81-4593.23) | 4617 (4022-5187) | 3937.23 (3487.54-4430.03) | -0.07 (-0.08 to -0.06) |
| Angola | 220228 (191524-250373) | 3376.27 (2960.29-3826.45) | 670584 (585007-768633) | 3256.05 (2874.95-3682.81) | -0.14 (-0.17 to -0.11) |
| Antigua and Barbuda | 1468 (1296-1659) | 2617.54 (2290.5-2949.26) | 2704 (2352-3071) | 2603.26 (2283.6-2945.78) | -0.02 (-0.04 to -0.01) |
| Argentina | 1350405 (1195288-1526846) | 4172.45 (3697.91-4719.77) | 2083325 (1842225-2339082) | 4121.75 (3641.43-4638.04) | -0.02 (-0.06-0.02) |
| Armenia | 128681 (113142-145461) | 4127.39 (3619.41-4653.58) | 152558 (133728-173186) | 4132.29 (3659.25-4672.38) | 0.02 (0.02-0.03) |
| Australia | 923294 (822051-1045980) | 5025.48 (4495.85-5709.17) | 1482484 (1308569-1656553) | 4665.14 (4129.02-5233.03) | -0.16 (-0.19 to -0.14) |
| Austria | 360907 (318378-402755) | 3821.97 (3380.36-4293.43) | 439080 (381510-494633) | 3575.91 (3137.65-3999.45) | -0.07 (-0.12 to -0.02) |
| Azerbaijan | 237870 (209614-270410) | 3903.25 (3441.23-4393.8) | 444581 (389986-509756) | 3925.38 (3472.61-4427.53) | 0.05 (0.04-0.06) |
| Bahamas | 5747 (5016-6504) | 2623.74 (2290.78-2955.25) | 11202 (9844-12754) | 2612.99 (2296.92-2954.23) | -0.03 (-0.04 to -0.02) |
| Bahrain | 14329 (12279-16607) | 3500.43 (3093.07-3973.9) | 53328 (46125-61308) | 3452.89 (3036.39-3866.39) | -0.05 (-0.07 to -0.04) |
| Bangladesh | 2852974 (2542304-3240359) | 3893.01 (3468.87-4409.64) | 5855108 (5159977-6637268) | 3696.16 (3251.54-4180.37) | -0.12 (-0.17 to -0.08) |
| Barbados | 7053 (6255-7942) | 2658.48 (2343.34-3001.85) | 10311 (9029-11637) | 2628.71 (2307.02-2963.17) | -0.03 (-0.04 to -0.03) |
| Belarus | 527737 (464315-595739) | 4477.18 (3939.87-5053.86) | 559125 (490635-628859) | 4457.88 (3949.39-5007.89) | 0 (-0.01-0) |
| Belgium | 505640 (446320-568645) | 4175.31 (3706.01-4731.36) | 615778 (540304-689454) | 4062.1 (3591.99-4569.05) | -0.07 (-0.08 to -0.06) |
| Belize | 3534 (3094-3979) | 2653.6 (2329.38-3001.37) | 10499 (9214-11860) | 2663.04 (2334.58-3007.91) | 0.03 (0.02-0.04) |
| Benin | 92100 (81080-104443) | 3130.54 (2746.19-3540.63) | 257773 (225982-292108) | 2991.77 (2627.95-3366.14) | -0.15 (-0.19 to -0.12) |
| Bermuda | 1756 (1538-2008) | 2695.33 (2369.62-3046.84) | 2423 (2102-2756) | 2663.36 (2342.45-3034.71) | -0.04 (-0.05 to -0.04) |
| Bhutan | 15323 (13434-17318) | 3648.19 (3219.31-4095.51) | 26195 (23057-29894) | 3578.73 (3161.51-4052.08) | -0.06 (-0.06 to -0.05) |
| Bolivia (Plurinational State of) | 123969 (108627-140053) | 2638.84 (2305.86-2964.44) | 292923 (258065-333401) | 2667.58 (2353.16-3037.04) | 0.02 (-0.02-0.05) |
| Bosnia and Herzegovina | 225751 (199134-255132) | 4903.52 (4340.48-5493.18) | 223106 (196219-250665) | 4991.8 (4457.19-5623.81) | 0.05 (0.04-0.07) |
| Botswana | 23715 (20723-26571) | 2844.47 (2497.47-3217.79) | 57601 (50175-66036) | 2813.18 (2454.09-3178.56) | -0.03 (-0.04 to -0.01) |
| Brazil | 4763353 (4167395-5431370) | 3794.63 (3343.85-4291.17) | 9426567  (8304552-10610219) | 3873.61 (3416.6-4355.03) | 0.04 (0.02-0.06) |
| Brunei Darussalam | 8154 (7033-9297) | 3891.56 (3446.93-4357.9) | 17649 (15449-20151) | 3730.92 (3291.22-4221.97) | -0.11 (-0.13 to -0.09) |
| Bulgaria | 533760 (468894-602590) | 5093.31 (4514.99-5792.57) | 478411 (418532-539349) | 5013.54 (4412.83-5638.08) | -0.05 (-0.05 to -0.04) |
| Burkina Faso | 185432 (162443-207181) | 3101.31 (2740.03-3465.15) | 440766 (385102-500134) | 3003.88 (2632.77-3408.28) | -0.15 (-0.18 to -0.12) |
| Burundi | 117464 (102756-133607) | 3376.7 (2971.92-3836.16) | 272446 (237776-309421) | 3206.63 (2806.25-3603.47) | -0.2 (-0.22 to -0.19) |
| Cabo Verde | 7435 (6529-8431) | 2993.49 (2626.91-3396.25) | 15110 (13159-17110) | 2812.93 (2456.78-3172.25) | -0.22 (-0.26 to -0.18) |
| Cambodia | 189340 (165445-215200) | 2819.37 (2475.89-3183.98) | 401347 (349565-455754) | 2626.13 (2304.62-2967.77) | -0.24 (-0.25 to -0.23) |
| Cameroon | 215116 (189284-243504) | 3212.41 (2819.4-3597.36) | 655123 (580004-746966) | 3062.43 (2700.48-3470.95) | -0.18 (-0.22 to -0.15) |
| Canada | 1262603 (1113066-1432253) | 4147.99 (3666.56-4720.44) | 1842152 (1614666-2066842) | 3816.85 (3352.25-4313.39) | -0.18 (-0.21 to -0.15) |
|  |  |  |  |  |  |

| Central African Republic | 59409 (52157-67437) | | | | | | 3334.42 (2941.47-3742.03) | | | | | 123057 (108266-139601) | | | | | 3282.66 (2912.09-3717.92) | | | | | -0.07 (-0.08 to -0.05) | | | | |  |  |
| --- | --- | --- | --- | --- | --- | --- | --- | --- | --- | --- | --- | --- | --- | --- | --- | --- | --- | --- | --- | --- | --- | --- | --- | --- | --- | --- | --- | --- |
| Chad | 133116 (118007-150086) | | | | | | 3446.82 (3035.84-3916.04) | | | | | 327234 (287064-371603) | | | | | 3201.86 (2811.17-3624.86) | | | | | -0.22 (-0.29 to -0.15) | | | | |  |  |
| Chile | 516950 (455193-582712) | | | | | | 4213.32 (3733.46-4751.91) | | | | | 920387 (810575-1034465) | | | | | 4166.18 (3690.56-4690.88) | | | | | -0.03 (-0.07-0) | | | | |  |  |
| China | 29843970  (26065824-34012369) | | | | | | 2859.38 (2508.61-3225.53) | | | | | 43374995  (37494376-49159184) | | | | | 2342.46 (2058.05-2639.32) | | | | | -0.47 (-0.56 to -0.37) | | | | |  |  |
| Colombia | 903584 (789482-1025737) | | | | | | 3383.77 (2979.52-3809.18) | | | | | 1808891 (1606166-2042895) | | | | | 3379.93 (3001.54-3827.31) | | | | | 0.02 (0-0.03) | | | | |  |  |
| Comoros | 9119 (8013-10231) | | | | | | 3088.52 (2738.4-3466.15) | | | | | 19459 (17069-22015) | | | | | 3086.63 (2699.23-3485.88) | | | | | -0.05 (-0.07 to -0.03) | | | | |  |  |
| Congo | 50455 (44436-56888) | | | | | | 3171.34 (2812.83-3570.09) | | | | | 132027 (115674-150889) | | | | | 3135.91 (2766.23-3532.56) | | | | | -0.06 (-0.08 to -0.05) | | | | |  |  |
| Cook Islands | 432 (376-489) | | | | | | 2769.7 (2427.84-3137.24) | | | | | 609 (531-688) | | | | | 2817.3 (2478.52-3189.97) | | | | | 0.09 (0.08-0.1) | | | | |  |  |
| Costa Rica | 81358 (70986-92768) | | | | | | 3270.2 (2877.3-3750.16) | | | | | 168302 (147931-189430) | | | | | 3194.58 (2822.58-3589.83) | | | | | -0.06 (-0.07 to -0.06) | | | | |  |  |
| Croatia | 281153 (249276-317681) | | | | | | 4996.03 (4461.57-5628.07) | | | | | 288442 (250497-324313) | | | | | 4920.53 (4339.57-5551.98) | | | | | -0.03 (-0.07-0) | | | | |  |  |
| Cuba | 292053 (255511-330655) | | | | | | 2661.94 (2329.27-3008.08) | | | | | 378545 (336935-421999) | | | | | 2532.31 (2269.46-2801.52) | | | | | -0.07 (-0.1 to -0.05) | | | | |  |  |
| Cyprus | 33568 (29442-37793) | | | | | | 4113.71 (3607.29-4626.26) | | | | | 68374 (59812-77134) | | | | | 4030.86 (3557.5-4573.67) | | | | | -0.06 (-0.07 to -0.05) | | | | |  |  |
| Czechia | 641839 (564408-724080) | | | | | | 5370.63 (4742.2-6042.4) | | | | | 757515 (660732-856685) | | | | | 5240.01 (4603.17-5944.22) | | | | | -0.08 (-0.09 to -0.08) | | | | |  |  |
| Cote d’Ivoire | 235578 (206609-267183) | | | | | | 3160.52 (2782.23-3560.46) | | | | | 585762 (514674-670846) | | | | | 3042.66 (2665.81-3418.73) | | | | | -0.12 (-0.17 to -0.08) | | | | |  |  |
| Democratic People's Republic of Korea | 586649 (511548-664668) | | | | | | 3090.73 (2693.81-3477.81) | | | | | 919260 (799725-1045842) | | | | | 2897.39 (2550.15-3263.16) | | | | | -0.23 (-0.25 to -0.21) | | | | |  |  |
| Democratic Republic of the Congo | 819174 (713904-928844) | | | | | | 3376.22 (2948.11-3807.59) | | | | | 1996433 (1761131-2272214) | | | | | 3272.37 (2886.54-3697.98) | | | | | -0.12 (-0.15 to -0.09) | | | | |  |  |
| Denmark | 272907 (236380-312312) | | | | | | 4340.92 (3763.84-4983.61) | | | | | 302691 (254278-354091) | | | | | 3907.44 (3319.52-4599.9) | | | | | -0.48 (-0.56 to -0.4) | | | | |  |  |
| Djibouti | 8065 (7028-9143) | | | | | | 3090.16 (2722.52-3472.19) | | | | | 29734 (26266-34110) | | | | | 2940.52 (2581.14-3320.21) | | | | | -0.19 (-0.2 to -0.18) | | | | |  |  |
| Dominica | 1722 (1510-1931) | | | | | | 2714.77 (2382.02-3062.64) | | | | | 2030 (1781-2301) | | | | | 2635.05 (2322.98-2987.04) | | | | | -0.12 (-0.13 to -0.11) | | | | |  |  |
| Dominican Republic | 144783 (127014-163024) | | | | | | 2614.82 (2306.61-2931.52) | | | | | 291607 (256609-331313) | | | | | 2672 (2351.56-3026.82) | | | | | 0.05 (0.03-0.07) | | | | |  |  |
| Ecuador | 194732 (171858-218099) | | | | | | 2535.85 (2245.48-2844.29) | | | | | 428993 (379676-480955) | | | | | 2430.22 (2150.88-2727.19) | | | | | -0.15 (-0.23 to -0.07) | | | | |  |  |
| Egypt | 1531588 (1337336-1741248) | | | | | | 3594.42 (3190.26-4062.13) | | | | | 3317200 (2880493-3762644) | | | | | 3671.83 (3224.33-4137.74) | | | | | 0.07 (0.05-0.1) | | | | |  |  |
| El Salvador | 132194 (114870-149505) | | | | | | 3218.45 (2816.2-3634.58) | | | | | 207220 (183196-235931) | | | | | 3265.02 (2888.89-3724.13) | | | | | 0.08 (0.07-0.09) | | | | |  |  |
| Equatorial Guinea | 9236 (8134-10458) | | | | | | 3336.86 (2943.04-3743.7) | | | | | 32004 (28079-36364) | | | | | 3179.37 (2808.3-3585.74) | | | | | -0.18 (-0.2 to -0.16) | | | | |  |  |
| Eritrea | 59919 (52460-67791) | | | | | | 2971.95 (2612.74-3328.39) | | | | | 137184 (119912-155942) | | | | | 2970.03 (2606.56-3337.55) | | | | | 0.03 (0.01-0.04) | | | | |  |  |
| Estonia | 80756 (71582-90519) | | | | | | 4432.32 (3952.91-4943.6) | | | | | 80918 (71101-91316) | | | | | 4409.91 (3888.54-4941.14) | | | | | 0 (-0.01-0.02) | | | | |  |  |
| Eswatini | 12279 (10740-13741) | | | | | | 2633.68 (2329.51-2949.08) | | | | | 21763 (19117-24709) | | | | | 2591.16 (2278.19-2909.88) | | | | | -0.09 (-0.12 to -0.06) | | | | |  |  |
| Ethiopia | 1052933 (918645-1188316) | | | | | | 3413.41 (3003.48-3830.14) | | | | | 2310711 (2017340-2628932) | | | | | 3184.86 (2799.69-3597.67) | | | | | -0.23 (-0.24 to -0.22) | | | | |  |  |
| Fiji | 15794 (13789-18058) | | | | | | 2789.97 (2447.3-3160.44) | | | | | 23876 (20886-27151) | | | | | 2708.91 (2371.3-3045.83) | | | | | -0.09 (-0.1 to -0.09) | | | | |  |  |
| Finland | 231204 (204155-259034) | | | | | | 3834.44 (3411.72-4283.88) | | | | | 283434 (248428-320363) | | | | | 3664.55 (3234-4088.35) | | | | | -0.1 (-0.12 to -0.08) | | | | |  |  |
| France | 2750833 (2443859-3074845) | | | | | | 4105.18 (3654.69-4613.02) | | | | | 3561233 (3151402-3977792) | | | | | 4059.39 (3594.93-4550.5) | | | | | -0.04 (-0.05 to -0.02) | | | | |  |  |
| Gabon | 22529 (19651-25290) | | | | | | 3141.76 (2768.61-3553.04) | | | | | 45379 (39868-51709) | | | | | 3117.89 (2741.95-3513.15) | | | | | -0.03 (-0.04 to -0.02) | | | | |  |  |
| Gambia | 17471 (15254-19794) | | | | | | 2923.58 (2556.71-3303.35) | | | | | 45638 (40159-51457) | | | | | 2839.33 (2480.86-3198.53) | | | | | -0.11 (-0.15 to -0.07) | | | | |  |  |
| Georgia | 222844 (196652-250922) | | | | | | 3747.78 (3320.39-4199.35) | | | | | 172356 (151079-194886) | | | | | 3705.43 (3276.22-4183.33) | | | | | -0.11 (-0.14 to -0.07) | | | | |  |  |
| Germany | 4663145 (4097906-5296776) | | | | | | 4711.39 (4185.1-5374.12) | | | | | 5280955 (4649960-5946989) | | | | | 4479.42 (3974.85-5045.12) | | | | | -0.1 (-0.12 to -0.08) | | | | |  |  |
| Ghana | 281544 (248696-318542) | | | | | | 2896.17 (2574.57-3228.31) | | | | | 704425 (623088-790114) | | | | | 2752.73 (2452.62-3085.33) | | | | | -0.19 (-0.22 to -0.16) | | | | |  |  |
| Greece | 491501 (435135-548813) | | | | | | 3952.39 (3505.27-4404.25) | | | | | 560188 (490340-631150) | | | | | 3888.68 (3413.09-4415.72) | | | | | -0.07 (-0.09 to -0.04) | | | | |  |  |
| Greenland | 2104 (1829-2428) | | | | | | 3968.4 (3490.22-4518.96) | | | | | 2414 (2082-2743) | | | | | 3748.66 (3285.91-4249.96) | | | | | -0.11 (-0.15 to -0.08) | | | | |  |  |
| Grenada | 1940 (1720-2184) | | | | | | 2662.97 (2332.81-3007.29) | | | | | 2974 (2572-3361) | | | | | 2623.42 (2290.17-2949.02) | | | | | -0.04 (-0.05 to -0.03) | | | | |  |  |
| Guam | 3113 (2718-3558) | | | | | | 2751.46 (2418.11-3125.36) | | | | | 5037 (4393-5713) | | | | | 2727.98 (2400.36-3096.88) | | | | | -0.02 (-0.03-0) | | | | |  |  |
| Guatemala | 208115 (183619-236915) | | | | | | 3625.92 (3206.64-4102.08) | | | | | 495472 (430485-561464) | | | | | 3492.2 (3052.2-3947.77) | | | | | -0.08 (-0.12 to -0.05) | | | | |  |  |
| Guinea | | 129752 (113416-146802) | | | | | 3105.5 (2720.87-3520.65) | | | | 268487 (236777-305016) | | | | | 3051.02 (2672.43-3456.74) | | | | | -0.1 (-0.14 to -0.06) | | | | |  |  |  |
| Guinea-Bissau | | 18630 (16159-21054) | | | | | 3003.59 (2626.42-3397.21) | | | | 38929 (33890-44475) | | | | | 2933.17 (2573.64-3325.83) | | | | | -0.1 (-0.13 to -0.07) | | | | |  |  |  |
| Guyana | | 15729 (13779-17810) | | | | | 2642.41 (2323.95-2985.8) | | | | 19357 (16853-21796) | | | | | 2624.18 (2295.1-2949.78) | | | | | -0.03 (-0.03 to -0.02) | | | | |  |  |  |
| Haiti | | 124618 (108514-140250) | | | | | 2647.29 (2319.37-2986.73) | | | | 283868 (248248-324524) | | | | | 2629.01 (2304.3-2966.16) | | | | | -0.05 (-0.06 to -0.03) | | | | |  |  |  |
| Honduras | | 105632 (91881-119823) | | | | | 3231.48 (2841.01-3645.65) | | | | 288517 (253764-326951) | | | | | 3261.51 (2882.41-3700.26) | | | | | 0.04 (0.03-0.05) | | | | |  |  |  |
| Hungary | | 680167 (597203-768930) | | | | | 5499.6 (4867.26-6202.2) | | | | 711005 (623252-798738) | | | | | 5408.81 (4788.39-6085.51) | | | | | -0.06 (-0.06 to -0.05) | | | | |  |  |  |
| Iceland | | 11473 (10088-12957) | | | | | 4320.83 (3782.84-4889.51) | | | | 17532 (15400-19673) | | | | | 4082.94 (3603.31-4611.27) | | | | | -0.19 (-0.21 to -0.18) | | | | |  |  |  |
| India | | 21034411  (18416350-23841440) | | | | | 3226.78 (2842.31-3618.13) | | | | 38580905  (33754640-43672640) | | | | | 2816.31 (2476.17-3169.09) | | | | | -0.46 (-0.57 to -0.35) | | | | |  |  |  |
| Indonesia | | 3825735 (3337840-4332317) | | | | | 2715.67 (2396.06-3054.22) | | | | 7461578 (6488872-8462645) | | | | | 2637.47 (2322.71-2969.76) | | | | | -0.05 (-0.07 to -0.03) | | | | |  |  |  |
| Iran (Islamic Republic of) | | 1731493 (1513550-1953080) | | | | | 4202.83 (3724.2-4725.21) | | | | 3455438 (3053898-3953725) | | | | | 3879.17 (3430.57-4376.33) | | | | | -0.21 (-0.25 to -0.17) | | | | |  |  |  |
| Iraq | | 471347 (410453-532663) | | | | | 3612.64 (3188.73-4069.9) | | | | 1274423 (1111474-1448221) | | | | | 3570.05 (3138.46-4029.41) | | | | | -0.04 (-0.05 to -0.03) | | | | |  |  |  |
| Ireland | | 157502 (138866-177698) | | | | | 4201.05 (3687.11-4753.08) | | | | 250550 (221415-281437) | | | | | 4140.06 (3683.49-4692.32) | | | | | -0.09 (-0.11 to -0.07) | | | | |  |  |  |
| Israel | | 206499 (183040-232441) | | | | | 4302.53 (3816.42-4839.54) | | | | 422118 (370064-472101) | | | | | 4115.52 (3608.89-4634.83) | | | | | -0.11 (-0.13 to -0.09) | | | | |  |  |  |
| Italy | | 2930897 (2588754-3276821) | | | | | 4162.59 (3684.64-4687.58) | | | | 3554242 (3113030-3997678) | | | | | 4105.26 (3609.92-4634.55) | | | | | -0.09 (-0.11 to -0.08) | | | | |  |  |  |
| Jamaica | | 54223 (47294-60971) | | | | | 2700.91 (2364.63-3064.54) | | | | 82258 (72120-93002) | | | | | 2690.61 (2362.19-3041.34) | | | | | -0.04 (-0.05 to -0.02) | | | | |  |  |  |
| Japan | | 7134687 (6286926-8008476) | | | | | 4808.91 (4269.26-5433.48) | | | | 8085995 (7116421-9043618) | | | | | 4447.66 (3939.3-5022.53) | | | | | -0.18 (-0.21 to -0.15) | | | | |  |  |  |
| Jordan | | 94226 (82031-107005) | | | | | 3633.01 (3182.5-4114.72) | | | | 401633 (350042-458544) | | | | | 3603.73 (3157.81-4061.78) | | | | | -0.03 (-0.03 to -0.02) | | | | |  |  |  |
| Kazakhstan | | 584537 (517013-657812) | | | | | 4007.17 (3553.98-4467.35) | | | | 766073 (669185-872956) | | | | | 4002.02 (3522.07-4522.99) | | | | | -0.03 (-0.05-0) | | | | |  |  |  |
| Kenya | | 476007 (418171-538292) | | | | | 3505.44 (3087.9-3935.78) | | | | 1242811 (1090711-1412001) | | | | | 3423.66 (3015.43-3854.94) | | | | | -0.08 (-0.1 to -0.06) | | | | |  |  |  |
| Kiribati | | 1496 (1306-1707) | | | | | 2815.25 (2473.41-3188.17) | | | | 2830 (2485-3222) | | | | | 2870.53 (2520.54-3250.4) | | | | | 0.12 (0.1-0.15) | | | | |  |  |  |
| Kuwait | | 48509 (41633-55381) | | | | | 3458.13 (3051.65-3874.63) | | | | 175482 (153266-204625) | | | | | 3585.91 (3169.92-4048.18) | | | | | 0.12 (0.11-0.14) | | | | |  |  |  |
| Kyrgyzstan | | 142767 (126299-161543) | | | | | 4023.83 (3539.69-4530.48) | | | | 235054 (205762-267581) | | | | | 3915.41 (3425.12-4407.6) | | | | | -0.09 (-0.09 to -0.08) | | | | |  |  |  |
| Lao People's Democratic Republic | | 75073 (65931-84184) | | | | | 2630.21 (2332.28-2948.6) | | | | 157007 (137312-178968) | | | | | 2496.82 (2196.91-2817.64) | | | | | -0.18 (-0.19 to -0.16) | | | | |  |  |  |
| Latvia | | 137689 (121570-154752) | | | | | 4399.82 (3905.58-4956.1) | | | | 116897 (102079-131701) | | | | | 4361.16 (3838.32-4954.22) | | | | | -0.03 (-0.04 to -0.03) | | | | |  |  |  |
| Lebanon | | 92554 (80733-103962) | | | | | 3552.56 (3116.85-3986.96) | | | | 211322 (186215-239169) | | | | | 3555.98 (3139.45-4009.71) | | | | | 0.04 (0.03-0.06) | | | | |  |  |  |
| Lesotho | | 31066 (27237-35102) | | | | | 2911.15 (2564.73-3291.88) | | | | 40416 (35061-45907) | | | | | 2780.57 (2433.54-3129.07) | | | | | -0.19 (-0.21 to -0.17) | | | | |  |  |  |
| Liberia | | 48888 (43042-55671) | | | | | 3020.05 (2646.45-3410.93) | | | | 111098 (96708-127770) | | | | | 2916.17 (2560.41-3312.35) | | | | | -0.14 (-0.16 to -0.12) | | | | |  |  |  |
| Libya | | 110554 (96513-124867) | | | | | 3571.19 (3154.04-4009.8) | | | | 250734 (220221-286708) | | | | | 3579.67 (3165.03-4021.26) | | | | | -0.02 (-0.03 to -0.01) | | | | |  |  |  |
| Lithuania | | 184691 (163774-208546) | | | | | 4482.92 (3990.9-5055.34) | | | | 172354 (150606-194117) | | | | | 4421.96 (3925.15-4977.35) | | | | | -0.06 (-0.07 to -0.05) | | | | |  |  |  |
| Luxembourg | | 19243 (17027-21783) | | | | | 4192.89 (3710.13-4750.33) | | | | 33131 (29277-37250) | | | | | 4045.76 (3581.16-4576.69) | | | | | -0.11 (-0.12 to -0.1) | | | | |  |  |  |
| Madagascar | | 259493 (227286-294495) | | | | | 3402.5 (2980.79-3836.91) | | | | 618749 (536049-707353) | | | | | 3203.19 (2818.27-3615.79) | | | | | -0.23 (-0.25 to -0.2) | | | | |  |  |  |
| Malawi | | 198948 (175091-223148) | | | | | 3282.88 (2906.75-3683.75) | | | | 409832 (358653-466193) | | | | | 3229.74 (2839.4-3650.59) | | | | | -0.07 (-0.09 to -0.06) | | | | |  |  |  |
| Malaysia | | 334545 (296587-374003) | | | | | 2473.78 (2210.8-2762.4) | | | | 765979 (670064-870864) | | | | | 2387.93 (2089.21-2695.16) | | | | | -0.18 (-0.21 to -0.14) | | | | |  |  |  |
| Maldives | | 3264 (2842-3690) | | | | | 2344.07 (2050.58-2647.86) | | | | 11492 (9947-13404) | | | | | 2215.56 (1944.53-2490.89) | | | | | -0.18 (-0.21 to -0.14) | | | | |  |  |  |
| Mali | | 152736 (133670-171527) | | | | | 2728.58 (2404.83-3075.63) | | | | 404477 (352978-455507) | | | | | 2782.99 (2441.41-3137.26) | | | | | 0.03 (0.01-0.05) | | | | |  |  |  |
| Malta | | 17358 (15309-19561) | | | | | 4282.52 (3790.09-4811.15) | | | | 25751 (22752-29091) | | | | | 4203.72 (3733.48-4757.53) | | | | | -0.09 (-0.1 to -0.07) | | | | |  |  |  |
| Marshall Islands | | 743 (645-848) | | | | | 2718.83 (2379.38-3070.73) | | | | 1288 (1120-1462) | | | | | 2643.36 (2318.49-2985.22) | | | | | -0.1 (-0.1 to -0.09) | | | | |  |  |  |
| Mauritania | | 38448 (33854-42920) | | | | | 2791.51 (2476.47-3126.71) | | | | 85434 (75277-96732) | | | | | 2769.39 (2439.53-3143.33) | | | | | -0.08 (-0.1 to -0.05) | | | | |  |  |  |
| Mauritius | | 24975 (21969-28186) | | | | | 2625.43 (2329.91-2964.99) | | | | 40002 (34794-45523) | | | | | 2481.51 (2184.37-2793.74) | | | | | -0.14 (-0.17 to -0.11) | | | | |  |  |  |
| Mexico | | 1975005 (1720154-2245780) | | | | | 2987.15 (2631.47-3374.96) | | | | 4148822 (3623777-4709984) | | | | | 3076.45 (2698.66-3487.05) | | | | | 0.08 (-0.02-0.17) | | | | |  |  |  |
| Micronesia (Federated States of) | | 1963 (1726-2210) | | | | | 2822.41 (2484.83-3181.61) | | | | 2603 (2265-2987) | | | | | 2826 (2497.14-3223.59) | | | | | 0.02 (0-0.03) | | | | |  |  |  |
| Monaco | | | | 1782 (1563-2005) | | 4098.18 (3635.6-4626.35) | | | | 2203 (1917-2505) | | | | | 3947.85 (3466.44-4434.77) | | | | | -0.1 (-0.11 to -0.09) | | | | |  |  |  |  |
| Mongolia | | | | 59225 (52202-67015) | | 3944.64 (3482.69-4463.19) | | | | 116204 (101693-132992) | | | | | 3869.21 (3399.9-4350.52) | | | | | -0.09 (-0.09 to -0.08) | | | | |  |  |  |  |
| Montenegro | | | | 32680 (28775-37087) | | 5074.86 (4479.04-5735.36) | | | | 39567 (34565-44739) | | | | | 5093.08 (4514.05-5774.75) | | | | | 0.02 (0.01-0.03) | | | | |  |  |  |  |
| Morocco | | | | 806629 (716966-913352) | | 3976.88 (3553.33-4503.64) | | | | 1478445 (1300908-1676720) | | | | | 3938.96 (3477.85-4452.28) | | | | | -0.01 (-0.09-0.06) | | | | |  |  |  |  |
| Mozambique | | | | 298390 (261637-340092) | | 3398.46 (2990.06-3842.31) | | | | 646069 (563264-733398) | | | | | 3357.82 (2953.74-3771.47) | | | | | -0.03 (-0.04 to -0.03) | | | | |  |  |  |  |
| Myanmar | | | | 693040 (603969-782417) | | 2247.19 (1973.73-2531.46) | | | | 1222920 (1076107-1391568) | | | | | 2246.83 (1987.45-2538.18) | | | | | -0.04 (-0.07 to -0.02) | | | | |  |  |  |  |
| Namibia | | | | 26880 (23802-30250) | | 2919.34 (2581.6-3287.04) | | | | 55167 (48879-62154) | | | | | 2887.36 (2549.17-3257.84) | | | | | 0 (-0.01-0.01) | | | | |  |  |  |  |
| Nauru | | | | 200 (176-228) | | 2769.73 (2441.52-3120.82) | | | | 241 (210-274) | | | | | 2846.36 (2504.13-3189.43) | | | | | 0.1 (0.08-0.13) | | | | |  |  |  |  |
| Nepal | | | | 602638 (530743-687151) | | 4309.69 (3817.76-4899.51) | | | | 1150825 (1008007-1315986) | | | | | 4075.32 (3592.08-4648.27) | | | | | -0.14 (-0.19 to -0.09) | | | | |  |  |  |  |
| Netherlands | | | | 651734 (572785-729692) | | 3768.02 (3315.4-4234.09) | | | | 852227 (747588-958469) | | | | | 3650.62 (3226.24-4086.27) | | | | | -0.05 (-0.11-0) | | | | |  |  |  |  |
| New Zealand | | | | 193518 (171748-217889) | | 5277.56 (4666.62-5954.51) | | | | 310891 (276478-347529) | | | | | 4970.24 (4413.58-5599.62) | | | | | -0.12 (-0.15 to -0.1) | | | | |  |  |  |  |
| Nicaragua | | | | 87463 (76102-99466) | | 3298.27 (2916.85-3729.39) | | | | 202382 (177300-231633) | | | | | 3256.79 (2861.65-3694.18) | | | | | -0.01 (-0.02-0) | | | | |  |  |  |  |
| Niger | | | | 140905 (122943-159740) | | 2999.28 (2631.36-3391.22) | | | | 429734 (376911-487723) | | | | | 3033.65 (2667.77-3451.21) | | | | | 0.07 (0.04-0.11) | | | | |  |  |  |  |
| Nigeria | | | | 1889294 (1654211-2133205) | | 3064.49 (2689.12-3457.13) | | | | 4537352 (3952809-5139278) | | | | | 3008.67 (2638.59-3393.45) | | | | | -0.06 (-0.1 to -0.02) | | | | |  |  |  |  |
| Niue | | | | 59 (52-67) | | 2788.36 (2437.53-3143.54) | | | | 53 (46-60) | | | | | 2748.47 (2416.7-3098.05) | | | | | -0.03 (-0.04 to -0.01) | | | | |  |  |  |  |
| North Macedonia | | | | 97574 (85834-110385) | | 4836.36 (4259.85-5463.47) | | | | 135011 (117412-152072) | | | | | 4800.67 (4226.44-5410.39) | | | | | -0.02 (-0.02 to -0.01) | | | | |  |  |  |  |
| Northern Mariana Islands | | | | 1019 (884-1186) | | 2744.39 (2411.36-3105.65) | | | | 1484 (1288-1689) | | | | | 2722.48 (2393.12-3067.97) | | | | | -0.04 (-0.06 to -0.01) | | | | |  |  |  |  |
| Norway | | | | 207414 (183343-233034) | | 4049.05 (3569.57-4556.65) | | | | 268732 (237151-302101) | | | | | 3821.68 (3374.53-4301.65) | | | | | -0.2 (-0.22 to -0.19) | | | | |  |  |  |  |
| Oman | | | | 49823 (43082-57062) | | 3481.89 (3051.75-3935.56) | | | | 146521 (126649-169184) | | | | | 3432.48 (3023.46-3876.56) | | | | | -0.04 (-0.05 to -0.04) | | | | |  |  |  |  |
| Pakistan | | | | 2338401 (2014724-2656278) | | 3059.35 (2638.02-3455.1) | | | | 6112580 (5300163-7034456) | | | | | 3334.44 (2893.5-3769.61) | | | | | 0.36 (0.31-0.42) | | | | |  |  |  |  |
| Palau | | | | 358 (313-406) | | 2756.35 (2428.37-3113.62) | | | | 606 (528-689) | | | | | 2690.78 (2377.65-3017.67) | | | | | -0.06 (-0.08 to -0.04) | | | | |  |  |  |  |
| Palestine | | | | 51045 (44624-57749) | | 3674.8 (3263.27-4161.13) | | | | 148899 (129797-168709) | | | | | 3585.3 (3161.31-4046.14) | | | | | -0.07 (-0.08 to -0.06) | | | | |  |  |  |  |
| Panama | | | | 64005 (56103-72813) | | 3163.82 (2789.12-3575.71) | | | | 139114 (121049-156834) | | | | | 3166.7 (2762.36-3573.88) | | | | | 0.01 (-0.01-0.02) | | | | |  |  |  |  |
| Papua New Guinea | | | | 77360 (67046-88080) | | 2732.78 (2396.83-3106.17) | | | | 214552 (186673-245013) | | | | | 2706.66 (2373.75-3081.35) | | | | | -0.02 (-0.04-0) | | | | |  |  |  |  |
| Paraguay | | | | 100278 (88123-112790) | | 3215.79 (2829.75-3608.52) | | | | 227547 (198599-257032) | | | | | 3304.43 (2884-3728.56) | | | | | 0.08 (0.02-0.14) | | | | |  |  |  |  |
| Peru | | | | 417440 (366126-474798) | | 2481.57 (2174.97-2812.31) | | | | 924518 (817651-1044053) | | | | | 2549.49 (2255.93-2872.07) | | | | | 0.13 (0.1-0.15) | | | | |  |  |  |  |
| Philippines | | | | 1191286 (1043851-1345325) | | 2653.56 (2335.83-2992.76) | | | | 2614041 (2293012-2975585) | | | | | 2585.87 (2284.09-2924.23) | | | | | -0.11 (-0.13 to -0.1) | | | | |  |  |  |  |
| Poland | | | | 2234537 (1982193-2520771) | | 5389.49 (4785.17-6052.63) | | | | 2725099 (2392904-3060457) | | | | | 5274.09 (4671.11-5936.96) | | | | | -0.09 (-0.1 to -0.08) | | | | |  |  |  |  |
| Portugal | | | | 515061 (455283-578100) | | 4368.13 (3871.24-4935.54) | | | | 642656 (565685-722689) | | | | | 4258.91 (3767.48-4771.06) | | | | | -0.11 (-0.13 to -0.09) | | | | |  |  |  |  |
| Puerto Rico | | | | 95521 (84225-108118) | | 2621.66 (2311.84-2971.03) | | | | 120440 (105178-135880) | | | | | 2614.27 (2304.24-2951.9) | | | | | -0.03 (-0.04 to -0.02) | | | | |  |  |  |  |
| Qatar | | | | 12648 (10749-14877) | | 3405.5 (3001.97-3851.81) | | | | 101412 (86426-119345) | | | | | 3467.29 (3051.1-3904.59) | | | | | 0.01 (-0.01-0.03) | | | | |  |  |  |  |
| Republic of Korea | | | | 1752694 (1528643-1999909) | | 4145.58 (3657.69-4686.93) | | | | 2704831 (2366623-3045724) | | | | | 3871.09 (3409.13-4371.71) | | | | | -0.18 (-0.21 to -0.16) | | | | |  |  |  |  |
| Republic of Moldova | | | | 201102 (176920-227443) | | 4457.71 (3931.69-5000.98) | | | | 212503 (185638-240032) | | | | | 4394.68 (3889.95-4962.09) | | | | | -0.06 (-0.07 to -0.05) | | | | |  |  |  |  |
| Romania | | | | 1381514 (1222241-1571008) | | 5343.81 (4734.27-6064.28) | | | | 1320184 (1149042-1490233) | | | | | 5150.54 (4540.8-5828.94) | | | | | -0.15 (-0.17 to -0.14) | | | | |  |  |  |  |
| Russian Federation | | | | 7925263 (7024918-8905018) | | 4667.59 (4154.67-5237.32) | | | | 8715418 (7611600-9775837) | | | | | 4529.36 (4024.02-5083.05) | | | | | -0.02 (-0.05-0) | | | | |  |  |  |  |
| Rwanda | | | | 154480 (135399-176295) | | 3481.04 (3047.82-3936.36) | | | | 329459 (288626-375878) | | | | | 3382.48 (2984.53-3807.93) | | | | | -0.12 (-0.14 to -0.1) | | | | |  |  |  |  |
| Saint Kitts and Nevis | | | | 984 (864-1106) | | 2651.77 (2308.59-2999.54) | | | | 1804 (1554-2057) | | | | | 2593.21 (2264.34-2925.8) | | | | | -0.08 (-0.08 to -0.08) | | | | |  |  |  |  |
| Saint Lucia | | | | 3025 (2634-3421) | | 2761.92 (2408.1-3127.45) | | | | 5740 (5020-6536) | | | | | 2653.96 (2332.07-3004.56) | | | | | -0.14 (-0.14 to -0.13) | | | | |  |  |  |  |
| Saint Vincent and the Grenadines | | | | 2313 (2015-2626) | | 2629.4 (2300.37-2980.84) | | | | 3364 (2943-3808) | | | | | 2584.62 (2261.15-2919.49) | | | | | -0.05 (-0.06 to -0.05) | | | | |  |  |  |  |
| Samoa | | | | 3416 (2967-3873) | | 2919.3 (2536.92-3300.3) | | | | 4847 (4231-5492) | | | | | 2792.47 (2455.98-3144.45) | | | | | -0.18 (-0.21 to -0.16) | | | | |  |  |  |  |
| San Marino | | | | 1173 (1033-1317) | | 4112.63 (3641.15-4650.38) | | | | 1825 (1594-2061) | | | | | 3966.97 (3488.5-4454.38) | | | | | -0.1 (-0.11 to -0.09) | | | | |  |  |  |  |
| Sao Tome and Principe | | | | 2233 (1953-2505) | | 2766.92 (2420.98-3132.88) | | | | 4479 (3950-5096) | | | | | 2680.34 (2372.86-3030.36) | | | | | -0.11 (-0.14 to -0.09) | | | | |  |  |  |  |
| Saudi Arabia | | | | 399358 (348465-453519) | | 3449.65 (3047.04-3890.04) | | | | 1305265 (1122126-1526171) | | | | | 3508.43 (3089.91-3964.15) | | | | | 0.07 (0.06-0.07) | | | | |  |  |  |  |
| Senegal | | | 136060 (119606-152361) | | | | | 2873.89 (2540.8-3223.4) | | | | | 313133 (274859-356947) | | | | | 2765.59 (2431.81-3136.31) | | | | | -0.18 (-0.21 to -0.15) | | | | |  |
| Serbia | | | 553595 (483422-623986) | | | | | 5138.33 (4521.81-5780.77) | | | | | 607813 (533724-682177) | | | | | 5128.06 (4544.18-5764.63) | | | | | -0.01 (-0.02 to -0.01) | | | | |  |
| Seychelles | | | 1552 (1364-1750) | | | | | 2469.14 (2178.98-2794.97) | | | | | 2761 (2392-3156) | | | | | 2334.65 (2050.29-2635.09) | | | | | -0.18 (-0.19 to -0.17) | | | | |  |
| Sierra Leone | | | 87875 (76748-99378) | | | | | 3095.98 (2694.18-3499.95) | | | | | 179134 (157347-202716) | | | | | 2930.75 (2563.55-3303.54) | | | | | -0.19 (-0.22 to -0.16) | | | | |  |
| Singapore | | | 110595 (97525-126162) | | | | | 3589.4 (3188.65-4035.87) | | | | | 244604 (212909-278047) | | | | | 3329.34 (2896.37-3752.56) | | | | | -0.12 (-0.18 to -0.07) | | | | |  |
| Slovakia | | | 295357 (262716-335468) | | | | | 5210.4 (4652.27-5913.21) | | | | | 363252 (318123-410646) | | | | | 5064.94 (4486.86-5752.89) | | | | | -0.09 (-0.09 to -0.08) | | | | |  |
| Slovenia | | | 109828 (96955-123560) | | | | | 4862.21 (4311.94-5469.13) | | | | | 139292 (121172-155746) | | | | | 4806.78 (4240.58-5425.93) | | | | | -0.06 (-0.08 to -0.05) | | | | |  |
| Solomon Islands | | | 5841 (5085-6627) | | | | | 2707.23 (2367.71-3057.46) | | | | | 14709 (12856-16832) | | | | | 2848.4 (2513.32-3234.01) | | | | | 0.22 (0.2-0.24) | | | | |  |
| Somalia | | | 149422 (129926-170664) | | | | | 3249.06 (2857.52-3641.81) | | | | | 399441 (348364-453268) | | | | | 3254.04 (2871.68-3667.98) | | | | | -0.01 (-0.03-0) | | | | |  |
| South Africa | | | 804804 (707604-908396) | | | | | 2948.76 (2591.41-3313.73) | | | | | 1491612 (1302877-1691659) | | | | | 2753.32 (2409.37-3110.09) | | | | | -0.19 (-0.21 to -0.18) | | | | |  |
| South Sudan | | | 120669 (105063-136741) | | | | | 3175.42 (2778.12-3574.4) | | | | | 199796 (175628-226870) | | | | | 3150.86 (2777.5-3534.73) | | | | | -0.04 (-0.05 to -0.02) | | | | |  |
| Spain | | | 1755641 (1583763-1918718) | | | | | 3871.75 (3503.26-4235.24) | | | | | 2247132 (1939446-2561406) | | | | | 3527.47 (3067.2-4007.15) | | | | | -0.07 (-0.18-0.04) | | | | |  |
| Sri Lanka | | | 355361 (313634-400222) | | | | | 2478.71 (2189.89-2764.85) | | | | | 617959 (537796-702447) | | | | | 2447.29 (2147.04-2782.77) | | | | | -0.04 (-0.06 to -0.02) | | | | |  |
| Sudan | | | 527916 (461415-599027) | | | | | 3637.42 (3221.26-4121.84) | | | | | 1234009 (1077083-1399951) | | | | | 3629.6 (3199.68-4073.18) | | | | | -0.01 (-0.02-0) | | | | |  |
| Suriname | | | 8623 (7501-9724) | | | | | 2625.19 (2295.09-2961.71) | | | | | 16591 (14554-18927) | | | | | 2663.9 (2346.95-3028.23) | | | | | 0.06 (0.05-0.07) | | | | |  |
| Sweden | | | 297281 (265443-329656) | | | | | 2647.8 (2383.99-2928.38) | | | | | 447647 (388737-508289) | | | | | 3189.49 (2748.5-3610.93) | | | | | 0.61 (0.46-0.76) | | | | |  |
| Switzerland | | | 376921 (336909-416477) | | | | | 4595.39 (4109.18-5079.51) | | | | | 502393 (438855-563448) | | | | | 4207.13 (3721.63-4728.07) | | | | | -0.14 (-0.22 to -0.05) | | | | |  |
| Syrian Arab Republic | | | 324357 (282729-366280) | | | | | 3677.31 (3258.52-4139.47) | | | | | 524527 (457209-594343) | | | | | 3670.71 (3233.61-4127.71) | | | | | -0.03 (-0.04 to -0.01) | | | | |  |
| Taiwan (Province of China) | | | 581029 (525336-632456) | | | | | 3028.33 (2752.13-3287.23) | | | | | 1122982 (1023766-1231288) | | | | | 3328.52 (3064.8-3601.76) | | | | | 0.37 (0.31-0.44) | | | | |  |
| Tajikistan | | | 141332 (124307-159247) | | | | | 3832.94 (3382.17-4306.88) | | | | | 306405 (270417-348287) | | | | | 3725.9 (3290.62-4202.04) | | | | | -0.1 (-0.11 to -0.1) | | | | |  |
| Thailand | | | 1069444 (933052-1205036) | | | | | 2224.75 (1950.08-2489.64) | | | | | 2075125 (1796308-2348951) | | | | | 2284.91 (1998.55-2581.55) | | | | | 0.14 (0.08-0.2) | | | | |  |
| Timor-Leste | | | 12554 (10972-14313) | | | | | 2458.4 (2179.58-2768.14) | | | | | 25279 (21919-28444) | | | | | 2349.44 (2056.88-2653.1) | | | | | -0.17 (-0.19 to -0.15) | | | | |  |
| Togo | | | 67479 (59130-77209) | | | | | 3117.49 (2749.59-3528.76) | | | | | 179790 (157054-204836) | | | | | 2963.73 (2584.15-3372.06) | | | | | -0.2 (-0.25 to -0.15) | | | | |  |
| Tokelau | | | 38 (33-43) | | | | | 2794.71 (2447.23-3165.37) | | | | | 39 (34-44) | | | | | 2750.05 (2416.83-3111.14) | | | | | -0.04 (-0.05 to -0.02) | | | | |  |
| Tonga | | | 2044 (1796-2308) | | | | | 2882.44 (2523.31-3255.96) | | | | | 2518 (2202-2824) | | | | | 2813.36 (2470.47-3164.51) | | | | | -0.09 (-0.11 to -0.08) | | | | |  |
| Trinidad and Tobago | | | 27901 (24472-31496) | | | | | 2666.52 (2343.9-2992.84) | | | | | 44414 (38858-50338) | | | | | 2646.64 (2344.5-3001.13) | | | | | -0.02 (-0.03 to -0.02) | | | | |  |
| Tunisia | | | 245008 (215949-273068) | | | | | 3582.35 (3173.62-3997.29) | | | | | 472177 (416620-536382) | | | | | 3647.82 (3228.84-4122.66) | | | | | 0.05 (0.03-0.06) | | | | |  |
| Turkmenistan | | | 103498 (90493-117517) | | | | | 3919.63 (3468.78-4428.66) | | | | | 182615 (160669-207483) | | | | | 3817.34 (3376.71-4295.52) | | | | | -0.07 (-0.08 to -0.06) | | | | |  |
| Tuvalu | | | 224 (195-254) | | | | | 2846.31 (2497.42-3207.47) | | | | | 319 (279-362) | | | | | 2776.87 (2439.6-3142.38) | | | | | -0.07 (-0.09 to -0.06) | | | | |  |
| Türkiye | | | 1834059 (1638714-2034321) | | | | | 3815.23 (3409.68-4244.43) | | | | | 3441181 (2993295-3894469) | | | | | 3750.6 (3292.49-4240.37) | | | | | -0.01 (-0.06-0.04) | | | | |  |
| Uganda | | | 344197 (300477-384703) | | | | | 3363.65 (2973.22-3774.87) | | | | | 858911 (747682-966493) | | | | | 3266.14 (2860.24-3676.74) | | | | | -0.1 (-0.11 to -0.08) | | | | |  |
| Ukraine | | | 3189021 (2789511-3570582) | | | | | 5114.95 (4476.43-5711.31) | | | | | 2982381 (2583005-3333472) | | | | | 4999.04 (4411.44-5580.78) | | | | | -0.06 (-0.08 to -0.04) | | | | |  |
| United Arab Emirates | | | 47357 (40140-54898) | | | | | 3176.98 (2807.11-3536.47) | | | | | 341686 (288669-406707) | | | | | 3252.99 (2867.83-3638.56) | | | | | 0.04 (0.01-0.08) | | | | |  |
| United Kingdom | | | 2721874 (2413854-3060480) | | | | | 4035.5 (3581.63-4552.43) | | | | | 3581700 (3160238-4017754) | | | | | 4054.15 (3580.24-4553.96) | | | | | 0.16 (0.11-0.21) | | | | |  |
| United Republic of Tanzania | | | 536448 (469851-601762) | | | | | 3300.88 (2901.91-3703.64) | | | | | 1279864 (1127902-1449323) | | | | | 3204.24 (2821.02-3617.48) | | | | | -0.08 (-0.09 to -0.07) | | | | |  |
| United States of America | | | 13405567  (11924745-15104332) | | | | | 4774.6 (4252.5-5367.92) | | | | | 18681080  (16946961-20396397) | | | | | 4501.63 (4092.82-4911.62) | | | | | -0.05 (-0.11-0.01) | | | | |  |
| United States Virgin Islands | | | 2678 (2361-3046) | | | | | 2645.26 (2344.73-2991.05) | | | | | 3190 (2742-3632) | | | | | 2630.06 (2312.54-2981.11) | | | | | -0.01 (-0.03-0) | | | | |  |
| Uruguay | | | 133523 (118205-149381) | | | | | 3966.03 (3504.26-4470.33) | | | | | 172071 (150937-192804) | | | | | 4191.52 (3697.03-4715.89) | | | | | 0.17 (0.12-0.21) | | | | |  |
| Uzbekistan | | | 598472 (524527-678144) | | | | | 3952.97 (3494.02-4460.08) | | | | | 1251493 (1092789-1427296) | | | | | 3910.79 (3446.23-4416.18) | | | | | -0.01 (-0.02-0) | | | | |  |
| Vanuatu | | | 2920 (2546-3313) | | | | | 2913.86 (2562.97-3293.35) | | | | | 7179 (6250-8207) | | | | | 2945.24 (2584.51-3354.19) | | | | | 0.03 (0.01-0.06) | | | | |  |
| Venezuela (Bolivarian Republic of) | | | | | 464834 (406034-527830) | | | | 3103.49 (2721.36-3498.04) | | | | | 869800 (755456-976102) | | | | | 3014.83 (2623.93-3381.14) | | | | | -0.11 (-0.12 to -0.1) | | | | |
| Viet Nam | | | | | 1303478 (1146810-1462674) | | | | 2590.65 (2286.95-2922.66) | | | | | 2682058 (2328042-3080451) | | | | | 2530.99 (2216.62-2879.86) | | | | | -0.03 (-0.05-0) | | | | |
| Yemen | | | | | 325650 (284449-370197) | | | | 3725.76 (3289.51-4223.78) | | | | | 923386 (803672-1044363) | | | | | 3627.32 (3200.54-4102.4) | | | | | -0.11 (-0.12 to -0.1) | | | | |
| Zambia | | | | | 132412 (116668-147882) | | | | 2832.73 (2504.63-3168.58) | | | | | 379563 (333590-429989) | | | | | 3020.48 (2670.15-3409.18) | | | | | 0.09 (0.04-0.14) | | | | |
| Zimbabwe | | | | | 186411 (164395-208943) | | | | 2979.95 (2635.72-3356.38) | | | | | 333686 (292626-377136) | | | | | 3104.5 (2717.27-3514.34) | | | | | 0.16 (0.15-0.17) | | | | |

ASR, age-standardized rate; LBP, low back pain; UI, uncertainty interval, CI, confdence interval; EAPC, estimated annual percentage change; DALYs, disability-adjusted life years.
