# Supplementary material for: Epidemiological trends and geographic disparities in low back pain burden based on the 2021 GBD study: A cross-sectional analysis
Source: Medicine (Baltimore). 2026 Jun 12;105(24):e49201. doi: 10.1097/MD.0000000000049201 (PMC13268564; doi:10.1097/MD.0000000000049201)
Supplement: Supplementary file 5 [file medi-105-e49201-s005.docx]

| Locations | Percentage Change in the number of cases between 1990 and 2021 | | |
| --- | --- | --- | --- |
|  | Incidence(%) | Prevalence (% | DALYs (%) |
| Qatar | 729.489064 | 836.7635719 | 826.9688377 |
| United Arab Emirates | 682.9113014 | 819.3280503 | 774.877579 |
| Jordan | 346.9080526 | 412.0314955 | 387.5663287 |
| Bahrain | 292.3217365 | 349.1409234 | 335.3015825 |
| Djibouti | 286.7771819 | 327.0558624 | 306.2574644 |
| Kuwait | 283.8304421 | 326.4862552 | 316.4802741 |
| Maldives | 278.0561548 | 335.5139786 | 314.33101 |
| Equatorial Guinea | 257.512515 | 284.6887706 | 285.05963 |
| Saudi Arabia | 246.8079772 | 299.2600401 | 289.6478899 |
| Cameroon | 212.0947888 | 223.2537159 | 221.3995734 |
| Angola | 210.8592667 | 222.1976485 | 214.9719645 |
| Oman | 209.673303 | 264.972392 | 255.6638765 |
| Belize | 208.8640291 | 238.2266107 | 231.7533294 |
| Niger | 204.4708213 | 207.3113773 | 207.3957371 |
| Palestine | 201.5892725 | 235.5951889 | 234.0048957 |
| Yemen | 190.9378516 | 215.8605725 | 212.6320373 |
| Benin | 188.2758702 | 201.5965619 | 199.5694492 |
| Zambia | 185.0212606 | 189.9250135 | 193.304982 |
| Papua New Guinea | 183.7644117 | 194.2720267 | 191.7946614 |
| Iraq | 180.0779771 | 216.1563077 | 210.1824341 |
| Honduras | 179.1351531 | 207.3621451 | 204.3109878 |
| Togo | 175.8052663 | 198.3100447 | 193.0101759 |
| Republic of Congo | 169.4164303 | 180.308529 | 173.4113473 |
| Kenya | 169.0426917 | 188.303989 | 181.5751189 |
| Gambia | 168.3932711 | 185.5555751 | 181.4991817 |
| Somalia | 167.0543358 | 163.9595865 | 163.1481578 |
| Afghanistan | 165.8524276 | 155.0394289 | 173.7879098 |
| Mali | 164.72482 | 162.203361 | 165.5540234 |
| Pakistan | 161.423507 | 172.6784233 | 175.8178474 |
| Ghana | 157.7448623 | 174.7499977 | 172.9152657 |
| Solomon Islands | 156.3375254 | 167.2224223 | 162.3640928 |
| Cote d’Ivoire | 155.5977641 | 171.7413028 | 171.2438478 |
| Uganda | 153.4951647 | 165.7704571 | 167.7010097 |
| Botswana | 153.1544432 | 175.6907536 | 164.6795654 |
| Vanuatu | 151.8909036 | 167.1616321 | 163.9403185 |
| Chad | 151.1203081 | 152.7046889 | 152.9942521 |
| Democratic Republic of the Congo | 146.5360119 | 149.8931827 | 147.2751359 |
| Nigeria | 145.9469416 | 152.0843585 | 154.8504163 |
| Guatemala | 145.6469003 | 179.9139422 | 170.7633794 |
| Madagascar | 143.9659069 | 153.8965161 | 153.6234386 |
| Bolivia | 143.4076008 | 169.6361952 | 162.1054467 |

Table S5. Percentage change in the number of reported cases of the global burden of LBP between 1990 and 2021.

| Tanzania | 143.2791616 | 154.2695335 | 154.116786 |
| --- | --- | --- | --- |
| Malaysia | 143.1469561 | 172.7846945 | 156.6011105 |
| Burkina Faso | 141.2202619 | 147.4533155 | 151.20459 |
| Libya | 140.8886825 | 177.8366123 | 173.0168093 |
| Nicaragua | 140.1054954 | 180.4442045 | 170.1805324 |
| Sudan | 138.6678949 | 157.4064335 | 155.8903593 |
| Singapore | 137.8233269 | 182.123783 | 156.1844634 |
| Senegal | 135.4583937 | 150.2496664 | 148.7240702 |
| Burundi | 135.3532888 | 120.0908712 | 114.9612051 |
| Algeria | 134.8681016 | 174.8659758 | 165.8045608 |
| Liberia | 134.6043141 | 136.5595367 | 132.7585681 |
| Lebanon | 134.065129 | 168.065801 | 169.4831215 |
| Eritrea | 132.1557609 | 159.0546965 | 155.4126222 |
| Haiti | 132.0870756 | 141.4395996 | 135.7994441 |
| Ecuador | 131.7129794 | 162.7873949 | 147.6453378 |
| Paraguay | 130.7298929 | 147.3366442 | 148.3441486 |
| Brunei Darussalam | 130.0330982 | 167.9199725 | 155.3072354 |
| Philippines | 129.4288966 | 150.933501 | 139.0406441 |
| Peru | 127.763675 | 153.2536544 | 146.5143677 |
| Mauritania | 126.9400557 | 138.1771377 | 136.2578241 |
| Ethiopia | 126.2394415 | 143.9458971 | 134.9690512 |
| Panama | 125.0108081 | 151.9767282 | 144.494316 |
| Cambodia | 122.3572566 | 152.1243367 | 138.8257398 |
| Tajikistan | 121.6322258 | 131.5521779 | 128.0994339 |
| Egypt | 121.1739862 | 143.1456737 | 139.7004859 |
| Comoros | 118.7816166 | 137.7052178 | 129.1262989 |
| Laos | 118.1333093 | 144.1072631 | 138.8313678 |
| Rwanda | 117.8802845 | 113.7676263 | 100.0337599 |
| Mozambique | 117.4927639 | 122.6449809 | 122.0369003 |
| Mexico | 117.3638537 | 143.5085379 | 134.3337368 |
| Costa Rica | 116.7124722 | 148.8070406 | 138.1931762 |
| Vietnam | 116.1968365 | 141.6673619 | 130.5471372 |
| Uzbekistan | 115.3426934 | 132.7342804 | 128.5500955 |
| Iran | 114.7008699 | 157.1283852 | 146.690282 |
| Bangladesh | 113.5675099 | 145.5091725 | 139.7042681 |
| Cabo Verde | 113.5462049 | 131.9563947 | 124.998372 |
| Guinea-Bissau | 112.4794115 | 119.5261786 | 121.2620381 |
| Namibia | 110.5733585 | 126.2074594 | 122.0138021 |
| Israel | 109.5862367 | 125.1268486 | 121.9692324 |
| Sierra Leone | 109.3274865 | 112.8550634 | 109.3411268 |
| Central African Republic | 109.3076567 | 107.8546406 | 104.7552528 |
| Cyprus | 109.1815376 | 130.7053331 | 122.4874146 |
| Sao Tome and Principe | 109.1255938 | 119.3132713 | 117.0940897 |
| Guinea | 109.0987311 | 109.8827309 | 115.0044425 |

| Malawi | 108.6021136 | | 115.7009401 | | | 112.3745734 | |  |
| --- | --- | --- | --- | --- | --- | --- | --- | --- |
| Gabon | 107.9560052 | | 119.2327039 | | | 114.7143189 | |  |
| Colombia | 107.6798644 | | 141.9666082 | | | 135.4390249 | |  |
| Timor-Leste | 107.6371221 | | 128.0691983 | | | 120.2396708 | |  |
| Dominican Republic | 107.4101744 | | 132.4534442 | | | 123.3780239 | |  |
| Indonesia | 105.4858099 | | 131.6356811 | | | 123.7075569 | |  |
| Mongolia | 104.1867201 | | 122.9415542 | | | 113.4547625 | |  |
| Brazil | 103.6509136 | | 115.5166663 | | | 107.3039544 | |  |
| Thailand | 103.6269831 | | 119.6393794 | | | 103.4905881 | |  |
| Bahamas | 102.6687157 | | 125.7392969 | | | 113.5361544 | |  |
| Saint Lucia | 101.8182509 | | 138.7701623 | | | 121.2824062 | |  |
| Tunisia | 101.5508727 | | 133.7556061 | | | 125.4876073 | |  |
| Suriname | 98.06775415 | | 120.3091179 | | | 109.655541 | |  |
| Venezuela | 97.33527773 | | 128.9945678 | | | 117.1647394 | |  |
| Türkiye | 97.03862863 | | 128.6802076 | | | 114.3292608 | |  |
| Nepal | 96.81380236 | | 120.90211 | | | 117.2171187 | |  |
| Taiwan | 95.57901503 | | 117.9794299 | | | 121.6285067 | |  |
| India | 95.13742667 | | 130.6513946 | | | 123.8597524 | |  |
| Saint Kitts and Nevis | 94.96581045 | | 114.3327794 | | | 99.02188328 | |  |
| Andorra | 94.72820119 | | 111.0192194 | | | 97.7146952 | |  |
| South Africa | 92.98293886 | | 114.6116203 | | | 102.7346853 | |  |
| Kiribati | 92.54849622 | | 101.0908965 | | | 98.9208329 | |  |
| Antigua and Barbuda | 92.18619081 | | 109.214638 | | | 103.4048614 | |  |
| Seychelles | 91.82734421 | | 113.5838268 | | | 101.3909261 | |  |
| Azerbaijan | 90.9227278 | | 104.5843011 | | | 99.256261 | |  |
| Morocco | 90.52989794 | | 115.9164674 | | | 108.0920388 | |  |
| Chile | 85.20645093 | | 88.56328236 | | | 81.24876417 | |  |
| Myanmar | 84.19056619 | | 106.3638405 | | | 90.65248643 | |  |
| Marshall Islands | 83.73480958 | | 103.7752784 | | | 92.44272051 | |  |
| Turkmenistan | 82.58337114 | | 101.4789618 | | | 93.96631685 | |  |
| Eswatini | 82.49210047 | | 97.98866351 | | | 92.49969706 | |  |
| Palau | 81.60424782 | | 102.5307758 | | | 85.62465387 | |  |
| Sri Lanka | 81.02219182 | | 102.6209954 | | | 90.0029174 | |  |
| Zimbabwe | 79.10880652 | | 83.19157901 | | | 85.38289131 | |  |
| Bhutan | 77.56100578 | | 105.3367275 | | | 102.9886145 | |  |
| Luxembourg | 76.19981817 | | 88.83229354 | | | 82.91114545 | |  |
| Syria | 72.42964911 | | 96.35391453 | | | 83.00667174 | |  |
| Mauritius | 70.91513221 | | 96.22642069 | | | 89.40078832 | |  |
| Kyrgyzstan | 68.78269133 | | 78.64289722 | | | 77.32742126 | |  |
| South Sudan | 68.77852699 | | 70.73445649 | | | 69.71476621 | |  |
| Australia | 68.62527178 | | 89.36397384 | | | 82.50515222 | |  |
| Guam | 68.58942117 | | 91.05510324 | | | 74.61189383 | |  |
| New Zealand | 67.32529224 | | 86.27848991 | | | 77.74216586 | |  |
| South Korea | 66.29529007 | | 107.7244203 | | | 93.16447427 | |  |
| Trinidad and Tobago | | 65.72595806 | 89.84613599 | | 79.50770089 | |  |  |
| Ireland | | 63.6862465 | 77.12843028 | | 71.88542806 | |  |  |
| China | | 63.44215267 | 100.5480707 | | 78.81819191 | |  |  |
| North Korea | | 62.58345143 | 77.73284705 | | 70.24641159 | |  |  |
| El Salvador | | 61.11078608 | 85.18484805 | | 82.94557349 | |  |  |
| Grenada | | 60.89900437 | 77.82046937 | | 68.72088144 | |  |  |
| San Marino | | 60.20819262 | 72.82557231 | | 63.5279898 | |  |  |
| Fiji | | 59.23115452 | 77.65091268 | | 64.45536092 | |  |  |
| Iceland | | 57.47309309 | 72.02872346 | | 64.82587702 | |  |  |
| Argentina | | 57.39822783 | 68.49802524 | | 66.6987251 | |  |  |
| Jamaica | | 57.13213164 | 73.80857747 | | 71.47127711 | |  |  |
| Northern Mariana Islands | | 53.86137739 | 75.25238845 | | 58.77897618 | |  |  |
| Saint Vincent and the Grenadines | | 53.24806403 | 77.32606689 | | 67.94492752 | |  |  |
| Canada | | 52.89374413 | 72.42665937 | | 64.14320854 | |  |  |
| Barbados | | 52.4584962 | 67.71484494 | | 58.44235293 | |  |  |
| Malta | | 51.05423727 | 66.9292692 | | 60.95806278 | |  |  |
| American Samoa | | 50.69850063 | 66.40840427 | | 54.24809275 | |  |  |
| Samoa | | 48.40553642 | 60.14958124 | | 52.09627386 | |  |  |
| Tuvalu | | 46.81851981 | 56.36065717 | | 50.12929123 | |  |  |
| Cook Islands | | 46.70300888 | 65.28355513 | | 54.35267132 | |  |  |
| USA | | 45.73418476 | 68.45669529 | | 65.5958095 | |  |  |
| Sweden | | 45.61600592 | 42.49755081 | | 42.29575328 | |  |  |
| Bermuda | | 41.8737194 | 61.63428824 | | 50.17793029 | |  |  |
| Macedonia | | 40.90064396 | 53.92158441 | | 47.56433161 | |  |  |
| Micronesia (Federated States of) | | 38.83399747 | 50.99817528 | | 42.41283641 | |  |  |
| Switzerland | | 37.25839675 | 48.82688144 | | 44.12371508 | |  |  |
| Cuba | | 36.3742519 | 62.69104576 | | 54.81380612 | |  |  |
| Norway | | 34.53667609 | 42.88003471 | | 35.65245084 | |  |  |
| Lesotho | | 34.36323765 | 41.31700103 | | 37.56091885 | |  |  |
| Kazakhstan | | 34.2180601 | 44.91492467 | | 41.41110505 | |  |  |
| Netherlands | | 34.01507819 | 42.62659891 | | 34.78467466 | |  |  |
| UK | | 32.49798684 | 35.200886 | | 32.68396144 | |  |  |
| France | | 32.01219968 | 42.96180317 | | 38.69075096 | |  |  |
| Spain | | 31.07475251 | 46.18861335 | | 29.88032467 | |  |  |
| Uruguay | | 29.78649257 | 35.80452329 | | 36.79783442 | |  |  |
| Puerto Rico | | 29.41677385 | 49.27338879 | | 40.10354093 | |  |  |
| Slovenia | | 29.05381374 | 42.16545018 | | 34.36862606 | |  |  |
| Guyana | | 28.19621068 | 45.40844282 | | 37.41619812 | |  |  |
| Portugal | | 27.82597894 | 44.90190599 | | 41.88646342 | |  |  |
| Tonga | | 27.65773851 | 37.5221592 | | 31.93821386 | |  |  |
| Monaco | | 26.33709626 | 31.71790652 | | 27.74636251 | |  |  |
| Finland | | 25.82533727 | 39.56080389 | | 29.79101079 | |  |  |
| Slovakia | | 25.74593207 | 36.41513585 | | 30.16068601 | |  |  |
| Austria | | 25.64794141 | 37.9709965 | | 30.56544296 | |  |  |
| Poland | | 24.49285057 | 35.53887632 | 26.23718058 | | | | |
| Belgium | | 24.29567418 | 32.50877593 | 26.9024123 | | | | |
| Italy | | 23.42150788 | 34.70190448 | 31.74841271 | | | | |
| Dominica | | 23.27027407 | 38.08164003 | 30.79873766 | | | | |
| Albania | | 23.19493615 | 41.99758589 | 34.76912502 | | | | |
| Montenegro | | 22.69519364 | 32.92827964 | 28.65968526 | | | | |
| Nauru | | 22.56841906 | 27.80780237 | 26.71973267 | | | | |
| United States Virgin Islands | | 22.17759592 | 46.05649754 | 32.44738317 | | | | |
| Armenia | | 21.03250385 | 39.24913629 | 32.72012935 | | | | |
| Greenland | | 20.92878941 | 37.75855213 | 32.61751227 | | | | |
| Czech Republic | | 20.14758001 | 30.41279796 | 26.67908426 | | | | |
| Japan | | 17.30448861 | 37.48063649 | 30.87267983 | | | | |
| Greece | | 15.99378311 | 28.35210492 | 27.17677466 | | | | |
| Germany | | 15.97624892 | 25.04521939 | 20.00838643 | | | | |
| Denmark | | 14.41907603 | 22.74871585 | 19.39082609 | | | | |
| Russia | | 11.7175031 | 18.80084204 | 15.84028853 | | | | |
| Serbia | | 11.17445252 | 21.23561718 | 16.05074656 | | | | |
| Moldova | | 7.991454729 | 21.10500078 | 16.37229727 | | | | |
| Belarus | | 7.779295256 | 16.01739347 | 12.13734986 | | | | |
| Tokelau | | 6.965834438 | 15.10312746 | 13.19859517 | | | | |
| Hungary | | 6.133861154 | 15.0291134 | 13.55584501 | | | | |
| Croatia | | 4.032528952 | 14.36205136 | 8.746842058 | | | | |
| Estonia | | 1.377959116 | 12.24367155 | 9.635075438 | | | | |
| Bosnia and Herzegovina | | 0.774325165 | 14.95118675 | 9.417268454 | | | | |
| Romania | | -2.379260714 | 6.672869882 | 2.930619605 | | | | |
| Ukraine | | -5.1348601 | 0.252324196 | -3.250959638 | | | | |
| Lithuania | | -5.317940827 | 5.975236954 | 2.714340815 | | | | |
| Niue | | -6.240730829 | 1.025752192 | -1.365432209 | | | | |
| Bulgaria | | -9.155155979 | -1.664064095 | -6.61048055 | | | | |
| Latvia | | -14.09945453 | -4.669627401 | -6.682337878 | | | | |
| Georgia | | -21.83311281 | -16.22056289 | -20.91834999 | | | | |

The number above 0 indicates an increase, while below 0 indicates a decrease. LBP – low back pain, DALYs – disability-adjusted life years.
