# Supplementary material for: Epidemiological trends and geographic disparities in low back pain burden based on the 2021 GBD study: A cross-sectional analysis
Source: Medicine (Baltimore). 2026 Jun 12;105(24):e49201. doi: 10.1097/MD.0000000000049201 (PMC13268564; doi:10.1097/MD.0000000000049201)

Figure S2. The cluster dendrogram based on EAPC age-standardized prevalence, incidence, and DALYs rates of LBP in all GBD regions.

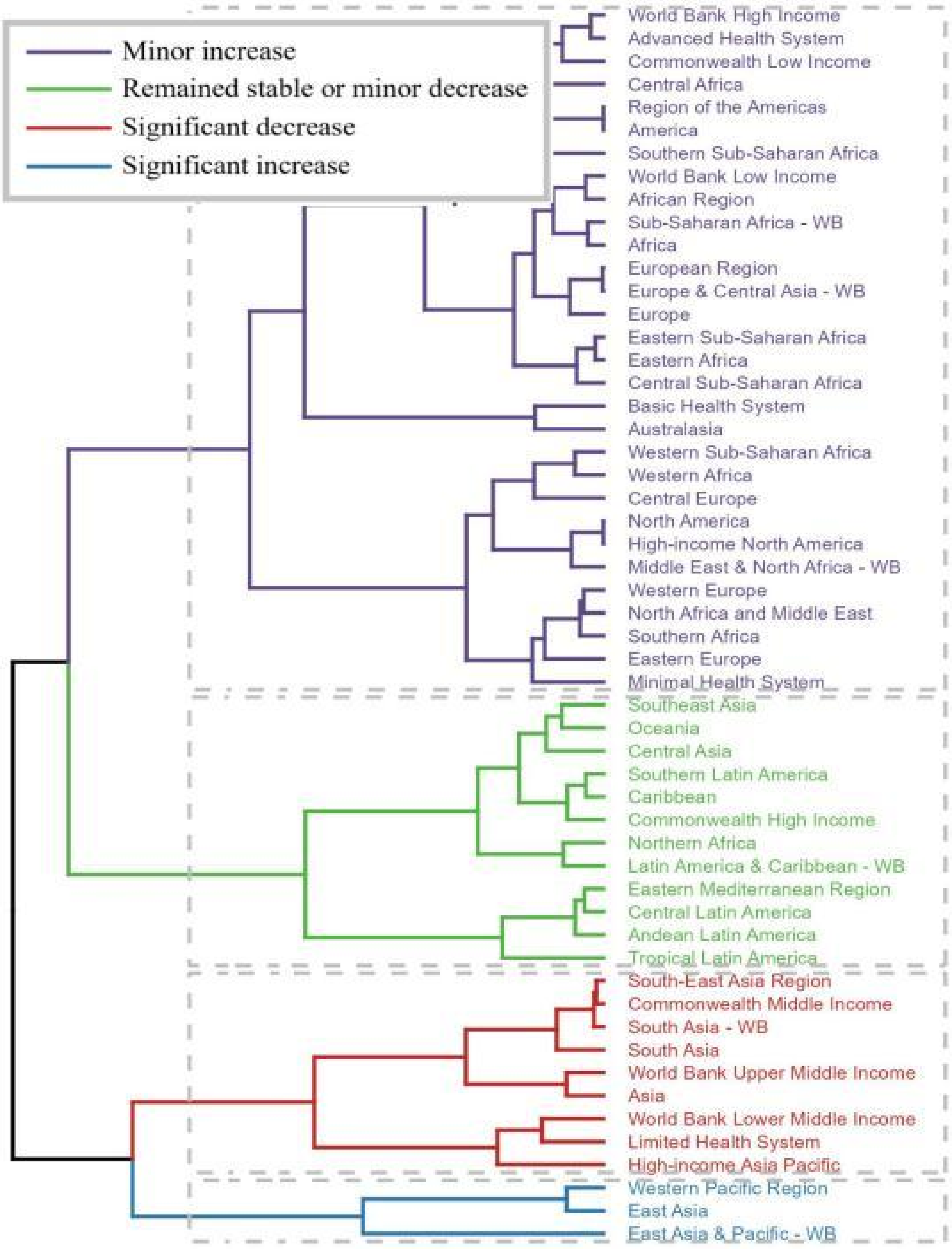

Supplement: Supplementary file 6 [file medi-105-e49201-s006.pdf]
