# Supplementary material for: Epidemiological trends and geographic disparities in low back pain burden based on the 2021 GBD study: A cross-sectional analysis
Source: Medicine (Baltimore). 2026 Jun 12;105(24):e49201. doi: 10.1097/MD.0000000000049201 (PMC13268564; doi:10.1097/MD.0000000000049201)

Figure S3. Age-specific patterns of the global LBP burden, 1990–2021. (A) Trends in age-standardized incidence, prevalence, and DALYs rates across age groups. (B) Trends in the absolute number of cases across age groups.

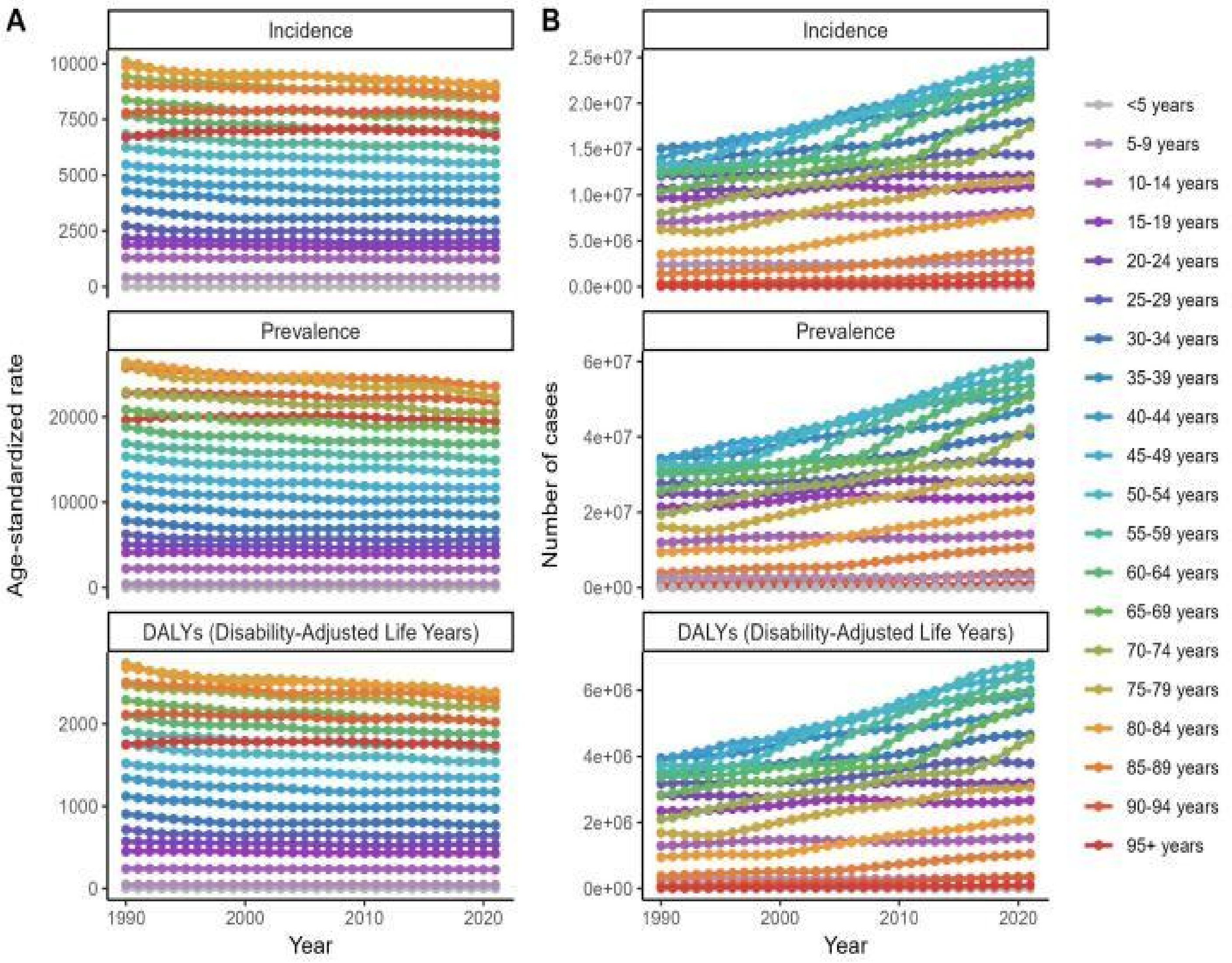

Supplement: Supplementary file 7 [file medi-105-e49201-s007.pdf]
