# Supplementary material for: Epidemiological trends and geographic disparities in low back pain burden based on the 2021 GBD study: A cross-sectional analysis
Source: Medicine (Baltimore). 2026 Jun 12;105(24):e49201. doi: 10.1097/MD.0000000000049201 (PMC13268564; doi:10.1097/MD.0000000000049201)

Figure S4. Global burden of LBP by sex, 2021. (A) Global age-standardized rates of incidence, prevalence, and DALYs per 100,000 population; (B) Global number of cases for incidence, prevalence, and DALYs estimates.

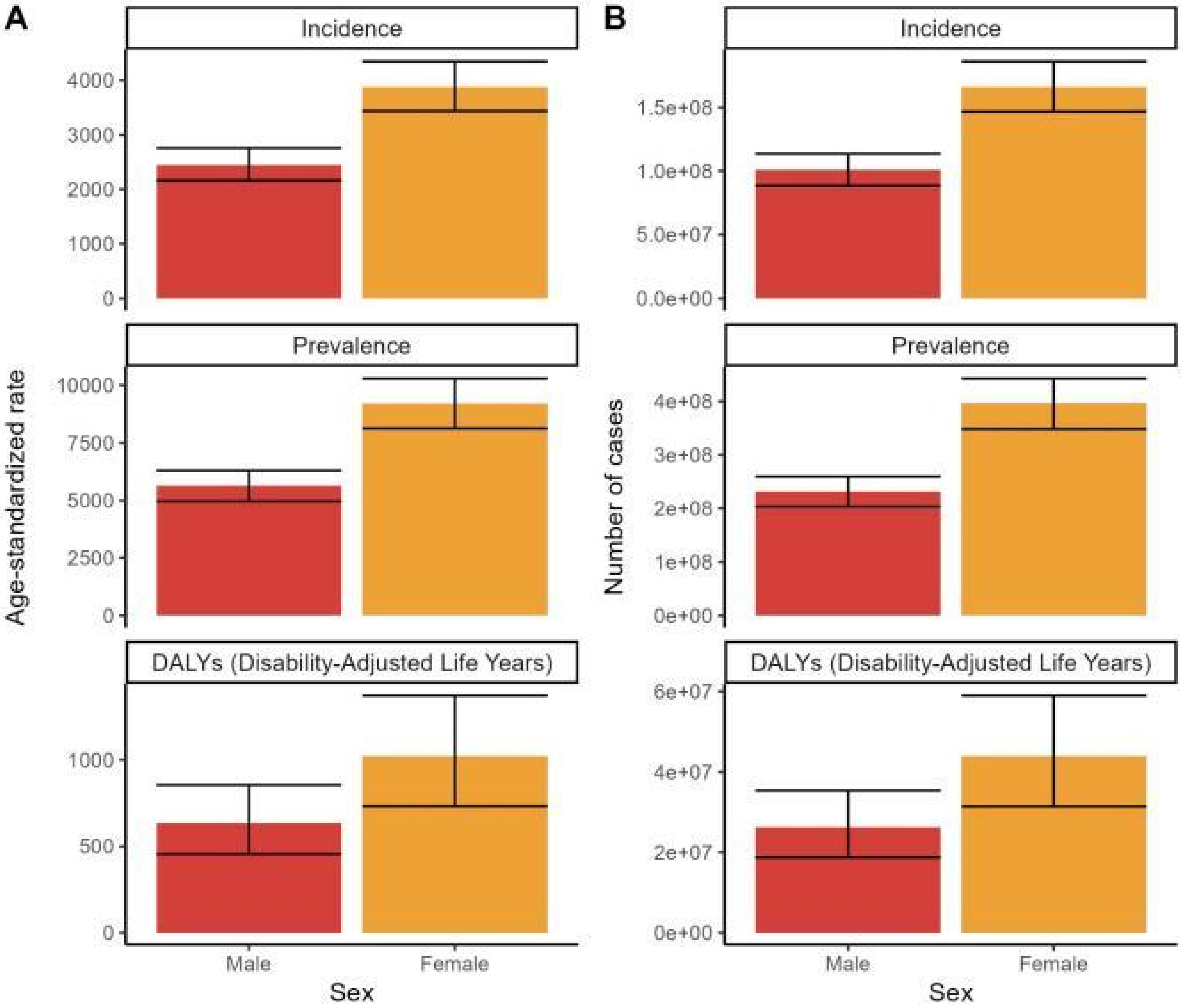

Supplement: Supplementary file 8 [file medi-105-e49201-s008.pdf]
