# Supplementary material for: Epidemiological trends and geographic disparities in low back pain burden based on the 2021 GBD study: A cross-sectional analysis
Source: Medicine (Baltimore). 2026 Jun 12;105(24):e49201. doi: 10.1097/MD.0000000000049201 (PMC13268564; doi:10.1097/MD.0000000000049201)

Figure S5. Global temporal trends of LBP burden by sex, 1990-2021. (A) Changes in the age-standardized rates of incidence, prevalence, and DALYs by sex; (B) Changes in the number of cases for incidence, prevalence, and DALYs by sex.

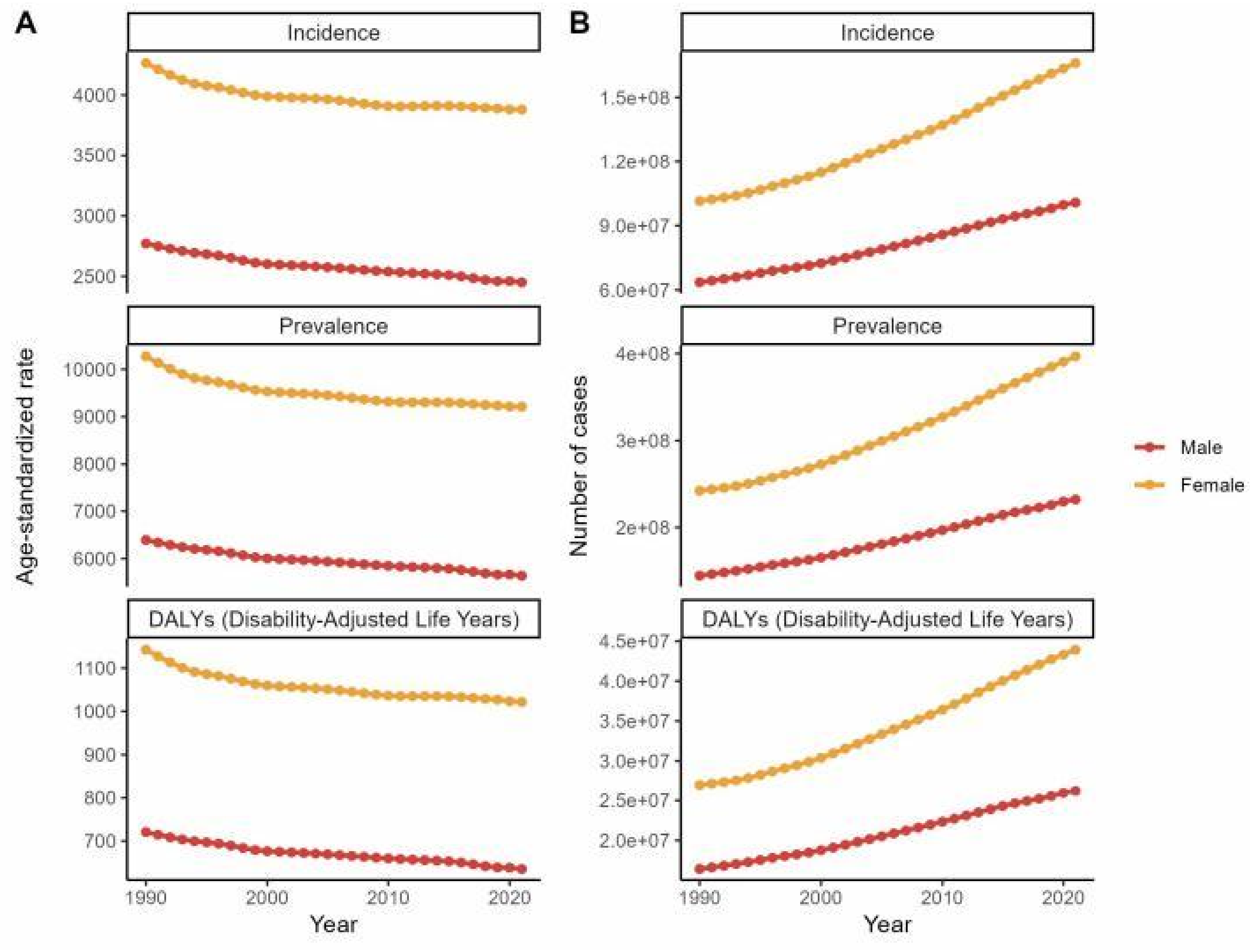

Supplement: Supplementary file 9 [file medi-105-e49201-s009.pdf]
