# Supplementary material for: Epidemiological trends and geographic disparities in low back pain burden based on the 2021 GBD study: A cross-sectional analysis
Source: Medicine (Baltimore). 2026 Jun 12;105(24):e49201. doi: 10.1097/MD.0000000000049201 (PMC13268564; doi:10.1097/MD.0000000000049201)

Figure S7. Temporal trends in the LBP burden across SDI regions, 1990–2021. Trends in age-standardized incidence, prevalence, and DALYs rates. (B) Trends in the absolute number of cases.

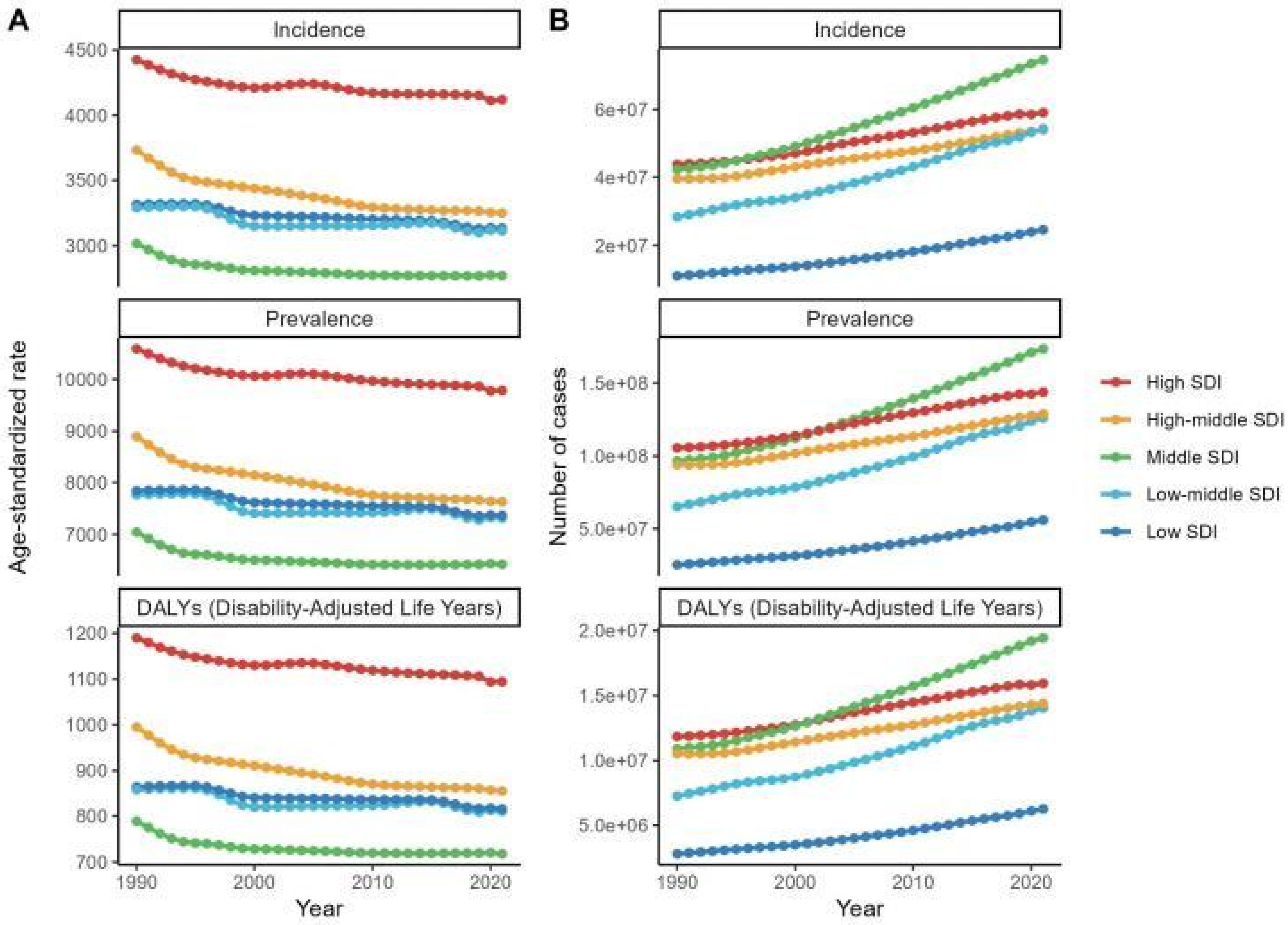

Supplement: Supplementary file 11 [file medi-105-e49201-s011.pdf]
