# Supplementary material for: Epidemiological trends and geographic disparities in low back pain burden based on the 2021 GBD study: A cross-sectional analysis
Source: Medicine (Baltimore). 2026 Jun 12;105(24):e49201. doi: 10.1097/MD.0000000000049201 (PMC13268564; doi:10.1097/MD.0000000000049201)
Supplement: Supplementary file 12 [file medi-105-e49201-s012.docx]

Table S8. Changes in the number of incidence, prevalence, and DALYs cases based on the population-level determinants and causes between 1990 and 2021.

| Location | | Overll difference* | Change due to population-level determinants (% contribute to the total changes) | | |
| --- | --- | --- | --- | --- | --- |
|  |  |  | Aging† | Population# | Epidemiological change& |
| Incidence numbers | Global | 208727331.9 | 39418483.93 (18.89%) | 225721667.7 (108.14%) | -56412819.78 (-27.03%) |
|  | Low SDI | 23997125.08 | -367147.419 (-1.53%) | 26683365.59 (111.19%) | -2319093.092 (-9.66%) |
|  | Low-middle SDI | 48506105.37 | 5144472.853 (10.61%) | 48495817.95 (99.98%) | -5134185.441 (-10.58%) |
|  | Middle SDI | 67565681.87 | 15745282.5 (23.30%) | 63466362.1 (93.93%) | -11645962.73 (-17.24%) |
|  | High-middle SDI | 36640393.89 | 12841408.18 (35.05%) | 43047615.43 (117.49%) | -19248629.72 (-52.53%) |
|  | High SDI | 32065448.53 | 8737273.421 (27.25%) | 31416292.66 (97.98%) | -8088117.552 (-24.72%) |
| Prevalence numbers | Global | 87521904.48 | 15125109.44 (17.28%) | 94029017.09 (107.43%) | -21632222.04 (-24.72%) |
|  | Low SDI | 10246510.74 | -150701.974 (-1.47%) | 11282680 (110.11%) | -885467.282 (-8.64%) |
|  | Low-middle SDI | 20355456.94 | 2007936.799 (9.86%) | 20370894.9 (100.08%) | -2023374.752 (-9.94%) |
|  | Middle SDI | 28529042.71 | 6275005.901 (22.00%) | 26916843.35 (94.35%) | -4662806.54 (-16.34%) |
|  | High-middle SDI | 15353765.26 | 4860578.569 (31.66%) | 17835738.73 (116.17%) | -7342552.044 (-47.82%) |
|  | High SDI | 13012743.75 | 3174909.502 (24.40%) | 12771320.47 (98.14%) | -2933486.222 (-22.54%) |
| DALYs  numbers | Global | 23111304.83 | 4205356.043 (18.20%) | 25317158.25 (109.54%) | -6411209.468 (-27.74%) |
|  | Low SDI | 2702645.71 | -42110.808 (-1.56%) | 2984160.697 (110.42%) | -239404.178 (-8.86%) |
|  | Low-middle SDI | 5401447.32 | 539379.094 (9.99%) | 5419160.055 (100.33%) | -557091.829 (-10.31%) |
|  | Middle SDI | 7529683.29 | 1709407.565 (22.70%) | 7161459.561 (95.11%) | -1341183.836 (-17.81%) |
|  | High-middle SDI | 4061113.99 | 1368915.777 (33.71%) | 4830659.863 (118.95%) | -2138461.647 (-52.66%) |
|  | High SDI | 3432659.14 | 891531.083 (25.97%) | 3508876.711 (102.22%) | -967748.651 (-28.19%) |

*Change in number between year 2021 and 1990.

†Change in deaths number due to change in the age structure. #Change in deaths number due to change in population number.

& Change in deaths number due to epidemiological changes. Epidemiological changes refer to the deaths number change when age structure and population hold constant.

Abbreviations: DALYs ，disability-adjusted life-years; SDI, Socio-Demographic Index.
