# Supplementary material for: Epidemiological trends and geographic disparities in low back pain burden based on the 2021 GBD study: A cross-sectional analysis
Source: Medicine (Baltimore). 2026 Jun 12;105(24):e49201. doi: 10.1097/MD.0000000000049201 (PMC13268564; doi:10.1097/MD.0000000000049201)
Supplement: Supplementary file 13 [file medi-105-e49201-s013.docx]

Table S2. The incident cases and ASR for LBP in all GBD regions between 1990 and 2021, and its temporal trends.

| **Location** | **1990** | **2021** | **EAPC (95% CI)** |
| --- | --- | --- | --- |
|  | **ASR per 100 000** | **ASR per 100 000** |  |
|  | **Cases (95% UI)** | **Cases (95% UI)** | **1990-2021** |
|  | **(95% UI)** | **(95% UI)** |  |

Global

165063882

(145785270-185933884)

3534.99

(3133.04-3960.99)

266873321

(235369489-299406380)

3176.63

(2811.82-3562.29)

-0.29 (-0.32 to -0.26)

GBD region

|  | 66703462 | 4525.63 | 85505224 | 4278.63 |
| --- | --- | --- | --- | --- |
| Advanced Health System |  |  |  | -0.13 (-0.15 to -0.1) |
|  | (59627450-74646118) | (4041.75-5073.09) | (76107959-94836959) | (3823.88-4775.03) |
|  | 13993215 | 3290.96 | 32026145 | 3198.27 |
| Africa |  |  |  | -0.1 (-0.12 to -0.08) |
|  | (12309228-15785197) | (2906.15-3699.13) | (28183215-36306581) | (2818.84-3596.03) |
|  | 10614034 | 3200.34 | 24844405 | 3103.52 |
| African Region |  |  |  | -0.11 (-0.13 to -0.09) |
|  | (9282486-11966178) | (2822.19-3600.31) | (21828663-28118858) | (2732.11-3491.05) |
|  | 26986725 | 4015.37 | 44495353 | 3805.02 |
| America |  |  |  | -0.11 (-0.15 to -0.08) |
|  | (23897484-30567007) | (3569.25-4527.65) 2520.72 | (39961888-49505993) | (3412.41-4260.1) 2537.33 |
| Andean Latin America | 736141 (647842-830131) |  | 1646434 (1458880-1844797) | 0.04 (0.01-0.06) |
|  |  | (2220.84-2846.49) |  | (2248.54-2847.88) |
|  | 82926603 | 3153.77 | 141407502 | 2805.88 |
| Asia |  |  |  | -0.3 (-0.35 to -0.24) |
|  | (72772529-94041106) | (2786.11-3541.63) 5066.6 | (123926344-158972438) | (2474.38-3155.81) 4715.64 |
| Australasia | 1116813 (995033-1264046) |  | 1793375 (1584866-1997436) | -0.16 (-0.18 to -0.13) |
|  |  | (4532.9-5758.99) |  | (4181.41-5288.91) |
|  | 58573529 | 3027.11 | 99594950 | 2784.69 |
| Basic Health System |  |  |  | -0.16 (-0.21 to -0.11) |
|  | (51307840-66471444) | (2670.21-3407.85) 2649.01 | (87173447-112270884) | (2453.84-3128.72) 2618.94 |
| Caribbean | 823557 (721753-927447) |  | 1338623 (1184999-1505225) | -0.02 (-0.03 to -0.01) |
|  |  | (2327.25-2988.32) 3340.4 |  | (2316.54-2943.07) 3210.93 |
| Central Africa | 1428733 (1247200-1623279) |  | 3588181 (3162400-4099888) | -0.15 (-0.17 to -0.12) |
|  |  | (2931.38-3763.14) 3946.39 |  | (2836.31-3622.49) 3908.43 |
| Central Asia | 2219225 (1967968-2496734) |  | 3627338 (3190645-4122873) | -0.03 (-0.03 to -0.02) |
|  |  | (3493.46-4422.79) 5279.12 |  | (3443.95-4408.07) 5181.24 |
| Central Europe | 7329411 (6501413-8246292) |  | 8079891 (7097942-9076612) | -0.07 (-0.08 to -0.07) |
|  |  | (4690.7-5941.73) 3140.4 |  | (4595.33-5834.52) 3173.31 |
| Central Latin America | 4022190 (3510176-4572563) |  | 8328520 (7336429-9409867) | 0.03 (-0.01-0.07) |
|  |  | (2773.02-3538.98) 3359.32 |  | (2801.57-3585.76) 3259.03 |
| Central Sub-Saharan Africa | 1181032 (1029213-1339383) |  | 2999484 (2638593-3421283) | -0.12 (-0.14 to -0.09) |
|  |  | (2938.81-3787.45) |  | (2879.66-3674.29) |
| Commonwealth High |  | 4215.91 |  | 4078.92 |
|  | 5315670 (4726730-5994373) |  | 7646802 (6730958-8574558) | 0 (-0.03-0.03) |
| Income |  | (3754.79-4788.66) |  | (3606.52-4592.35) |
|  |  | 3647.1 | 10801188 | 3479.74 |
| Commonwealth Low Income | 4949319 (4383372-5586090) |  |  | -0.13 (-0.16 to -0.1) |
|  |  | (3244.54-4114.67) | (9481037-12214449) | (3058.75-3911.01) |
| Commonwealth Middle | 27699847 | 3153.87 | 54469234 | 2866.06  -0.32 (-0.4 to -0.23) |
| Income | (24234404-31351837) | (2778.96-3538.78) | (47730335-61727710) | (2519.6-3224.88) |

East Asia 31011647 2866.16 45417237 2369.25 -0.44 (-0.53 to -0.35)

|  | (27123453-35291824) | (2520.47-3229.74) | (39382941-51288188) | (2088.98-2663.65) |
| --- | --- | --- | --- | --- |
|  | 50005779 | 3024.37 | 76068719 | 2589.46 |
| East Asia & Pacific - WB |  |  |  | -0.38 (-0.44 to -0.32) |
|  | (43860552-56712908) | (2669.71-3401.76) 3394.82 | (66531860-85827291) | (2284.68-2911.05) 3278.46 |
| Eastern Africa | 3725194 (3265893-4201417) |  | 8702891 (7638819-9853398) | -0.12 (-0.13 to -0.11) |
|  |  | (2993.08-3817.08) |  | (2892.8-3691.2) |
|  | 12246259 | 4752.91 | 12839596 | 4619.12 |
| Eastern Europe |  |  |  | -0.04 (-0.06 to -0.03) |
|  | (10800139-13742205) | (4216.92-5309.17) | (11238456-14387501) | (4112.22-5173.33) |
| Eastern Mediterranean |  | 3543.85 | 23121500 | 3552.57 |
|  | 9663509 (8436458-10928156) |  |  | 0.03 (0.02-0.04) |
| Region |  | (3123.36-3992.44) 3349.44 | (20189207-26479107) | (3124.47-4001.79) 3238.98 |
| Eastern Sub-Saharan Africa | 3920767 (3442324-4415532) |  | 9141988 (8019263-10354866) | -0.12 (-0.13 to -0.11) |
|  |  | (2952.44-3770.07) |  | (2855.2-3641.36) |
|  | 40861541 | 4473.92 | 48559042 | 4306.38 |
| Europe |  |  |  | -0.09 (-0.1 to -0.07) |
|  | (36376822-45610278) | (3988.1-5012.07) | (42783860-54371752) | (3814.69-4841.36) |
|  | 42317215 | 4442.75 | 51139920 | 4266.54 |
| Europe & Central Asia - WB |  |  |  | -0.1 (-0.11 to -0.08) |
|  | (37669303-47266011) | (3957.97-4976.85) | (45063369-57241207) | (3781.65-4793.85) |
|  | 42636542 | 4443.05 | 51720385 | 4267.07 |
| European Region |  |  |  | -0.1 (-0.11 to -0.08) |
|  | (37952922-47628996) | (3958.36-4977.67) 4629.55 | (45568354-57890555) 11053079 | (3781.73-4794.32) 4236.84 |
| High-income Asia Pacific | 9006130 (7938409-10123637) |  |  | -0.23 (-0.25 to -0.21) |
|  |  | (4106.23-5216.54) | (9710908-12353921) | (3745.6-4769.29) |
|  | 14670611 | 4712.08 | 20525968 | 4430.69 |
| High-income North America |  |  |  | -0.07 (-0.12 to -0.01) |
|  | (13042429-16545457) | (4197.89-5302.41) | (18564440-22433456) | (4019.85-4856.34) |
| Latin America & Caribbean - | 12416754 | 3420.02 | 24095751 | 3421.12  0 (-0.02-0.01) |
| WB | (10897217-14100161) | (3019.8-3856.04) | (21241565-27122539) | (3019.81-3845.58) |
|  | 36747934 | 3237.04 | 74637023 | 2991.76 |
| Limited Health System |  |  |  | -0.26 (-0.32 to -0.2) |
|  | (32228711-41581233) | (2856.88-3630.79) | (65349626-84463576) | (2633.45-3361.69) |
| Middle East & North Africa - |  | 3780.02 | 16572662 | 3688.95 |
|  | 7269146 (6366554-8198195) |  |  | -0.07 (-0.08 to -0.06) |
| WB |  | (3352-4272.31) 3246.87 | (14552595-18919008) | (3264.77-4160.03) 3193.41 |
| Minimal Health System | 2848224 (2491327-3217337) |  | 6884819 (6049441-7840809) | -0.07 (-0.09 to -0.04) |
|  |  | (2857.57-3653.26) |  | (2810.65-3595.49) |
|  |  | 3762.42 | 21554211 | 3686.08 |
| North Africa and Middle East | 9687730 (8533318-10892164) |  |  | -0.05 (-0.06 to -0.04) |
|  |  | (3358.17-4228.9) | (18949089-24589228) | (3259.35-4157.55) |
|  | 14669927 | 4711.78 | 20525655 | 4430.48 |
| North America |  |  |  | -0.07 (-0.12 to -0.01) |
|  | (13041840-16544602) | (4197.62-5302.05) 3665.16 | (18564074-22433116) | (4019.64-4856.1) 3680.05 |
| Northern Africa | 3412975 (3001362-3864029) |  | 7117171 (6249214-8070427) | 0.01 (-0.02-0.04) |
|  |  | (3252.26-4148.07) 2756.62 |  | (3244.95-4151.16) 2728.63 |
| Oceania | 126038 (109329-143469) |  | 297549 (259577-339195) | -0.02 (-0.03-0) |
|  |  | (2423.35-3122.59) |  | (2394.39-3098.38) |
|  | 26986725 | 4015.37 | 44495353 | 3805.02 |
| Region of the Americas |  |  |  | -0.11 (-0.15 to -0.08) |
|  | (23897484-30567007) | (3569.25-4527.65) | (39961888-49505993) | (3412.41-4260.1) |
|  | 31051394 | 3121.41 | 57946645 | 2829.66 |
| South-East Asia Region |  |  |  | -0.32 (-0.39 to -0.25) |
|  | (27239957-35113506) | (2755.38-3500.96) | (50669376-65547747) | (2489.96-3179.04) |
|  | 26843747 | 3285.69 | 51725612 | 2970.33 |
| South Asia |  |  |  | -0.33 (-0.42 to -0.24) |
|  | (23552246-30395436) | (2895.16-3689.97) | (45244519-58532118) | (2606.51-3334.99) |

South Asia - WB 27485602 3273.87 53118922 2971.86 -0.31 (-0.4 to -0.23)

|  | (24117131-31115340) | (2885.36-3675.22) 2563.77 | (46486979-60110262) 18102798 | (2608.27-3336.34) 2518.34 |
| --- | --- | --- | --- | --- |
| Southeast Asia | 9092801 (7980280-10181926) |  |  | -0.04 (-0.05 to -0.03) |
|  |  | (2263.65-2880.72) 3076.37 | (15777830-20544107) | (2218.52-2844.11) 3011.18 |
| Southern Africa | 1935133 (1705715-2175077) |  | 4106293 (3603037-4643098) | -0.06 (-0.07 to -0.06) |
|  |  | (2716.9-3459.71) 4169.07 |  | (2646.67-3380.1) 4138.35 |
| Southern Latin America | 2000972 (1776610-2262609) |  | 3175959 (2802991-3549723) | -0.01 (-0.05-0.03) |
|  |  | (3705.78-4713.46) |  | (3660.72-4648.24) |
| Southern Sub-Saharan |  | 2946.99 |  | 2810.63 |
|  | 1085155 (954299-1220274) |  | 2000245 (1750711-2271266) | -0.13 (-0.14 to -0.12) |
| Africa |  | (2592.52-3308.26) |  | (2465.1-3178.51) |
|  | 10610624 | 3197.54 | 24964674 | 3108.27 |
| Sub-Saharan Africa - WB |  |  |  | -0.1 (-0.12 to -0.08) |
|  | (9281738-11965274) | (2820.1-3594.1) 3780.7 | (21942542-28294700) | (2737.11-3497.12) 3857.44 |
| Tropical Latin America | 4863631 (4256075-5541750) |  | 9654115 (8506642-10873405) | 0.04 (0.02-0.06) |
|  |  | (3331.84-4274.24) 3035.83 |  | (3402.33-4336.37) 2962.37 |
| Western Africa | 3491180 (3058318-3939244) |  | 8511610 (7446860-9653360) | -0.09 (-0.13 to -0.06) |
|  |  | (2675.21-3418.24) |  | (2603.97-3337.87) |
|  | 19199804 | 4173.12 | 23987309 | 4040.66 |
| Western Europe |  |  |  | -0.06 (-0.07 to -0.04) |
|  | (17127530-21392148) | (3732.89-4684.76) | (21198828-26893840) | (3579.55-4541.58) |
|  | 43232710 | 3098.49 | 63234318 | 2591.59 |
| Western Pacific Region |  |  |  | -0.44 (-0.51 to -0.37) |
|  | (37895548-49104603) | (2729.53-3488.08) | (55219554-71488286) | (2280.61-2916.55) |
| Western Sub-Saharan |  | 3054.43 |  | 2974.28 |
|  | 3880221 (3401179-4375940) |  | 9583990 (8386342-10870298) | -0.1 (-0.14 to -0.06) |
| Africa |  | (2693.49-3440.05) |  | (2616.26-3351.94) |
|  | 52021295 | 4484.26 | 68697508 | 4237.04 |
| World Bank High Income |  |  |  | -0.12 (-0.15 to -0.1) |
|  | (46567745-58238281) | (4004.44-5040.99) 3327.45 | (61403863-75945581) 15471980 | (3793.88-4728.05) 3232.51 |
| World Bank Low Income | 6796997 (5953308-7682386) |  |  | -0.11 (-0.13 to -0.09) |
|  |  | (2935.44-3742.89) | (13575206-17519391) | (2851.06-3634.65) |
| World Bank Lower Middle | 48854256 | 3283.57 | 94846566 | 3018.39  -0.27 (-0.32 to -0.22) |
| Income | (42869479-55196788) | (2902.71-3680) | (83232510-107441735) | (2660.83-3392.97) |
| World Bank Upper Middle | 57200072 | 3235.9 | 87605259 | 2873.19  -0.28 (-0.34 to -0.22) |
| Income | (50315021-64738166) | (2862.33-3636.48) | (76602973-98959395) | (2530.95-3227.23) |

ASR, age-standardized rate; LBP, low back pain; UI, uncertainty interval, CI, confdence interval; EAPC, estimated annual percentage change; DALYs, disability-adjusted life years.
