# Supplementary material for: Epidemiological trends and geographic disparities in low back pain burden based on the 2021 GBD study: A cross-sectional analysis
Source: Medicine (Baltimore). 2026 Jun 12;105(24):e49201. doi: 10.1097/MD.0000000000049201 (PMC13268564; doi:10.1097/MD.0000000000049201)
Supplement: Supplementary file 14 [file medi-105-e49201-s014.docx]

Table S3. The DALYs cases and ASR for LBP in all GBD regions between 1990 and 2021, and its temporal trends.

| **Location** | **1990** |  | **2021** | **EAPC (95% CI) 1990-2021** |
| --- | --- | --- | --- | --- |
|  | **Cases (95% UI) ASR per 100 000 (95% UI)** | **Cases (95% UI)** | **ASR per 100 000 (95% UI)** |  |

**Global**

43386226

(31083937-58355210)

937.34 (669.13-1261)

70156962

(50194205-94104688)

832.18 (595.85-1115.24)

-0.32

(-0.35 to -0.28)

**GBD region**

| Advanced Health System | 18086354  (12971692-24355350) | 1217.84 (874.78-1638.71) | 23117190  (16648457-30897629) | 1139.02 (822.46-1523.91) | -0.15  (-0.17 to -0.13) |
| --- | --- | --- | --- | --- | --- |
| Africa | 3550688 (2526880-4728418) | 856.51 (611.15-1148.64) | 8175006  (5817444-10930127) | 831.91 (595.29-1120.63) | -0.09  (-0.11 to -0.06) |
| African Region | 2671224 (1904967-3570397) | 827.7 (590.89-1109.89) | 6293266  (4480897-8431960) | 803.97 (574.08-1081.66) | -0.09  (-0.11 to -0.07) |
| America | 7134208 (5113140-9545637) | 1068.79 (765.12-1430.1) | 11790899  (8562848-15646692) | 1001.33 (726.14-1327.54) | -0.13 (-0.17 to -0.1) |
| Andean Latin America | 185029 (130897-246432) | 646.13 (458.75-865.08) | 419827 (298545-559565) | 646.88 (458.19-863.75) | 0.03 (0.01-0.06) |
| Asia | 21517468  (15382219-28868689) | 827.05 (589.39-1114.06) | 36875193  (26280962-49670898) | 727.55 (519.4-976.38) | -0.32  (-0.37 to -0.26) |
| Australasia | 304861 (215769-408899) | 1376.56 (976-1844.16) | 488387 (349002-661173) | 1268.22 (904.77-1709.01) | -0.19  (-0.22 to -0.17) |
| Basic Health System | 15202315  (10857338-20406313) | 793.96 (564.6-1070.26) | 26149537  (18636239-35267422) | 725 (518.71-971.86) | -0.17  (-0.23 to -0.11) |
| Caribbean | 210760 (151733-281762) | 684.15 (489.1-922.21) | 344542 (247890-462391) | 670.86 (483.43-901.16) | -0.02  (-0.03 to -0.01) |
| Central Africa | 359672 (255082-483127) | 865.9 (622.54-1172.16) | 908408 (645873-1210115) | 835.59 (596.52-1133.95) | -0.13 (-0.15 to -0.1) |
| Central Asia | 576974 (415109-767337) | 1043.07 (747.31-1393.92) | 952839 (682011-1286067) | 1029.87 (732.88-1385.72) | -0.02  (-0.03 to -0.02) |
| Central Europe | 2060141 (1476137-2773949) | 1475.91 (1058.6-1976.09) | 2290522  (1634308-3088294) | 1439.39 (1027.23-1934.4) | -0.08  (-0.09 to -0.08) |
| Central Latin America | 1038273 (731753-1387394) | 827.1 (589.11-1110.66) | 2204585  (1574612-2963661) | 837.42 (597.9-1127.03) | 0.04 (0-0.09) |
| Central Sub-Saharan Africa | 295144 (209057-395657) | 863.94 (623.33-1164.32) | 754976 (536134-1006808) | 842.65 (603.12-1136.37) | -0.09  (-0.12 to -0.07) |
| Commonwealth High Income | 1416850 (1008473-1902164) | 1114.83 (794.86-1493.6) | 2036471  (1455052-2752858) | 1072.35 (765.81-1439.32) | -0.02  (-0.05-0.01) |
| Commonwealth Low Income | 1294836 (919907-1724615) | 988.85 (713.6-1325.63) | 2843093  (2030925-3815817) | 932.35 (669.83-1256.74) | -0.14 (-0.19 to -0.1) |
| Commonwealth Middle Income | 6976875 (4995453-9325904) | 806.48 (579.48-1083.29) | 13809555  (9882320-18602151) | 729.35 (521.29-980.07) | -0.32  (-0.42 to -0.23) |
| East Asia | 8080257 (5742297-10947390) | 751.03 (532.46-1014.91) | 11867556  (8333735-16052098) | 611.77 (433.79-820.52) | -0.46  (-0.56 to -0.36) |
| East Asia & Pacific - WB | 13121930  (9341526-17718411) | 798.63 (567.12-1077.28) | 20064477  (14159089-27078359) | 676 (480.82-907.82) | -0.4 (-0.46 to -0.33) |

| Eastern Africa | 941050 (665220-1253716) | 885.73 (632.25-1188.29) | 2214416  (1572035-2963373) | 855.13 (611.38-1151.75) | -0.11 (-0.11 to -0.1) |
| --- | --- | --- | --- | --- | --- |
| Eastern Europe | 3353744 (2399226-4527209) | 1293.39 (925.73-1741.73) | 3508059  (2503644-4720036) | 1241.42 (889.66-1666.37) | -0.05  (-0.08 to -0.03) |
| Eastern Mediterranean Region | 2477888 (1745950-3321076) | 929.64 (664.18-1249.88) | 6009409  (4268837-8049806) | 927.82 (667.5-1250.19) | 0.03 (0.02-0.05) |
| Eastern Sub-Saharan Africa | 987109 (701131-1317166) | 872.15 (624.42-1168.26) | 2317834  (1643306-3112633) | 844.44 (602.74-1135.48) | -0.1 (-0.11 to -0.1) |
| Europe | 11104843  (7960672-14917367) | 1204.88 (864.97-1620.3) | 13212814  (9368530-17715446) | 1152.36 (825.94-1552.13) | -0.1 (-0.12 to -0.08) |
| Europe & Central Asia - WB | 11480942  (8235544-15429647) | 1195.87 (858.34-1609.14) | 13886540  (9850935-18628918) | 1140.65 (817.25-1536.32) | -0.11 (-0.13 to -0.1) |
| European Region | 11566378  (8296797-15545312) | 1195.92 (858.37-1609.27) | 14043128  (9963614-18841082) | 1140.84 (817.42-1536.72) | -0.11 (-0.13 to -0.1) |
| High-income Asia Pacific | 2466629 (1764723-3339335) | 1256.21 (903.17-1688.97) | 3027728  (2147702-4085674) | 1140.12 (814.58-1534.15) | -0.27  (-0.29 to -0.24) |
| High-income North America | 3943904 (2820966-5255899) | 1260.42 (905.37-1682.35) | 5425099  (3944504-7058790) | 1159.5 (843.77-1513.97) | -0.11  (-0.17 to -0.05) |
| Latin America & Caribbean - WB | 3216226 (2293341-4285359) | 899.92 (642.02-1208.04) | 6398658  (4571112-8603196) | 903.94 (647.35-1213.47) | 0.01 (-0.01-0.03) |
| Limited Health System | 9330671 (6650464-12452952) | 837.68 (599.48-1125.9) | 19086377  (13664928-25669301) | 770.44 (550.28-1034.6) | -0.26 (-0.33 to -0.2) |
| Middle East & North Africa - WB | 1884022 (1325940-2525600) | 1002.74 (715.59-1346.36) | 4358429  (3113375-5840504) | 968.18 (697.67-1298.45) | -0.1 (-0.12 to -0.08) |
| Minimal Health System | 714945 (510981-955440) | 837.85 (601.73-1126.74) | 1735669  (1226435-2312171) | 829.56 (592.6-1121.02) | -0.04  (-0.06 to -0.01) |
| North Africa and Middle East | 2513043 (1774596-3348746) | 997.39 (719.01-1341.15) | 5661667  (4030973-7583353) | 967.37 (696.7-1296.96) | -0.07  (-0.09 to -0.05) |
| North America | 3943736 (2820856-5255684) | 1260.35 (905.31-1682.25) | 5425023  (3944454-7058669) | 1159.45 (843.73-1513.89) | -0.11  (-0.17 to -0.05) |
| Northern Africa | 888699 (625845-1191949) | 972.36 (692.56-1306.39) | 1872581  (1336922-2505119) | 969.67 (700.01-1298.84) | 0 (-0.04-0.05) |
| Oceania | 31920 (22753-42740) | 712.13 (507.26-954.19) | 76195 (54394-102628) | 705.15 (504.09-943.46) | -0.01  (-0.02-0.01) |
| Region of the Americas | 7134208 (5113140-9545637) | 1068.79 (765.12-1430.1) | 11790899  (8562848-15646692) | 1001.33 (726.14-1327.54) | -0.13 (-0.17 to -0.1) |
| South-East Asia Region | 7924792 (5652443-10556828) | 808.25 (579.04-1085.89) | 14937091  (10667763-20107563) | 728.43 (520.16-976.95) | -0.32 (-0.4 to -0.25) |
| South Asia | 6828442 (4869769-9096399) | 849.6 (608.76-1141.44) | 13247628  (9487458-17842552) | 762.34 (544.52-1022.84) | -0.34  (-0.43 to -0.24) |
| South Asia - WB | 6991360 (4986204-9316090) | 846.37 (606.41-1137.06) | 13602052  (9741685-18329761) | 762.67 (544.79-1022.93) | -0.32  (-0.42 to -0.23) |
| Southeast Asia | 2326494 (1659013-3122018) | 665.22 (474.6-892.67) | 4757936  (3371946-6410456) | 657.99 (467.99-885.65) | 0 (-0.01-0.01) |

| Southern Africa | 486999 (349540-647785) | 793.44 (567.83-1060.59) | 1033832  (740825-1382027) | 770.88 (551.2-1034.33) | -0.07  (-0.08 to -0.06) |
| --- | --- | --- | --- | --- | --- |
| Southern Latin America | 524866 (373418-706452) | 1097.59 (782.23-1475.55) | 843277 (596115-1134514) | 1090.83 (772.11-1464.42) | -0.02  (-0.06-0.02) |
| Southern Sub-Saharan Africa | 273764 (196708-366348) | 758.59 (541.31-1017.04) | 505036 (361967-678388) | 712.89 (510.7-955.02) | -0.16  (-0.17 to -0.15) |
| Sub-Saharan Africa - WB | 2669497 (1902983-3563543) | 826.98 (590.52-1108.57) | 6316305  (4486454-8450242) | 805.48 (575.58-1085.3) | -0.08 (-0.1 to -0.06) |
| Tropical Latin America | 1264922 (900250-1690841) | 998.4 (712.23-1339.07) | 2598774  (1862649-3499223) | 1029.57 (741.54-1382.07) | 0.07 (0.05-0.1) |
| Western Africa | 874268 (624304-1171193) | 779.46 (555.63-1046.79) | 2145768  (1520280-2874615) | 766.13 (546.74-1032.3) | -0.06  (-0.09 to -0.02) |
| Western Europe | 5145785 (3678724-6891381) | 1105.46 (797.53-1482.55) | 6444977  (4566679-8666045) | 1068.98 (766.39-1441.34) | -0.07  (-0.08 to -0.05) |
| Western Pacific Region | 11380228  (8090481-15400505) | 819.85 (581.47-1107.3) | 16657289  (11729274-22533833) | 675.49 (480-906.37) | -0.48 (-0.55 to -0.4) |
| Western Sub-Saharan Africa | 974166 (694695-1306372) | 786.45 (559.82-1055.9) | 2419519  (1712962-3244286) | 770.61 (549.43-1039.03) | -0.07 (-0.1 to -0.03) |
| World Bank High Income | 13738380  (9832218-18464106) | 1203.64 (865.54-1620.43) | 18192260  (13150362-24264013) | 1126 (813.42-1505.14) | -0.15  (-0.17 to -0.13) |
| World Bank Low Income | 1846126 (1312041-2457737) | 866.17 (619.57-1163.09) | 4211018  (2985950-5614661) | 843.13 (601.48-1138.6) | -0.1 (-0.11 to -0.08) |
| World Bank Lower Middle Income | 12142414  (8653292-16236918) | 860.9 (615.2-1156.77) | 23710074  (16957142-31931173) | 783.83 (560.63-1051.98) | -0.29  (-0.33 to -0.24) |
| World Bank Upper Middle Income | 15506829  (11094416-20946910) | 854.71 (609.36-1154.08) | 23855049  (16952421-32168295) | 749.38 (535.85-1003.75) | -0.31  (-0.37 to -0.24) |

ASR, age-standardized rate; LBP, low back pain; UI, uncertainty interval, CI, confdence interval; EAPC, estimated annual percentage change; DALYs, disability-adjusted life years.
