# Supplementary material for: Epidemiological trends and geographic disparities in low back pain burden based on the 2021 GBD study: A cross-sectional analysis
Source: Medicine (Baltimore). 2026 Jun 12;105(24):e49201. doi: 10.1097/MD.0000000000049201 (PMC13268564; doi:10.1097/MD.0000000000049201)
Supplement: Supplementary file 15 [file medi-105-e49201-s015.docx]

Table S7. The DALYs cases and ASR for LBP in 204 countries and territories between 1990 and 2021, and its temporal trends.

| **Location** | **1990** |  | **2021** | **EAPC (95% CI) 1990-2021** |
| --- | --- | --- | --- | --- |
|  | **Cases (95% UI) ASR per 100 000 (95% UI)** | **Cases (95% UI)** | **ASR per 100 000 (95% UI)** |  |

| Global | 43386226  (31083937-58355210) | 937.34 (669.13-1261) | 70156962  (50194205-94104688) | 832.18 (595.85-1115.24) | -0.32 (-0.35 to -0.28) |
| --- | --- | --- | --- | --- | --- |
| Country |  |  |  |  |  |
| Afghanistan | 71876 (51040-96100) | 937.52 (671.26-1257.46) | 192329 (134797-258340) | 935.42 (667.77-1244.03) | 0 (-0.01-0.01) |
| Albania | 39825 (28329-53824) | 1450.55 (1037.29-1949.45) | 50119 (35701-67964) | 1465.12 (1042.24-1978.49) | 0.04 (0.03-0.04) |
| Algeria | 174203 (121908-233251) | 943.36 (677.67-1275.44) | 394980 (281805-531508) | 931.15 (667.12-1254.59) | -0.06 (-0.08 to -0.05) |
| American Samoa | 251 (177-338) | 735.44 (518.42-993.35) | 361 (259-486) | 713.99 (511.82-955.96) | -0.07 (-0.09 to -0.05) |
| Andorra | 655 (470-878) | 1070.06 (766.67-1427.3) | 1237 (871-1674) | 1034.3 (737.79-1388.95) | -0.09 (-0.1 to -0.08) |
| Angola | 55754 (39327-74024) | 880.39 (621.7-1178.21) | 168042 (118451-225847) | 841.77 (608.4-1126.17) | -0.16 (-0.19 to -0.12) |
| Antigua and Barbuda | 375 (267-501) | 673.71 (481.34-907.59) | 700 (501-939) | 666.76 (475.19-890.54) | -0.03 (-0.04 to -0.01) |
| Argentina | 355105 (252342-477353) | 1100.45 (782.75-1475.94) | 552287 (391178-742471) | 1086.54 (770.8-1460.14) | -0.03 (-0.07-0) |
| Armenia | 34414 (24373-46066) | 1112.25 (792.45-1489.87) | 41538 (29817-56107) | 1113.27 (803.07-1493.35) | 0.05 (0.03-0.06) |
| Australia | 252973 (179166-338769) | 1370.11 (969.96-1833.99) | 404050 (289168-547269) | 1255.82 (897.47-1688.59) | -0.21 (-0.24 to -0.18) |
| Austria | 93423 (67374-124427) | 977.3 (711.48-1301.82) | 113411 (80594-153070) | 909.12 (654.67-1219.52) | -0.06 (-0.12-0) |
| Azerbaijan | 61950 (44102-82673) | 1030.12 (728.49-1370.28) | 118188 (84354-160452) | 1041.16 (740.51-1404.64) | 0.09 (0.07-0.1) |
| Bahamas | 1465 (1041-1947) | 677.04 (478.26-912.38) | 2893 (2044-3917) | 670.25 (472.92-904.68) | -0.04 (-0.05 to -0.04) |
| Bahrain | 3719 (2551-5000) | 911.63 (644.11-1211.56) | 13994 (9818-18891) | 888.7 (637.63-1185.1) | -0.09 (-0.09 to -0.08) |
| Bangladesh | 765542 (546475-1020134) | 1083.42 (779.35-1447.71) | 1582987 (1135196-2130732) | 1003.98 (722.55-1347.79) | -0.18 (-0.25 to -0.11) |
| Barbados | 1823 (1305-2450) | 687.58 (493.46-926.32) | 2690 (1928-3645) | 677.01 (485.88-912.16) | -0.04 (-0.05 to -0.04) |
| Belarus | 145232 (103246-196117) | 1221.94 (872.13-1637.87) | 155738 (112242-207330) | 1214.75 (876.3-1627.36) | -0.01 (-0.02-0) |
| Belgium | 135081 (95948-182332) | 1101.37 (784.73-1493.45) | 164693 (116610-222326) | 1072.35 (768.79-1439.85) | -0.08 (-0.09 to -0.07) |
| Belize | 891 (627-1187) | 690.24 (486.4-933.28) | 2701 (1950-3640) | 688.95 (495.01-936.64) | 0.02 (0.01-0.04) |
| Benin | 23061 (16477-30829) | 809.83 (580.48-1094.24) | 65291 (45708-86804) | 779.45 (545.12-1046.31) | -0.13 (-0.17 to -0.1) |
| Bermuda | 462 (332-622) | 703.14 (508.48-943.89) | 640 (454-864) | 692.27 (500.45-924.2) | -0.06 (-0.06 to -0.05) |
| Bhutan | 4000 (2825-5346) | 987.63 (711.63-1324.45) | 7071 (5065-9474) | 969.88 (695.08-1294.5) | -0.04 (-0.05 to -0.03) |
| Bolivia | 31297 (22283-42021) | 681.03 (484.56-920.08) | 75178 (53099-99696) | 686.44 (486.31-916.61) | 0.01 (-0.03-0.05) |
| Bosnia and Herzegovina | 61426 (43719-82066) | 1333.64 (956.33-1782.22) | 62599 (45076-83829) | 1372.37 (984.17-1838.16) | 0.11 (0.09-0.12) |
| Botswana | 5891 (4190-7792) | 727.21 (513.26-973.37) | 14506 (10259-19479) | 714.69 (514.28-956.16) | -0.02 (-0.04 to -0.01) |
| Brazil | 1239399  (881976-1657077) | 1002.4 (715.13-1344.89) | 2539065 (1819608-3418673) | 1034.16 (744.86-1387.84) | 0.08 (0.05-0.1) |
| Brunei Darussalam | 2102 (1465-2825) | 1017.13 (730.07-1374.63) | 4646 (3301-6220) | 972.16 (690.57-1302.37) | -0.13 (-0.15 to -0.11) |
| Bulgaria | 150147 (107652-202730) | 1415.56 (1020.14-1894.13) | 135150 (96976-181613) | 1384.98 (989.34-1861.48) | -0.04 (-0.05 to -0.04) |
| Burkina Faso | 46458 (32754-61933) | 800.79 (570.55-1078.02) | 111868 (79167-150637) | 785.07 (570.92-1068.36) | -0.11 (-0.14 to -0.08) |
| Burundi | 29944 (21345-40059) | 890.09 (637.06-1201.52) | 69173 (48749-92368) | 841.33 (598.81-1124.49) | -0.21 (-0.22 to -0.2) |
| Cabo Verde | 1874 (1328-2498) | 773.46 (542.32-1045.71) | 3860 (2732-5211) | 721.26 (515.48-976.82) | -0.25 (-0.29 to -0.21) |
| Cambodia | 48876 (35114-65214) | 746.24 (534.26-1001.73) | 105508 (74404-140804) | 692.21 (492.69-928.92) | -0.25 (-0.27 to -0.24) |
| Cameroon | 54889 (38351-73369) | 843.27 (593.96-1140.43) | 167339 (118112-225928) | 803.38 (571.44-1087.33) | -0.17 (-0.21 to -0.14) |
| Canada | 332779 (235195-445252) | 1086.6 (769.43-1450.19) | 483677 (344637-660082) | 990.93 (705.75-1328.01) | -0.21 (-0.24 to -0.18) |
| Central African Republic | 14851 (10621-19728) | 853.85 (620.59-1132.86) | 31005 (22022-41247) | 846.45 (605.26-1130.73) | -0.04 (-0.05 to -0.03) |

| Chad | 34899 (25016-47157) | 930.8 (662.63-1258.23) | 83842 (58674-110863) | 853.39 (607.2-1140.46) | -0.24 (-0.33 to -0.16) |
| --- | --- | --- | --- | --- | --- |
| Chile | 134995 (96082-180167) | 1108.18 (792.09-1482.72) | 245014 (173292-329867) | 1097.66 (779.01-1480.04) | -0.05 (-0.08 to -0.01) |
| China | 7772958  (5520145-10545676) | 749.03 (530.01-1013.84) | 11297805  (7931468-15328056) | 603.03 (427.63-810.16) | -0.49 (-0.6 to -0.39) |
| Colombia | 239197 (168594-319921) | 909.01 (647.26-1215.22) | 493600 (347216-661959) | 916.32 (645.9-1226.5) | 0.07 (0.04-0.09) |
| Comoros | 2269 (1619-3026) | 790.53 (567.98-1053.96) | 4982 (3591-6639) | 797.8 (578.73-1064.13) | -0.02 (-0.05-0) |
| Congo | 12508 (8847-16661) | 807.44 (575.44-1074.5) | 33326 (23525-44929) | 802.87 (571.11-1084.19) | -0.04 (-0.05 to -0.02) |
| Cook Islands | 111 (79-149) | 721.99 (513.02-975.84) | 160 (113-217) | 734.12 (519.82-992.78) | 0.1 (0.08-0.11) |
| Costa Rica | 21323 (15051-28586) | 869.84 (620.43-1166.21) | 44770 (32024-60052) | 843.89 (603.9-1128.09) | -0.08 (-0.09 to -0.08) |
| Croatia | 77873 (55832-103325) | 1369.97 (987.74-1808.92) | 80639 (57456-108284) | 1346.91 (961.41-1812.34) | -0.01 (-0.07-0.05) |
| Cuba | 75970 (54505-102623) | 691.58 (494.03-938.56) | 97908 (69968-129870) | 645.41 (465.92-847.55) | -0.09 (-0.13 to -0.05) |
| Cyprus | 8848 (6240-12124) | 1081.42 (761.17-1475.52) | 18417 (13136-24993) | 1069.96 (768.31-1437.91) | -0.06 (-0.08 to -0.04) |
| Czechia | 185222 (131803-247476) | 1534.77 (1091.22-2045.93) | 219883 (157882-295511) | 1488.41 (1068.88-2002.2) | -0.09 (-0.1 to -0.09) |
| Côte d'Ivoire | 59192 (42031-78814) | 817.96 (587.68-1083.26) | 150384 (106075-203961) | 799.33 (570.49-1086.16) | -0.06 (-0.11 to -0.02) |
| Democratic People's Republic of Korea | 155361 (109559-209162) | 820.1 (578.5-1110.88) | 247560 (173494-333060) | 771.93 (541.25-1033.12) | -0.22 (-0.24 to -0.2) |
| Democratic Republic of the Congo | 204118 (144026-274757) | 866.28 (625.15-1169.22) | 503220 (359172-677111) | 847.33 (608.45-1144.54) | -0.09 (-0.11 to -0.06) |
| Denmark | 93137 (65818-121968) | 1458.42 (1030.23-1916.67) | 100854 (71492-137039) | 1276.55 (891.31-1730.95) | -0.7 (-0.83 to -0.56) |
| Djibouti | 2019 (1439-2721) | 798.55 (571.08-1068.1) | 7540 (5330-10170) | 753.34 (536.13-1015.59) | -0.21 (-0.22 to -0.2) |
| Dominica | 443 (315-594) | 708.04 (508.32-948.68) | 527 (380-706) | 678.83 (491.29-903.19) | -0.16 (-0.17 to -0.15) |
| Dominican Republic | 36605 (25924-49243) | 673.85 (483.59-897.35) | 75554 (53994-101485) | 691.71 (495.57-930.73) | 0.08 (0.05-0.1) |
| Ecuador | 49048 (34823-64964) | 651.61 (465.82-871.94) | 107611 (76764-143498) | 610.22 (434.75-815.8) | -0.22 (-0.33 to -0.12) |
| Egypt | 396140 (278915-535292) | 943.71 (670.42-1267.08) | 866670 (611823-1160825) | 964.52 (697.67-1290.44) | 0.09 (0.05-0.13) |
| El Salvador | 33850 (23847-45608) | 844.48 (599.47-1140.34) | 54833 (39234-73801) | 865.98 (619.19-1164.41) | 0.14 (0.12-0.15) |
| Equatorial Guinea | 2306 (1651-3078) | 853.52 (609.48-1140.03) | 7988 (5601-10652) | 815.2 (587.33-1087.3) | -0.17 (-0.19 to -0.15) |
| Eritrea | 14552 (10372-19347) | 745.61 (533.09-1001.92) | 34196 (24297-45471) | 754.28 (536.42-1008.71) | 0.08 (0.06-0.09) |
| Estonia | 22244 (15806-29980) | 1205.94 (857.45-1629.86) | 22599 (16134-30164) | 1194.77 (857.26-1601.87) | 0.01 (-0.01-0.04) |
| Eswatini | 2976 (2135-3979) | 659.33 (472.83-889.84) | 5284 (3793-7049) | 639.2 (456.72-853.76) | -0.15 (-0.19 to -0.11) |
| Ethiopia | 265081 (187189-353727) | 884.45 (632.09-1180.21) | 582836 (412379-778457) | 822.39 (583.75-1105.62) | -0.22 (-0.24 to -0.21) |
| Fiji | 4042 (2876-5362) | 725.03 (516.19-973.57) | 6135 (4348-8205) | 695.3 (489.51-928.93) | -0.12 (-0.13 to -0.12) |
| Finland | 59803 (42338-80105) | 981.53 (698.25-1313.38) | 73349 (51555-99318) | 938.73 (660.92-1269.84) | -0.1 (-0.12 to -0.08) |
| France | 724568 (525420-973795) | 1071.67 (774.65-1445.43) | 951532 (681052-1270497) | 1071.05 (771.19-1433.02) | -0.02 (-0.04-0) |
| Gabon | 5607 (3970-7514) | 797.13 (567.01-1072.09) | 11395 (8125-15264) | 792.72 (566.79-1061.33) | -0.01 (-0.01-0) |
| Gambia | 4331 (3055-5773) | 748.38 (531.64-1004.42) | 11353 (8121-15140) | 724.18 (519.32-967.79) | -0.11 (-0.15 to -0.07) |
| Georgia | 58207 (41396-78536) | 974.92 (697.24-1311.92) | 45020 (32127-60305) | 955.37 (686.64-1273.16) | -0.15 (-0.19 to -0.1) |
| Germany | 1323936  (943240-1797301) | 1312.59 (935.51-1781.99) | 1497385 (1067933-2000007) | 1242.99 (887.88-1668.43) | -0.11 (-0.13 to -0.08) |
| Ghana | 69642 (49281-93213) | 735.56 (522.55-984.76) | 175751 (124275-234096) | 697.19 (500.4-921.37) | -0.2 (-0.23 to -0.17) |
| Greece | 128149 (89895-171400) | 1020.65 (724.25-1367.05) | 148243 (106019-197893) | 1012.27 (734.38-1350.32) | -0.06 (-0.09 to -0.03) |
| Greenland | 540 (382-727) | 1017.62 (729.35-1353.07) | 631 (443-850) | 966.25 (692.13-1294.26) | -0.1 (-0.13 to -0.07) |
| Grenada | 492 (346-654) | 686.77 (489.28-917.65) | 770 (544-1036) | 674.08 (480.12-906.28) | -0.04 (-0.06 to -0.03) |
| Guam | 809 (567-1089) | 720.76 (508.91-978.14) | 1325 (944-1765) | 712.29 (508.36-953.84) | -0.02 (-0.03 to -0.01) |
| Guatemala | 54938 (38767-72920) | 993.07 (710.9-1332.79) | 133095 (93889-179558) | 946.74 (667.11-1267.22) | -0.09 (-0.13 to -0.04) |

| Guinea | 33029 (23391-44535) | 806.82 (576.1-1086.46) | 68255 (48445-91335) | 798.21 (573.47-1061.45) | -0.08 (-0.12 to -0.03) |
| --- | --- | --- | --- | --- | --- |
| Guinea-Bissau | 4620 (3277-6168) | 767.92 (548.54-1047.1) | 9748 (6866-13099) | 756.12 (535.56-1019.5) | -0.08 (-0.11 to -0.04) |
| Guyana | 3934 (2795-5188) | 672.87 (483.56-896.22) | 4900 (3513-6548) | 663.94 (480.87-885.87) | -0.04 (-0.04 to -0.04) |
| Haiti | 31028 (21725-41783) | 673.74 (479.05-911.42) | 71355 (51465-95405) | 667.35 (487.35-899.05) | -0.05 (-0.07 to -0.02) |
| Honduras | 26982 (18855-36464) | 852.64 (603.21-1157.56) | 75707 (53970-101067) | 863.62 (626.95-1158.24) | 0.07 (0.06-0.08) |
| Hungary | 198912 (142680-267017) | 1593.19 (1145.94-2126.47) | 212508 (151934-283251) | 1573.52 (1118.61-2089.53) | -0.03 (-0.03 to -0.02) |
| Iceland | 3092 (2173-4186) | 1161.52 (816.92-1568.72) | 4734 (3413-6384) | 1089.89 (790.97-1466.73) | -0.24 (-0.25 to -0.22) |
| India | 5303845  (3799937-7088637) | 824.3 (593.59-1106.51) | 9780012 (7003903-13127349) | 713.88 (509.42-955.04) | -0.48 (-0.6 to -0.36) |
| Indonesia | 994739 (703409-1332230) | 715.88 (510.71-964.32) | 1985798 (1402418-2680164) | 697.12 (496.56-936.17) | -0.03 (-0.05 to -0.01) |
| Iran | 451695 (316798-606966) | 1131.51 (810.92-1522.57) | 923962 (657330-1239276) | 1027.98 (740.46-1370.26) | -0.25 (-0.3 to -0.2) |
| Iraq | 119579 (83975-159365) | 941.41 (674.42-1261.15) | 326494 (231950-441521) | 918.4 (660.53-1236) | -0.07 (-0.08 to -0.06) |
| Ireland | 41688 (29820-55596) | 1112.6 (804.77-1483.65) | 67700 (48134-91490) | 1105.55 (795.89-1485.7) | -0.1 (-0.13 to -0.07) |
| Israel | 54881 (38597-74176) | 1148.62 (811.6-1553.03) | 113007 (81286-151345) | 1099.3 (792.66-1477.37) | -0.12 (-0.15 to -0.09) |
| Italy | 775975 (551918-1042169) | 1088.79 (783.99-1463.08) | 952372 (674066-1281968) | 1082.24 (773.35-1455.86) | -0.08 (-0.09 to -0.06) |
| Jamaica | 13915 (10030-18616) | 704.59 (504.74-942.93) | 21509 (15212-29077) | 699.91 (494.96-947.98) | -0.04 (-0.06 to -0.02) |
| Japan | 1974322  (1399798-2673927) | 1313.43 (938.44-1765.7) | 2232624 (1590996-2995120) | 1208.31 (861.08-1627.71) | -0.2 (-0.23 to -0.17) |
| Jordan | 24193 (16995-32606) | 957.83 (680.14-1277.69) | 104643 (74776-141394) | 938.81 (675.77-1252.94) | -0.05 (-0.06 to -0.05) |
| Kazakhstan | 153008 (109239-203416) | 1061.83 (761.95-1407.37) | 202892 (145658-274065) | 1061.69 (762.69-1425.21) | -0.02 (-0.05-0) |
| Kenya | 120635 (85397-160365) | 923.01 (659.36-1242.2) | 319038 (226680-426821) | 898.52 (641.5-1209.83) | -0.07 (-0.09 to -0.05) |
| Kiribati | 379 (268-509) | 724.11 (519.44-976.38) | 730 (516-974) | 746.76 (528.15-990.85) | 0.18 (0.15-0.21) |
| Kuwait | 12624 (8810-16988) | 901.49 (642.91-1194.89) | 46747 (32727-62330) | 933.59 (656.38-1248.03) | 0.12 (0.11-0.14) |
| Kyrgyzstan | 37288 (26572-50175) | 1070.49 (765.13-1448.33) | 61447 (44570-82294) | 1034.97 (748.9-1394.38) | -0.1 (-0.11 to -0.09) |
| Lao People's Democratic Republic | 19072 (13572-25297) | 683.44 (490.93-913.11) | 40834 (29353-54784) | 652.12 (465.49-875.89) | -0.16 (-0.18 to -0.14) |
| Latvia | 37779 (26816-50234) | 1190.66 (852.1-1583.42) | 32434 (23142-43017) | 1173 (834.75-1566.08) | -0.04 (-0.05 to -0.03) |
| Lebanon | 23889 (16924-31812) | 923.72 (657.86-1231.36) | 54839 (39417-73331) | 917.86 (661.62-1228.63) | 0.05 (0.03-0.07) |
| Lesotho | 7796 (5601-10468) | 746.71 (533.6-1001.93) | 9952 (7153-13239) | 693.96 (496.87-927.91) | -0.29 (-0.31 to -0.26) |
| Liberia | 12015 (8509-15946) | 761.82 (541.21-1017.7) | 27616 (19600-36844) | 740.26 (536.48-993.84) | -0.11 (-0.12 to -0.1) |
| Libya | 28269 (20147-38014) | 936.89 (672.34-1254.35) | 65780 (46473-87418) | 927.25 (662.63-1239.13) | -0.05 (-0.06 to -0.04) |
| Lithuania | 50757 (36227-68287) | 1219.55 (878.72-1641.15) | 47889 (34138-63555) | 1191.04 (848.63-1584.12) | -0.09 (-0.1 to -0.07) |
| Luxembourg | 5145 (3680-6899) | 1105.28 (793.62-1478.32) | 8945 (6345-12128) | 1075.22 (763.67-1449.56) | -0.1 (-0.11 to -0.09) |
| Madagascar | 66350 (46860-88088) | 899.23 (645.96-1208.38) | 157542 (109891-213014) | 839.3 (597.59-1133.11) | -0.25 (-0.28 to -0.22) |
| Malawi | 49928 (35693-66093) | 852.77 (624.46-1122.3) | 103907 (72944-138488) | 845.6 (598.06-1145.23) | -0.03 (-0.05 to -0.02) |
| Malaysia | 84880 (60631-111150) | 633.49 (452.96-837.88) | 198083 (141269-268391) | 615.65 (441.54-834.04) | -0.17 (-0.22 to -0.12) |
| Maldives | 809 (575-1080) | 594.51 (423.61-799.4) | 2954 (2067-3978) | 564.98 (396.39-750.07) | -0.16 (-0.2 to -0.12) |
| Mali | 37185 (26399-49289) | 678.85 (482.01-897.41) | 99569 (70866-132169) | 705.65 (506.25-941.94) | 0.09 (0.07-0.12) |
| Malta | 4659 (3318-6308) | 1142.63 (818.05-1540.66) | 7108 (5049-9556) | 1136.64 (823.84-1525.48) | -0.09 (-0.12 to -0.06) |
| Marshall Islands | 185 (130-245) | 700.97 (494.14-939.19) | 326 (228-441) | 672.29 (476.86-903.54) | -0.14 (-0.14 to -0.13) |
| Mauritania | 9527 (6745-12737) | 707.29 (505.11-954.17) | 21421 (15225-28520) | 710.03 (499.49-957.27) | -0.05 (-0.08 to -0.01) |
| Mauritius | 6464 (4614-8747) | 684.52 (492.41-918.49) | 10431 (7487-14145) | 640.48 (457.81-865.16) | -0.17 (-0.21 to -0.12) |
| Mexico | 503907 (356499-676782) | 777.61 (554.24-1048.23) | 1085530 (772664-1462111) | 801.94 (572.56-1081.9) | 0.08 (-0.03-0.19) |
| Micronesia | 498 (360-664) | 737.25 (530.16-985.86) | 675 (474-907) | 735.97 (521.25-983.86) | 0.02 (0-0.04) |

| Monaco | 479 (338-650) | 1079.06 (772.34-1450.87) | 591 (416-798) | 1036.84 (741.18-1397.53) | -0.12 (-0.12 to -0.11) |
| --- | --- | --- | --- | --- | --- |
| Mongolia | 15209 (10831-20209) | 1044.56 (740.93-1401.87) | 30156 (21520-40698) | 1012.29 (730.65-1363.32) | -0.12 (-0.13 to -0.11) |
| Montenegro | 9114 (6509-12374) | 1413.07 (1014.22-1906.99) | 11231 (7954-15170) | 1422.67 (1010.29-1912.61) | 0.06 (0.04-0.07) |
| Morocco | 216947 (153332-289054) | 1089.72 (787.16-1460.3) | 399143 (283978-533308) | 1060.29 (756.26-1416.19) | -0.05 (-0.15-0.06) |
| Mozambique | 75773 (54045-100283) | 888.72 (641.16-1178.69) | 162939 (115077-218833) | 880.34 (634.62-1181.94) | 0 (-0.02-0.01) |
| Myanmar | 170699 (123112-226633) | 560.27 (404.3-743.96) | 308676 (217558-409158) | 565.48 (396.38-750.32) | -0.01 (-0.04-0.03) |
| Namibia | 6750 (4806-8992) | 752.93 (540.53-1006.87) | 14038 (9970-18733) | 745.7 (532.92-1003.14) | 0.02 (0-0.04) |
| Nauru | 51 (36-68) | 721.07 (516.51-970.86) | 62 (44-84) | 740.86 (528.75-996.74) | 0.12 (0.09-0.14) |
| Nepal | 171798 (120761-228514) | 1267.96 (900.2-1701.95) | 321833 (227535-435105) | 1150.42 (821.7-1560.96) | -0.23 (-0.32 to -0.13) |
| Netherlands | 168579 (121731-224795) | 966.01 (698.97-1282.7) | 221249 (157277-296044) | 935.36 (669.04-1256.8) | -0.06 (-0.12 to -0.01) |
| New Zealand | 51888 (37039-69437) | 1410.49 (1007.95-1887.74) | 84337 (60144-113925) | 1331.3 (951.02-1787.55) | -0.13 (-0.15 to -0.11) |
| Nicaragua | 22365 (15773-29798) | 874.61 (624.91-1174.44) | 53552 (38189-71749) | 864.25 (620.38-1156.99) | 0 (-0.01-0.02) |
| Niger | 35009 (25129-46637) | 770.2 (552.83-1033.05) | 108264 (76336-144761) | 794.97 (561.03-1061.63) | 0.16 (0.13-0.2) |
| Nigeria | 475284 (339894-635043) | 788.34 (561.27-1057.08) | 1145003 (813020-1532827) | 779.59 (553.38-1051.55) | -0.02 (-0.06-0.02) |
| Niue | 15 (11-21) | 726.59 (516.95-978.09) | 14 (10-19) | 710.8 (506.44-947.97) | -0.05 (-0.06 to -0.04) |
| North Macedonia | 26436 (18911-35576) | 1309.9 (940.46-1755.04) | 37104 (26145-50264) | 1299.76 (924.62-1753.5) | 0 (-0.01-0.01) |
| Northern Mariana Islands | 265 (184-364) | 719.66 (506.25-983.78) | 392 (278-533) | 711.05 (506.69-962.94) | -0.05 (-0.08 to -0.03) |
| Norway | 54496 (38998-73201) | 1055.17 (758.41-1420.77) | 70985 (50389-95633) | 997.68 (714.29-1351.09) | -0.2 (-0.21 to -0.18) |
| Oman | 12689 (8924-17126) | 903.19 (643.13-1208.98) | 38190 (27011-51442) | 885.36 (635.05-1175.23) | -0.05 (-0.06 to -0.05) |
| Pakistan | 583257 (410997-788716) | 781.04 (553.26-1059.82) | 1555725 (1091574-2118767) | 861 (604.05-1170.82) | 0.44 (0.36-0.51) |
| Palau | 92 (65-124) | 715.09 (511.12-965.56) | 158 (112-216) | 692.44 (493.09-929.83) | -0.08 (-0.1 to -0.06) |
| Palestine | 13020 (9096-17412) | 967.73 (693.19-1301.39) | 38117 (26850-50891) | 928.5 (662.2-1249.64) | -0.12 (-0.13 to -0.11) |
| Panama | 16639 (11723-22230) | 833.85 (593.15-1120.31) | 36829 (26283-49125) | 837.5 (599.55-1116.35) | 0.03 (0.01-0.04) |
| Papua New Guinea | 19483 (13861-26143) | 701.9 (498.24-937.1) | 54745 (38935-73757) | 697.95 (500.13-935.46) | 0.01 (-0.01-0.03) |
| Paraguay | 25523 (18243-33978) | 835.57 (595.64-1111.12) | 59709 (42402-79967) | 868.92 (617.82-1170.43) | 0.12 (0.05-0.19) |
| Peru | 104684 (73842-139719) | 634.14 (447.75-845.22) | 237038 (167052-317742) | 652.64 (458.37-876.65) | 0.16 (0.13-0.19) |
| Philippines | 306672 (218322-411477) | 695.44 (493.09-936.64) | 684247 (484100-923176) | 678.85 (481.55-914.75) | -0.11 (-0.13 to -0.09) |
| Poland | 609194 (436923-820031) | 1466 (1048.46-1969.5) | 749888 (533070-1011310) | 1426.76 (1014.51-1919.36) | -0.1 (-0.11 to -0.09) |
| Portugal | 138656 (99000-185019) | 1165.15 (835.49-1551.67) | 176578 (125999-237117) | 1144.57 (814.2-1538.15) | -0.12 (-0.15 to -0.08) |
| Puerto Rico | 24601 (17334-32870) | 676.15 (476.84-903.68) | 31312 (22118-42091) | 672.68 (480.9-904. 16) | -0.03 (-0.05 to -0.02) |
| Qatar | 3303 (2263-4472) | 880.9 (626.08-1180.55) | 27002 (18950-36884) | 895.34 (644.16-1193.12) | 0 (-0.01-0.02) |
| Republic of Korea | 461880 (326978-619749) | 1095.85 (796.8-1464.34) | 727108 (516989-977658) | 1020.94 (729.04-1368.32) | -0.21 (-0.23 to -0.19) |
| Republic of Moldova | 54453 (38892-73693) | 1211.99 (861.38-1635.92) | 58752 (41950-79857) | 1191.87 (855.6-1607.42) | -0.09 (-0.11 to -0.07) |
| Romania | 397941 (281793-536044) | 1529.05 (1085.41-2050.83) | 381364 (271281-512846) | 1453.03 (1034.87-1959.19) | -0.21 (-0.23 to -0.19) |
| Russian Federation | 2139198  (1531728-2879390) | 1254.61 (899.04-1690.28) | 2352957 (1685540-3172235) | 1206.26 (866.68-1623.31) | -0.02 (-0.05-0.01) |
| Rwanda | 39816 (27725-53196) | 930.51 (665.67-1255.89) | 86063 (61046-115707) | 904.53 (642.64-1219.37) | -0.11 (-0.12 to -0.09) |
| Saint Kitts and Nevis | 249 (175-335) | 680.96 (482.25-909.63) | 468 (335-632) | 663.34 (477.14-896.17) | -0.09 (-0.09 to -0.09) |
| Saint Lucia | 771 (546-1035) | 718.46 (509.33-965.08) | 1497 (1070-2007) | 683.54 (493.6-915.61) | -0.16 (-0.16 to -0.16) |
| Saint Vincent and the Grenadines | 583 (412-791) | 676.93 (481.8-916.78) | 867 (620-1170) | 661.28 (477.03-893.51) | -0.06 (-0.07 to -0.06) |
| Samoa | 881 (625-1181) | 772.31 (555.07-1037.16) | 1249 (892-1669) | 726.72 (518.93-970.71) | -0.25 (-0.29 to -0.22) |
| San Marino | 313 (224-418) | 1083.81 (776.5-1444.22) | 488 (344-659) | 1041.65 (745.61-1398.2) | -0.12 (-0.13 to -0.11) |
| Sao Tome and Principe | 551 (392-735) | 700.6 (499.6-946.57) | 1120 (788-1510) | 679.24 (475.29-916.34) | -0.1 (-0.12 to -0.09) |

| Saudi Arabia | 101266 (71515-135749) | 888.35 (629.09-1191.43) | 344324 (238493-460839) | 906.9 (645.76-1204.63) | 0.09 (0.08-0.1) |
| --- | --- | --- | --- | --- | --- |
| Senegal | 33424 (23996-44640) | 727.16 (514.52-972.49) | 77876 (56287-103686) | 701.25 (504.41-939.38) | -0.18 (-0.21 to -0.15) |
| Serbia | 156661 (111958-210310) | 1442.89 (1043.87-1926.01) | 174204 (124316-234842) | 1443.06 (1031.71-1935.49) | 0.01 (0-0.01) |
| Seychelles | 396 (280-533) | 636.65 (447.6-857.7) | 714 (510-960) | 596.72 (430.56-798.09) | -0.21 (-0.22 to -0.21) |
| Sierra Leone | 22229 (15853-29830) | 800.77 (574.03-1082.4) | 45217 (31818-60773) | 757.03 (534.97-1015.89) | -0.18 (-0.2 to -0.16) |
| Singapore | 28325 (20069-37680) | 916.28 (655.97-1223.16) | 63350 (44460-85507) | 847.08 (603.31-1134.9) | -0.13 (-0.18 to -0.07) |
| Slovakia | 84309 (60264-113283) | 1484.19 (1062.27-1986.01) | 103908 (74393-141133) | 1421.02 (1015.78-1920.78) | -0.12 (-0.13 to -0.1) |
| Slovenia | 30135 (21418-40189) | 1322.39 (942.99-1757.63) | 38585 (27465-51728) | 1302.04 (925.16-1744.64) | -0.06 (-0.08 to -0.04) |
| Solomon Islands | 1471 (1045-1973) | 700.97 (499.21-952.68) | 3808 (2684-5079) | 747.37 (531.64-1000.84) | 0.29 (0.26-0.32) |
| Somalia | 37263 (26233-49535) | 842.71 (602.52-1122.08) | 100637 (71402-134951) | 854.48 (610.55-1153.54) | 0.03 (0.01-0.04) |
| South Africa | 203699 (146422-272076) | 758.6 (540.03-1018.52) | 375306 (268811-505397) | 693.42 (497.79-927.6) | -0.25 (-0.26 to -0.23) |
| South Sudan | 30078 (21484-40046) | 814.22 (583.9-1093.01) | 50114 (35405-66764) | 810.06 (571.77-1078.3) | -0.02 (-0.04 to -0.01) |
| Spain | 422933 (300741-556185) | 926.49 (663.12-1219.2) | 542892 (383938-733436) | 841.91 (597.12-1116.99) | -0.06 (-0.18-0.05) |
| Sri Lanka | 90233 (64128-120903) | 634.71 (452.16-850.99) | 159140 (113756-214997) | 627.37 (448.55-841.75) | -0.04 (-0.06 to -0.02) |
| Sudan | 135212 (94903-181113) | 954.81 (679.93-1277.93) | 317383 (222150-424063) | 948.08 (686.86-1259.05) | -0.01 (-0.02-0) |
| Suriname | 2194 (1552-2952) | 675.69 (477.39-911.21) | 4273 (3042-5754) | 682.63 (486.16-919.22) | 0.06 (0.05-0.08) |
| Sweden | 74608 (52973-98630) | 660.9 (470.57-879.39) | 113838 (80719-154383) | 802.64 (571.03-1090.96) | 0.62 (0.47-0.78) |
| Switzerland | 104615 (76068-136848) | 1262.16 (917.81-1655.13) | 137762 (97949-184244) | 1133.67 (805-1518.45) | -0.15 (-0.28 to -0.01) |
| Syrian Arab Republic | 83089 (58812-111908) | 972.42 (696.03-1315.59) | 137236 (97764-185525) | 957.43 (683.15-1282.41) | -0.06 (-0.07 to -0.05) |
| Taiwan (Province of China) | 151939 (109677-199314) | 794.14 (578.01-1036.09) | 322191 (240127-419803) | 934.38 (692.61-1222.08) | 0.64 (0.54-0.73) |
| Tajikistan | 36067 (25431-48317) | 1005.75 (710.79-1356.67) | 78287 (55786-104647) | 966.11 (682.79-1306.79) | -0.13 (-0.14 to -0.13) |
| Thailand | 264616 (185676-354955) | 553.11 (393.13-737.34) | 534708 (377829-729711) | 581.13 (413.21-789.1) | 0.23 (0.15-0.31) |
| Timor-Leste | 3151 (2212-4234) | 626.94 (443.82-839.33) | 6351 (4516-8477) | 598.77 (425.49-801.44) | -0.16 (-0.19 to -0.12) |
| Togo | 16916 (11845-22722) | 810.52 (571.49-1105.72) | 45713 (32252-60904) | 768.44 (546.32-1029.04) | -0.21 (-0.26 to -0.16) |
| Tokelau | 10 (7-13) | 726.28 (512.28-983.97) | 10 (7-14) | 710.84 (510.49-956.07) | -0.04 (-0.05 to -0.03) |
| Tonga | 529 (373-716) | 761.47 (544.12-1036.83) | 653 (468-883) | 735.74 (529.52-995.53) | -0.14 (-0.15 to -0.12) |
| Trinidad and Tobago | 7131 (5115-9491) | 688.83 (496.66-922.22) | 11488 (8290-15370) | 678.18 (489.74-903.24) | -0.03 (-0.04 to -0.03) |
| Tunisia | 63612 (45294-85243) | 945.03 (676.26-1267.19) | 124587 (89855-167508) | 956.96 (692.22-1281.08) | 0.03 (0.02-0.05) |
| Turkmenistan | 26562 (18992-35469) | 1034.36 (748.95-1383.46) | 47674 (34011-64016) | 1002.04 (715.78-1341.61) | -0.08 (-0.09 to -0.07) |
| Tuvalu | 58 (41-79) | 743.82 (530.35-1003.76) | 83 (60-112) | 722.19 (517.92-974.61) | -0.05 (-0.07 to -0.03) |
| Türkiye | 482117 (347459-634220) | 1017.07 (735.78-1352.19) | 915900 (651279-1235554) | 991.64 (708.1-1330.33) | 0 (-0.08-0.07) |
| Uganda | 86617 (61362-115681) | 880.33 (625.02-1190.84) | 217602 (153887-289302) | 860.36 (620.38-1149.43) | -0.07 (-0.09 to -0.06) |
| Ukraine | 904082 (648401-1219303) | 1431.46 (1018.29-1915.15) | 837691 (598526-1128216) | 1372.69 (983.05-1829.59) | -0.1 (-0.13 to -0.06) |
| United Arab Emirates | 12043 (8269-16106) | 802.66 (570.51-1067.67) | 89867 (63618-122253) | 824.18 (588.58-1101.14) | 0.05 (0.01-0.09) |
| United Kingdom | 723836 (517492-969674) | 1062.56 (762.87-1424.91) | 951935 (673695-1278999) | 1062.95 (761.91-1432.1) | 0.15 (0.09-0.2) |
| United Republic of Tanzania | 134298 (95805-181888) | 855.43 (616.03-1155.26) | 325339 (230445-435865) | 836.38 (597.96-1130.65) | -0.04 (-0.06 to -0.02) |
| United States of America | 3610495  (2585813-4807828) | 1279.74 (919.09-1708.34) | 4940706 (3587048-6412519) | 1179.08 (856.96-1536.41) | -0.1 (-0.16 to -0.03) |
| United States Virgin Islands | 691 (488-924) | 683.92 (486.3-918.97) | 830 (585-1116) | 676.6 (488.41-901.07) | -0.03 (-0.04 to -0.01) |
| Uruguay | 34740 (24923-46320) | 1029.01 (740.2-1370.69) | 45929 (32889-61955) | 1106.93 (790.61-1483.09) | 0.2 (0.15-0.26) |
| Uzbekistan | 154269 (111041-204875) | 1046.07 (747.58-1401.46) | 327637 (232475-443183) | 1028.54 (729.41-1385.63) | 0 (-0.02-0.02) |
| Vanuatu | 753 (531-1009) | 772.1 (550.36-1042.12) | 1887 (1338-2545) | 784.89 (558.63-1052.44) | 0.06 (0.02-0.09) |
| Venezuela (Bolivarian | 119074 (83406-159216) | 808.84 (577.48-1094.02) | 226670 (163150-306182) | 780.58 (560.54-1049.6) | -0.13 (-0.14 to -0.11) |

| Republic of) |  |  |  |  |  |
| --- | --- | --- | --- | --- | --- |
| Viet Nam | 332523 (238427-443286) | 674.96 (488.3-903.35) | 713856 (510682-964555) | 668.72 (476.11-901.6) | 0.04 (0-0.07) |
| Yemen | 82183 (57910-110803) | 980.24 (701.77-1316.88) | 234199 (164680-311282) | 940.38 (661.96-1251.62) | -0.16 (-0.17 to -0.15) |
| Zambia | 31781 (22745-42574) | 702.43 (497.82-931.85) | 93908 (67285-124668) | 769.13 (554.65-1023.92) | 0.16 (0.11-0.22) |
| Zimbabwe | 46650 (33235-61877) | 772.86 (551.64-1030.23) | 85949 (60967-114996) | 820.06 (583.4-1105.89) | 0.25 (0.24-0.27) |

ASR, age-standardized rate; LBP, low back pain; UI, uncertainty interval, CI, confdence interval; EAPC, estimated annual percentage change; DALYs, disability-adjusted life years.
